# Antibody-mediated neutralization of myelin-associated EphrinB3 accelerates CNS remyelination

**DOI:** 10.1007/s00401-015-1521-1

**Published:** 2015-12-19

**Authors:** Yasir A. Syed, Chao Zhao, Don Mahad, Wiebke Möbius, Friedrich Altmann, Franziska Foss, Aycan Sentürk, Amparo Acker-Palmer, Gert Lubec, Kathryn Lilley, Robin J. M. Franklin, Klaus-A. Nave, Mark R. N. Kotter

**Affiliations:** 1 Wellcome Trust and MRC Cambridge Stem Cell Institute, Department of Clinical Neurosciences, Anne McLaren Laboratory, University of Cambridge, Cambridge, CB2 0SZ UK; 2 Centre for Neuroregeneration, Chancellor’s Building, 49 Little France Crescent, Edinburgh, EH16 4SB UK; 3 Department of Neurogenetics, Max Planck Institute for Experimental Medicine, 37075 Goettingen, Germany; 4 Department of Chemistry, University of Natural Resource and Life Sciences, Muthgasse 18, 1190 Vienna, Austria; 5 Frankfurt Institute for Molecular Life Sciences and Institute of Cell Biology and Neuroscience, Goethe University Frankfurt, Max-von-Laue-Str. 9, 60438 Frankfurt am Main, Germany; 6 Department of Pediatrics, Medical University Vienna, Waehringer Guertel 18-20, 1090 Vienna, Austria; 7 Cambridge Centre for Proteomics, Department of Biochemistry, University of Cambridge, Tennis Court Road, Cambridge, CB2 1QW UK; 8 Universitätsmedizin Göttingen, Universitätsklinik für Neurochirurgie, Robert-Koch-Straße 40, 37075 Göttingen, Germany; 9 Department of Pharmaceutical Chemistry, University of Vienna, Althanstrasse 4, 1090 Vienna, Austria

**Keywords:** Remyelination, EphrinB3, Oligodendrocytes, Multiple sclerosis

## Abstract

Remyelination in multiple sclerosis (MS) lesions often remains incomplete despite the presence of oligodendrocyte progenitor cells (OPCs). Amongst other factors, successful remyelination depends on the phagocytic clearance of myelin debris. However, the proteins in myelin debris that act as potent and selective inhibitors on OPC differentiation and inhibit CNS remyelination remain unknown. Here, we identify the transmembrane signalling protein EphrinB3 as important mediator of this inhibition, using a protein analytical approach in combination with a primary rodent OPC assay. In the presence of EphrinB3, OPCs fail to differentiate. In a rat model of remyelination, infusion of EphrinB3 inhibits remyelination. In contrast, masking EphrinB3 epitopes using antibodies promotes remyelination. Finally, we identify EphrinB3 in MS lesions and demonstrate that MS lesion extracts inhibit OPC differentiation while antibody-mediated masking of EphrinB3 epitopes promotes it. Our findings suggest that EphrinB3 could be a target for therapies aiming at promoting remyelination in demyelinating disease.

**Electronic supplementary material:**

The online version of this article (doi:10.1007/s00401-015-1521-1) contains supplementary material, which is available to authorized users.

## Introduction

Myelin sheaths are layered, lipid-rich structures that wrap around axons to mediate salutatory signal conduction along them and provide them with trophic support [38, 45]. In the central nervous system (CNS), myelin sheaths are formed by oligodendrocytes [9]. Myelin is a target of demyelinating diseases, such as multiple sclerosis (MS) [22, 30]. In response to myelin degeneration, multipotent parenchymal progenitor cells called oligodendrocyte progenitor cells (OPCs) are activated and recruited to the damaged areas [22]. These can regenerate myelin to some extent, but the process is often incomplete [35], leaving axons permanently demyelinated and vulnerable to degeneration [6].

The reason for remyelination failure in MS is still not fully understood, and is expected to be complex. Several studies have demonstrated that a proportion of chronically demyelinated MS lesions contains OPCs that fail to differentiate into remyelinating oligodendrocytes [10, 30, 32, 52]. It is likely that intrinsic changes in OPCs as well as extrinsic inhibitors, such as chondroitin sulphate proteoglycans, that accumulate in MS lesions, contribute to the differentiation failure of OPCs in MS [1, 5, 12, 20, 23, 24, 28–30, 34, 46, 49].

We and others have demonstrated that successful remyelination is dependent on an active inflammatory response [5, 30], and especially the presence of macrophages [29]. The switch from proliferation-inducing M1 macrophages to M2 type activation may promote OPC differentiation [37]. Experimental studies of remyelination indicate that the role of macrophages in remyelination is twofold: first, macrophages contribute by secretion of cytokines and second, by the phagocytic clearance of myelin debris [31, 37]. The elimination of myelin debris is an important prerequisite for remyelination. Studies in primary rodent OPC cultures have demonstrated that unknown proteins in myelin exert potent and selective inhibitory effects on OPC differentiation [2, 41, 48]. Furthermore, supplementation of experimentally induced demyelinated lesions with myelin debris results in a nearby complete suppression of remyelination in vivo without affecting the presence of OPC response [28]. Electron micrographs of biopsies of acute MS lesions [30] and proteomic analysis of MS lesions postmortem indicated that myelin proteins may accumulate and persist in MS lesions [26]. In the present study, we sought to identify the molecular substrate in myelin that is responsible for inhibiting OPC differentiation, and to determine whether neutralization of myelin inhibitors may constitute a novel target for remyelination therapies.

## Materials and methods

### Ethics statement

All animal experiments were conducted in accordance with animal welfare regulations of the German animal protection law, the Niedersächsisches Landesamt fur Verbraucherschutz und Lebensmittelsicherheit (license: RKO_033/2008), Max Planck Institute, UK home office and ARRIVE guidelines.

### Characterization of chronic active MS lesions

Postmortem MS lesion tissue was provided by the UK MS tissue bank. Chronic active lesions were identified by H&E staining and defined by the presence of immune cells (HLA + T-lymphocytes) and the absence of foamy macrophages. Active lesions contained foamy macrophages.

### Immunohistochemistry of MS lesions

Immunohistochemistry on MS lesions was performed as described in [50]. Sections were stained with antibodies to Nkx2.2 (1:150) and β3tubulin (1:500), HLA (1:150), MBP (1:300), GFAP (1:500) degenerated MBP (1:500). Appropriate Alexa 488, 555 or 594-conjugated secondary antibodies (Invitrogen) were used. Cell nuclei were visualized with DAPI (Sigma-Aldrich). A list with details of the antibodies used is included in Supplementary Table 8.

### Laser capture microdissection

MS lesions were visualized on 30 µm cryostat sections by rapid immunohistochemistry with antibodies to MOG (1:20). MOG-negative lesions cores were harvested by laser capture microdissection (P.A.L.M. Microlaser Systems) and proteins were extracted using Lysis buffer (8 M urea, 4 % w/w CHAPS, 5 mM magnesium acetate, 10 mM Tris pH 8.0 and protease inhibitor cocktail set I at 1 × concentration (Calbiochem).

### Preparation of myelin protein extracts (MPE)

Myelin (rat and human) was purified from mechanically dissociated brains by two rounds of discontinuous density gradient centrifugation and osmotic disintegration. Protein extracts were prepared by dissolving myelin pellets with 1 % *N*-octyl-β-d-glucopyranoside, 0.2 M sodium phosphate pH 6.8, 0.1 M Na_2_SO_4_, and 1 mM ethylenediaminetetraacetic acid.

### Enrichment of inhibitory activity in MPE by column chromatography

MPE (50 mg) was filtered, desalted and concentrated (Amicon ultrafiltration cell, Millipore) with 50 mM sodium acetate (pH 4). Column chromatography was performed by FPLC (Pharmacia Fine Chemicals, GE Healthcare). First, MPE was separated by cationic chromatography (Econo-Pac CM cartridges, 1 ml, BioRad). To test the resulting fractions for inhibitory activity, OPCs were exposed to substrates that were prepared using equal volumes (100 µl) of eluates, and the number of O4 + OPCs was determined. Further separation of inhibitory fractions was achieved using anionic chromatography (EconoPac High Q cartridge, BioRad) and gel exclusion chromatography (Sephacryl S100 column, GE Healthcare). Protease inhibitors (Thermo Scientific) were used during all stages.

### Proteomic analysis of MS lesions and MPE fractions

Protein extracts were precipitated with ammonium acetate in methanol, separated by 1D SDS-PAGE, and stained with colloidal Coomassie blue. Each track was cut into 1 mm slices (16 per track). Mass spectrometry experiments were performed using an LTQ linear ion trap instrument fitted with a nanospray ion source (Thermo, San Jose, CA). The LTQ was operated in a data-dependent manner, and ions with a charge state of 2+ or 3+ (indicative of a tryptic peptide) were automatically isolated, fragmented by CID and an MS/MS spectrum generated. The separation of peptides was performed by reverse-phase chromatography using an Agilent 1200 (Agilent Technologies, Santa Clara, CA), a HPLC pump at a flow rate of 300 nL/min and a LC-Packings (Dionex, Sunnyvale, CA) PepMap 100 column (C18, 75 uM i.d. × 150 mm, 3 μM particle size). Peptides were loaded onto a LC-Packings pre-column (Acclaim PepMap 100 C18, 5 μM particle size, 100 A, 300 μM i.d × 5 mm) in 0.1 % formic acid for 5 min at a flow rate of 20 μL/min to desalt samples. A gradient employed of 5–55 % B in 60 min was used to elute peptides (Solvent A = 0.1 % formic acid in water and solvent B = 5 % acetonitrile with 0.1 % formic acid in water). Data were processed using Bioworks Browser (version 3.3.1 SP1, ThermoFisher), and searched using MASCOT (Matrix Science Ltd) using a fixed modification of carbamidomethyl and a variable modification of oxidation (M). The databases used were NCBInr 060629 *Rattus* or *Homo sapiens* with a Peptide Mass Tolerance of ±1 Da and a Fragment Mass Tolerance of ±0.8 Da.

### Preparation of primary OPC cultures

Primary OPC cultures were isolated from neonatal (p0–2) Sprague–Dawley rat forebrains as in [2, 47]. Differentiation was induced with Sato’s medium containing 0.5 % FCS. Only cultures ≥94 % A2B5+ cells were used. OPCs were plated at a density of 2 × 10^4^ cells (8-well chamber slides) or 3 × 10^5^ cells (6-well plates).

### Immunocytochemistry

Immunocytochemistry on OPCs was conducted as in. O4, Nkx2.2, Mbp-positive cells were quantified relative to DAPI-stained nuclei in >20 randomly selected eye fields on an Olympus X80 microscope. The morphology of OPCs stained with phalloidin was categorized as follows: I: mono/bipolar; II: multipolar, primary branches; III: multipolar, secondary branches; IV: membranous processes.

### Preparation of MS lesion, MPE, and EphrinB3 substrates

MS lesion extract (see below), MPE and EphrinB3-Fc (R&D) substrates were prepared by overnight incubation on PLL (Sigma-Aldrich) coated dishes as in [48, 49].

### Pre-clustering of recombinant EphrinB3-Fc

EphrinB3-Fc fragments were mixed with anti-human Fc-IgG (Millipore) (ratio = 1:5) and incubated for 2 h at room temperature prior to addition to the tissue culture medium.

### Effects of EphrinB3 on late-stage OPCs

OPCs were differentiated in Sato’s differentiation medium for 48 h, and subsequently exposed to pre-clustered EphrinB3 suspended in Sato’s medium for another 24 h. Cells were then fixed with 4 % PFA and assessed for O4 and Mbp expression.

### Neutralization of EphrinB3 epitopes in MPE

EphrinB3 epitopes in MPE substrates and MS lesion extracts were neutralized by incubation with anti-EphrinB3 antibodies (Abcam and R&D; ratio: 1:1) in Sato’s differentiation medium for 2 h at room temperature prior to cell seeding.

### Phosphorylation assays

Immunoprecipitations of cell lysates bound with respective antibodies were conducted using Protein A/G agarose beads (Santa Cruz). Following SDS-PAGE separation and Western blotting, membranes were probed with anti-phosphotyrosine antibodies (1:2500). Blots were subsequently stripped, blocked, and re-probed with antibodies binding the respective receptor. Receptor activation is represented by the ratio of relative optical densities (ROD) of phosphorylated vs. total protein.

### RhoA GTPase activity assay

As in [2], RhoA activation was detected using a commercial RhoA assay (Millipore).

### TUNEL assay

To detect cell death, TUNEL assays (Promega) were conducted and the percentage of apoptotic nuclei determined [2].

### Proliferation assay

The OPC were cultured in Sato’s differentiation medium for 24 h. Subsequently, cells were fixed and stained with Olig2 (1:1000) and PCNA (1:500). Number of cells was counted in random field with >200 cells per experiment. A minimum of three biological replicates were conducted.

### Reverse transcriptase-PCR and q-RT PCR

RNA was extracted using RNeasy Mini Kit (Qiagen). Reverse transcription (first-strand cDNA synthesis kit for RT–PCR, Roche Applied Science) and second-round PCR was performed using GoTaq DNA polymerase (Promega). Primers used are summarized in Supplementary Table 7. q-RT-PCR was conducted on an Applied Biosystems 7500HT Fast Real-time PCR system [2, 49]. Triplicate measurements were made on a minimum of three biological replicates.

### siRNA-mediated gene-silencing

siRNA transfections of purified OPCs were conducted with lipofectamine RNAiMAX transfection reagent (Invitrogen) in OPTI-MEM as in [2]. The knockdown efficiency was established by qPCR.

### Animal experiments

#### Immunogold electron microscopy

Perfusion-fixed (4 % formaldehyde, 0.2 % glutaraldehyde) corpus callosum white matter was cryoprotected (2.3 M sucrose), mounted onto aluminium pins, and frozen in liquid nitrogen. Ultrathin cryosections (Leica UC6 cryo-ultramicrotome) were collected (2 % methylcellulose:2.3 M sucrose = 1:1), blocked (1 % BSA), and incubated with antibodies to EphrinB3 (1:200; R&D). Gold-conjugated secondary antibodies (Aurion) were used to visualize EphrinB3 epitopes on a LEO EM912 Omega transmission electron microscope (Zeiss) fitted with an on-axis 2048 × 2048 CCD camera (Proscan).

#### EphrinB3 knockout mice

EphrinB3 knockout mice were kindly provided by Amparo Acker-Palmer and genotyped as previously [43].

#### Induction of focal demyelination

Female Sprague–Dawley rats were anesthetized with ketamine (75 mg/kg) and xylazine (10 mg/kg) and positioned in a stereotactic frame. Demyelination was induced bilaterally by stereotactic injection of ethidium bromide (0.01 %, 4 µl) into the CCP-young animals (10.4 mm caudal, 2.6 mm lateral and 7.07 mm ventral to bregma) and older animals (11.1 mm caudal, 2.8 mm lateral and 7.8 mm ventral to bregma).

#### Infusion of EphrinB3-Fc-IgG and anti-EphrinB3 antibodies into demyelinated lesions

EphrinB3 and anti-EphrinB3 antibodies were administered via osmotic pumps (ALZA Corporation) at 10 and 3 dpi, respectively (200 μg/ml in PBS). EphrinB3-Fc (R&D Systems) was pre-clustered with anti-human-Fc (Chemicon) for 2 h at room temperature (EphrinB3-Fc:anti-Fc = 10:1) prior to pump filling. Control animals received anti-Fc IgG only. Anti-EphrinB3 antibodies (R&D:Abcam ratio = 1:1). Controls received Human IgG. Animals were perfused at 28 and 14 dpi, respectively.

#### Immunohistochemistry of CCP lesions

Rats were anesthetized and transcardially perfused with 4 % PFA. The brains post-fixed, cryoprotected (30 % sucrose) and snap frozen. 15 µm cryostat sections were stained with antibodies to EphA4 (1:500), Nkx2.2 (1:150), β3tubulin (1:500).

#### In situ hybridization

In situ hybridization was conducted on cryostat sections using digoxigenin-labelled complementary RNA probes for *Msrb*, and *Pdgfr*-*α* as in [28, 49]. Lesions were identified on digital images of solochrome-cyanine-stained sections, and the lesion area was determined using Image J 1.43b (http://rsb.info.nih.gov/ij/). ISH-stained cells in the lesions were counted on digitized adjacent sections. To rule out effects attributable to the size of the lesion, only lesions <0.4 mm^2^ were included for analysis [49].

#### Histological analysis of remyelination

Remyelination was assessed on tissue fixed with 4 % glutaraldehyde, osmicated and processed into resin (TAAB Laboratories) [28]. Sections (1 µm) were stained with methylene blue and Azur-II. Rank analysis was conducted as in [49]. Lesions with the greatest extent of remyelination were assigned the highest rank value. In addition, remyelinated and demyelinated axons were manually counted on a minimum of 3 digitized 100x images of resin-embedded lesions.

#### Electron microscopy

Ultrathin sections (50 nm, Leica Ultracut S ultramicrotome) were stained with aqueous 4 % uranyl acetate and lead citrate. Electron micrographs were obtained as outlined above [28, 49].

### Statistical analysis/experimental design

All analyses were conducted with researcher blind to treatment group. Experimental animals were randomized to treatment group. Data were analysed using GraphPad software (Prism). Multiple group comparisons were conducted using one-way ANOVA followed by Dunnett’s post test. Two-tailed Student’s *t* test was used to test receptor activation, qPCR data. Cellular responses in vivo were analysed using Student’s *t* test. For rank analysis, a two-tailed Mann–Whitney *U* test was used.

## Results

### Human myelin extracts inhibit OPC differentiation

**Fig. 1:**
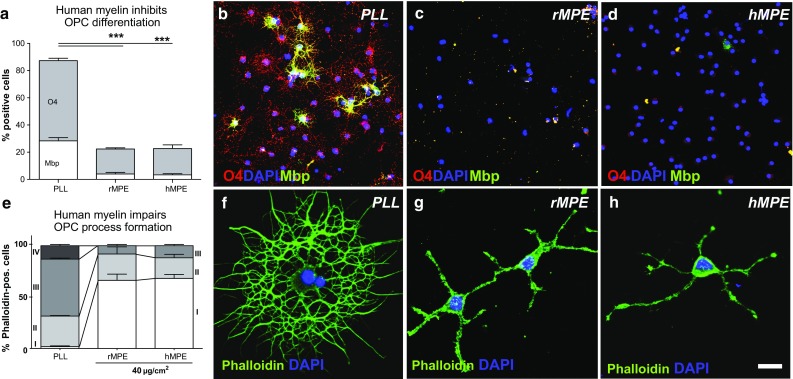
Human myelin extracts contain selective inhibitors of OPC differentiation. **a**–**d** OPCs exposed to extracts of rat myelin extracts (rMPE) and human myelin preparations (hMPE) respond by down-regulation of O4 and Mbp expression (2-day differentiation, *n* = 3; ANOVA: O4 ***P < 0.0001, Mbp ***P < 0.0001; Dunnett’s post hoc test PLL vs. rMPE: *P* < 0.0001; PLL vs. hMPE *P* < 0.0001). **e**–**h** The presence of hMPE also induced a reduction of the complexity of OPC processes and an increase of earlier morphological stages compared with OPCs plated on control substrate (2-day differentiation, *n* = 3; ANOVA: *P* < 0.0001; *I* mono/bipolar, *II* primary, *III* secondary, *IV* membranous branches). *Error bars* ± SEM. *Scale bar* in **b**–**d** and **f**–**h** = 30 μm

Inhibitory effects on OPC differentiation have been described for rodent [2, 41, 48] and porcine myelin [48]. We therefore first sought to establish that the OPC-differentiation-inhibitory activity is also found in human myelin protein extracts (Fig. 1a–d). Assessment of OPCs cultured for 48 h on PLL control, rat and human myelin substrates confirmed strongly inhibited expression of the early differentiation marker O4 and myelin basic protein (MBP), a protein expressed by mature oligodendrocytes. Furthermore, human myelin extracts also profoundly inhibited process formation in OPCs (Fig. 1e–h). The effects of human myelin on OPC differentiation were specific, as TUNEL assays and cell counts did not reveal any differences from control substrates (Supplementary Fig. 1).

### Identification of EphrinB3 as a myelin-associated inhibitor of OPC differentiation

**Fig. 2:**
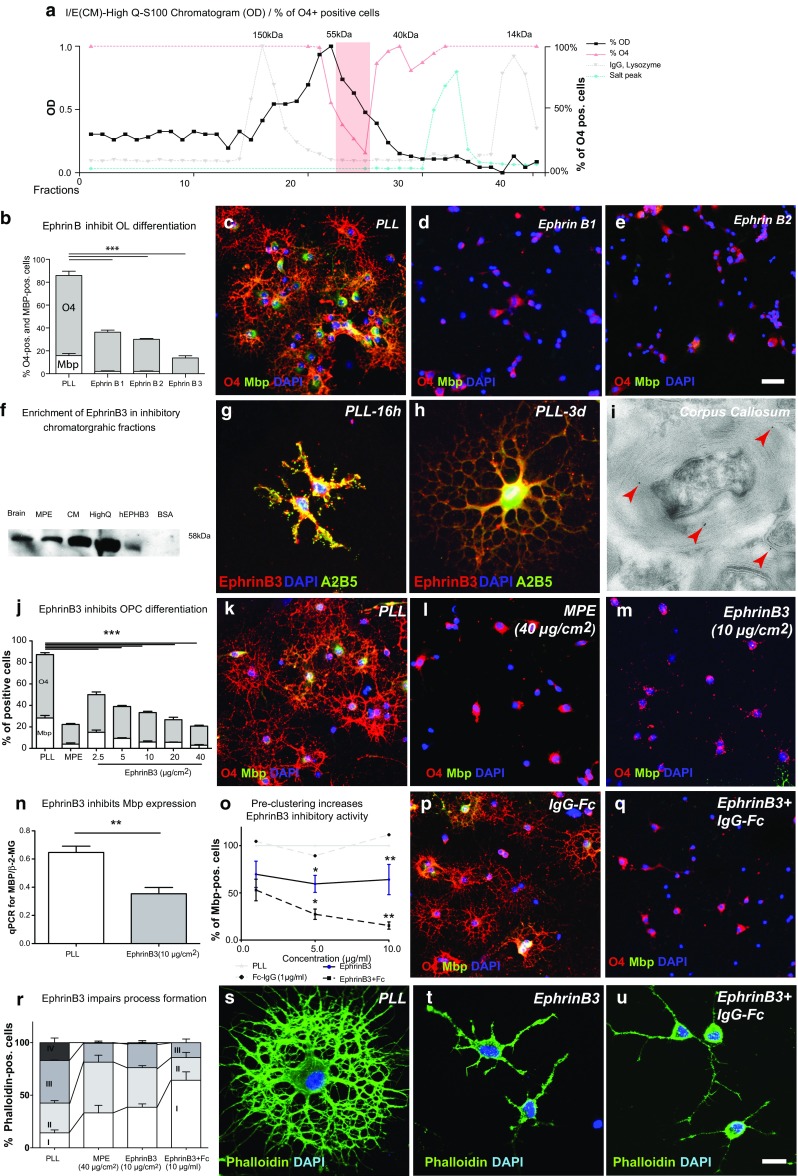
EphrinB3 is associated with the inhibitory activity of myelin extracts and inhibits OPC differentiation in vitro. **a** Myelin protein extracts were separated into inhibitory fractions (*red line* percentage of O4^+^ cells cultured in the presence of individual fractions) using CM → HiqhQ → Sephadex S100 size-exclusion chromatography (*black line* S100 chromatogram, *grey line* IgG and lysozyme peak). Fractions containing proteins with a calculated weight of 51 kDal were associated with the highest inhibitory activity. **b** *Bar graph* showing the decrease of O4+ and Mbp+ oligodendrocyte lineage (OL) cells cultured in differentiation medium in the presence of recombinant human EphrinB1, B2, B3 (2-day differentiation, *n* = 3; ANOVA: *O4* ***P < 0.0001, *Mbp* ***P < 0.0001; Dunnett’s post hoc test: PLL vs. EphrinB1 or EphrinB2 or EphrinB3 at 10 μg/cm^2^: *P* < 0.0001). **c**–**e** Representative images of cells immunolabelled for O4 (*red*) and MBP (*green*) cultured on control substrates (PLL) and EphrinB1, B2 substrates. **f** Immunoblot demonstrating that CM and subsequent HighQ column chromatography-based enrichment of OPC-differentiation-inhibiting activity results in concordant enrichment of EphrinB3 (*brain* total brain lysates, *BSA* bovine serum albumin). **g**, **h** EphrinB3 is expressed in immature OPCs (16 h differentiation), throughout to mature oligodendrocyte stages (3-day differentiation). **i** Electron microscopy immunogold labelled cryo-ultramicrotome sections of murine corpus callosum white matter at 1 months of age demonstrated the presence of EphrinB3 (*red arrow*) in myelin sheaths in vivo. **j**–**m** EphrinB3 inhibited OPC differentiation in a dose-dependent manner (2-day differentiation, *n* = 3; ANOVA: O4 ***P < 0.0001, Mbp ***P < 0.0001; Dunnett’s post hoc test: PLL vs. MPE or vs. EphrinB3 at 2.5, 5, 10, 20 or 40 μg/cm^2^: *P* < 0.0001. **n** RT-qPCR confirmed reduced expression of *Mbp* mRNA in the presence of EphrinB3 (*n* = 3; *t* test; **P < 0.001). **o**–**q** When OPCs were exposed to recombinant EphrinB3-Fc pre-clustered with anti-Fc antibodies mimicking surface-bound EphrinB3, the inhibitory activity was increased (2-day differentiation; *n* = 3; ANOVA: Mbp ***P < 0.0001; Dunnett’s post hoc test: EphrinB3 vs. EphrinB3 + IgG-Fc at 1, 5 or 10 µg/ml): *P* < 0.0001. **r**–**u** EphrinB3 also induced a reduction in the complexity of OPC processes and a corresponding increase of earlier morphological stages (2-day differentiation; *I* mono/bipolar, *II* primary, *III* secondary, *IV* membranous branches) (*n* = 3; ANOVA: *P* < 0.0001). *Error bars* ± SEM. *Scale bar* in **c**–**e**, **k**–**m**, **p**–**q** = 30 μm, in **i** = 2 μm, in **s**–**u** = 20 μm

We previously demonstrated that the inhibitory activity of myelin is associated with the protein and not the lipid fraction [48]. To identify the protein responsible for the inhibition, we developed a protocol for biochemical separation of protein fractions and enrichment of inhibitory activity. This fractionation included three steps: 1) enriching inhibitory activity by carboxymethyl (CM), 2) High Q column ion exchange chromatography, and 3) Sephadex S100 mediated size exclusion (Supplementary Fig. 2). LC–MS/MS analysis of the inhibitory fractions followed by testing of candidate proteins with primary OPCs was not able to identify an inhibitory protein (Supplementary Table 1). Potential reasons why MS/MS identification was unsuccessful include that the protein responsible for the inhibition might be either in low abundance or hydrophobic. As an alternative approach, we sought to estimate the size of the inhibitory protein, which would then facilitate a bioinformatic search. For this purpose, fractions obtained following CM and High Q separation were spiked with two proteins of known molecular weight [immunoglobulin G (IgG), 150 kDa, and lysozyme, 14 kDa] before they were further separated on S100 Sephadex size-exclusion columns. The addition IgG and lysozyme to protein fractions did not change the distribution of the inhibitory activity. Based on the localization of the inhibitory activity relative to the reference proteins, the size of the predicted protein was calculated to be approximately 51 kDa (Fig. 2a, Supplementary Fig. 2). Taking into account that post-translational modifications may alter the size of the protein core, we conducted PubMed Protein Database searches for signalling proteins expressed in the CNS that fall within a size range of 30–55 kDa (search terms: “030000:055000 [Molecular Weight] and membrane and signalling and ligand and brain”). From these searches, EphrinB1-B3, a group of transmembrane proteins forming part of the Ephrin family, emerged as candidate proteins. We tested their effects on OPC differentiation by exposing cells to substrates prepared from EphrinB1-3. Amongst these, EphrinB3 had the most potent inhibitory effect on differentiation of oligodendrocyte linage cells (Fig. 2b). Immunoprecipitation of EphrinB3 in CNS, myelin extracts and column chromatography fractions demonstrated enrichment of EphrinB3 (and B1) in parallel with enrichment for OPC-differentiation-inhibiting activity (Fig. 2f). The enrichment and the potent inhibitory effects observed implies an important role for EphrinB3. Nevertheless, other molecules may also contribute to the myelin-associated inhibition of OPC differentiation.

### EphrinB3 inhibits OPC differentiation

Class B Ephrins can act both as ligands for Eph-receptor tyrosine kinases (RTKs) (forward signalling) and as receptors for Eph-receptor binding (reverse signalling). Biological functions of Ephrin signalling in the CNS include regulation of axon outgrowth and migration of tumour and stem cells [4, 14, 15]. Expression of EphrinB3 has been noted in the developing CNS, where it has an important role preventing the crossing of corticospinal tract axons in the spinal cord and ensuring contralateral innervation of alpha-motor neurons located in the ventral horns [17]. In the adult CNS, EphrinB3 is mainly expressed in white matter (http://www.brainatlas.org), where it can be detected in oligodendrocytes [4]. We first confirmed the expression of EphrinB3 in cultured oligodendrocyte lineage cells by immunocytochemistry (Fig. 2g, h). Immunogold electron microscopy of murine corpus callosum white matter at 1 months of age suggested that EphrinB3 is located within myelin sheaths in the intact rodent CNS (Fig. 2i).

When OPCs were exposed to substrates containing recombinant EphrinB3-Fc fragments, in which the transmembrane segment was replaced with an Fc tag to make it soluble, a concentration-dependent impairment of O4 and MBP expression was detected (Fig. 2j–m). Furthermore, EphrinB3-Fc also negatively affected process formation and resulted in the majority of OPCs being arrested at an immature monopolar/bipolar stage (Fig. 2r–t). The effects of EphrinB3 on OPC differentiation were specific, as TUNEL assays and cell counts did not reveal any differences from control substrates (Supplementary Fig. 1).

### Clustering EphrinB3 epitopes increases the inhibition of OPC differentiation

EphrinB-EphR signalling occurs by direct cell–cell interactions and results in oligomerisation of receptor-ligand complexes. A particular feature of Ephrin signalling is that clustering of Ephrin-Eph receptors can modulate the signalling process. In most cases, ligand-receptor clustering leads to enhanced signalling [16]. To mimic interaction with membrane-bound EphrinB3, we exposed OPCs to aggregated EphrinB3-Fc using anti-Fc-IgG. Significantly fewer cells differentiated when exposed to pre-clustered EphrinB3 than non-clustered EphrinB3 (Fig. 2o–k).

### EphrinB3 signals via EphA4 receptor and activates differentiation-inhibiting intracellular cascades in OPCs

**Fig. 3:**
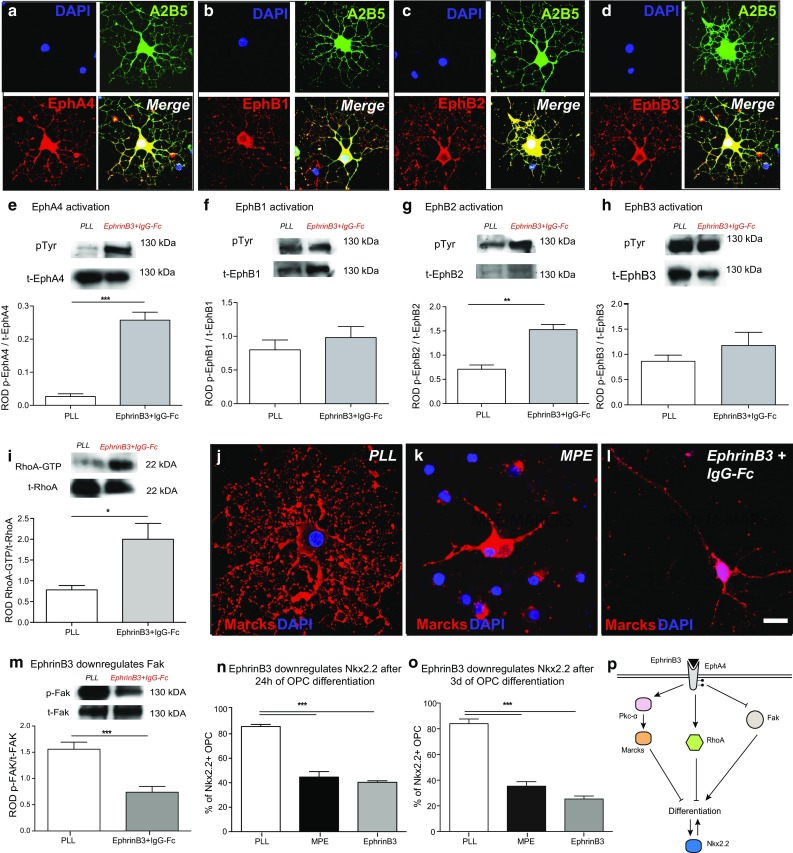
EphrinB3 activates RhoA and Pkc-α, inhibits Fak, and decreases Nkx2.2 expression. **a**–**d** OPCs in culture for 48 h expressed EphA4, EphB1, EphB2 and EphB3 receptor tyrosine kinases (RTKs). **e**–**h** EphrinB3-induced phosphorylation of EphA4 and EphB2 RTKs in OPCs [immunoprecipitation followed by WB, *pTyr* phosphorylated Eph-RTK, *t*-*Eph* total Eph-RTK, quantification of phosphorylated Eph-RTK relative to total Eph-RTK (ROD), *n* = 3: *t* test; EphA4 ***P < 0.05; EphB1 *P* > 0.1; EphB2 **P < 0.01; EphB3 *P* > 0.1]. **i** Furthermore, EphrinB3-induced RhoA activity in OPCs (24-h differentiation; quantification of RhoA-GTP relative to RhoA-GDP (ROD), *n* = 3: ANOVA; *P < 0.05). **j**–**l** Whilst Marcks is localized at the membrane in differentiating OPCs cultured on control substrates, EphrinB3-mediated activation of Pkc-α resulted in phosphorylation and membrane-to-cytosol translocation of Marcks. **m** EphrinB3 also inhibited the activation of Fak [quantification of phosphorylated (p-)Fak relative to total (t-)Fak: (ROD), *n* = 3; *t* test; **P < 0.001]. **n**, **o** Similar to MPE, EphrinB3 inhibited Nkx2.2 expression in OPCs (24-h differentiation, *n* = 3; ANOVA; ****P < 0.0001; Dunnett’s post hoc test PLL vs. MPE: *P* < 0.001; PLL vs. EphrinB3: *P* < 0.001; 3-day differentiation, *n* = 3; ANOVA; ****P < 0.0001; Dunnett’s post hoc test PLL vs. MPE: *P* < 0.001; PLL vs. EphrinB3: *P* < 0.001). **p** Proposed model of EphrinB3 signalling regulating OPC differentiation: the presence of EphrinB3 activates EphA4 and EphB2-RTKs. This results in activation of RhoA and Pkc-α signalling, both known to inhibit OPC differentiation. EphrinB3 also inhibits Fak signalling, a positive regulator of OPC differentiation. Failed differentiation is associated with reduced expression of Nkx2.2. *Error bars* ± SEM. *Scale bar* in **a**–**d** = 40 µm, **j**–**l** = 20 μm

The B class of transmembrane Ephrins preferentially binds to EphB receptors. In addition, the EphA4 receptor can also recognize EphrinB3. We found that OPCs express EphB1-3 and EphA4 receptors at both the transcriptional and the protein levels (Fig. 3a–d, Supplementary Fig. 3). To investigate which of these receptors respond to the addition of recombinant EphrinB3-Fc to Sato’s differentiation medium, we conducted immunoprecipitation of OPC protein extracts with antibodies against individual Eph receptors followed by Western blotting with anti-phosphotyrosine antibodies. This revealed a strong activation of EphA4 and EphB2 receptors in response to EphrinB3-Fc (Fig. 3e–h). However, EphB2 seemed to be expressed at relatively low levels in OPCs when compared to EphA4 expression (Supplementary Fig. 3).

Ephrin-Eph signalling has been associated with modulation of Rho-GTPases. Myelin-associated inhibitors also activate RhoA signalling and inhibition of RhoA can neutralize inhibitory effects of myelin [2]. We therefore hypothesized that RhoA signalling might be activated in OPCs in response to EphrinB3. RhoA activity assays on OPCs cultured in the presence and absence of EphrinB3 confirmed activation of RhoA in OPCs exposed to Ephrin B3 (Fig. 3i). In the past, we also demonstrated that myelin protein extracts activate protein kinase C-α (Pkc-α) to mediate inhibitory effects on OPC differentiation, and that inhibition of Pkc-α promotes OPC differentiation in the presence of myelin [2]. Inhibitory PKC-α-mediated signalling is associated with phosphorylation and membrane-to-cytosol translocation of Myristoylated alanine-rich C-kinase substrate (Marcks) and causes disorganization of the cytoskeleton and redistribution of actin filaments [2, 3]. We assessed the subcellular location of Marcks in OPCs and found that in the presence of EphrinB3 or myelin protein extract it was trapped in the cytosol, whereas on control substrate it was located at the membrane. This demonstrated that EphrinB3 induces Pkc-α activity (Fig. 3j-l). Taken together, our findings indicate that EphrinB3 is sufficient to explain activation of the inhibitory cascades in OPCs that have been associated with myelin extracts. In addition, we found that EphrinB3 also inhibited the activity of Focal adhesion kinase (Fak) (Fig. 3m), a positive regulator of early OPC differentiation [21] that has been implicated in Ephrin-EphA4 signalling [8].

To assess transcriptional consequences of Ephrin signalling in OPCs, we investigated the expression of Nkx2.2, a functionally important transcription factor that is known to be up-regulated at the onset of OPC differentiation [19], but whose expression fails to increase in the presence of myelin extracts in vitro [48] and in vivo [28]. We found reduced expression of Nkx2.2 in the presence of EphrinB3 (Fig. 3n,o).

### Assessment of the effects of EphrinB3 on CNS remyelination in vivo

Having established an important regulatory function of EphrinB3 on OPC differentiation in vitro, we assessed the effects of EphrinB3 on myelin regeneration in vivo. For this purpose, we used a well-characterized model of remyelination, in which a focal demyelination lesion is induced by stereotactic injection of ethidium bromide into the rat caudal cerebellar peduncle (CCP) (Supplementary Fig. 4 a,b). This results in rapid demyelination and recruitment of new OPCs. Around day 10 post lesion induction, the recruited OPCs start to differentiate into myelin-forming oligodendrocytes. 28 days post lesion induction, the lesions are fully remyelinated [28, 53].

### OPCs express EphA4 receptor in models of CNS remyelination and human MS lesions

**Fig. 4:**
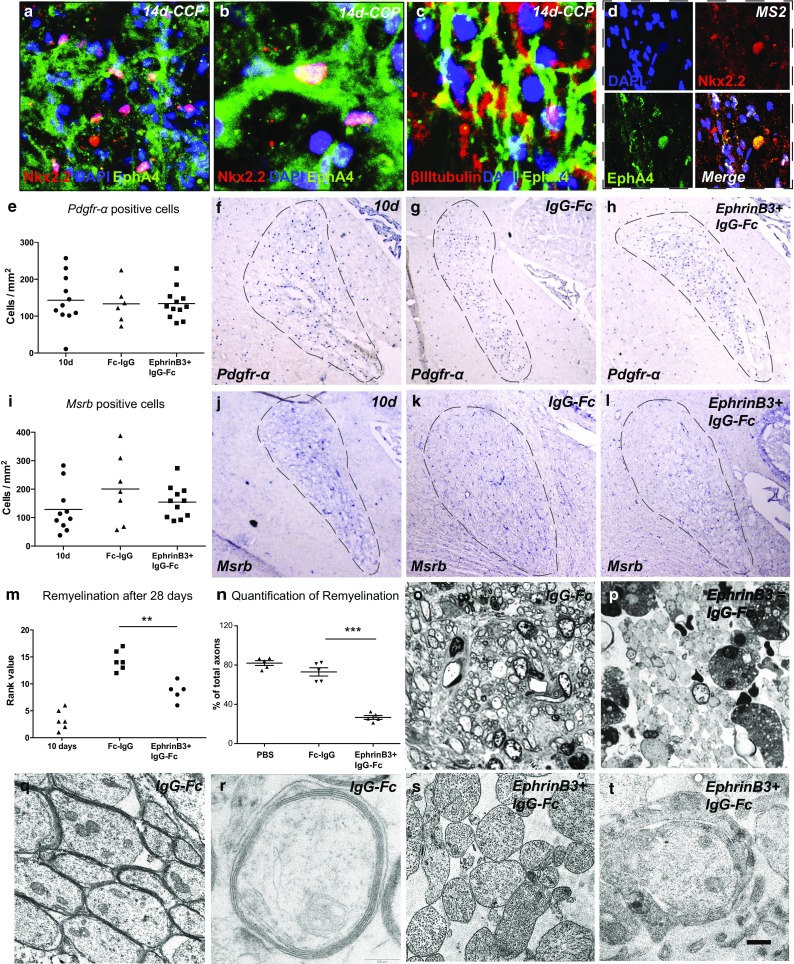
The presence of EphrinB3 inhibits CNS remyelination. **a**–**d** EphA4 receptor is expressed by Nkx2.2^+^ OPCs and βIII-tubulin^+^ axons in experimental CCP lesions, as well as **d** in chronic active human white matter MS lesions. **e**–**h** In situ hybridization for *Pdgfr*-*α* showed comparable OPC densities within lesions of EphrinB3-treated animals and controls 28 days after lesion induction (dpi) (10 dpi—prior to EphrinB3 infusion: *n* = 11; IgG-Fc: *n* = 6; EphrinB3 + IgG-Fc: *n* = 12; ANOVA: *P* > 0.1). **i**–**l** Similarly, levels of *Msrb* ^+^ macrophages remained unaffected (10 dpi: *n* = 9; IgG-Fc: *n* = 7; EphrinB3 + IgG-Fc: *n* = 11; ANOVA: *P* > 0.1). **m**–**t** The extent of remyelination was assessed on methylene blue and Azur-II-stained semithin sections. EphrinB3 infusion induced a significant impairment of CNS remyelination as compared to controls (IgG-Fc control *n* = 6; EphrinB3 *n* = 5; Mann–Whitney *U* test; **P < 0.001). **n** Quantification of demyelinated and demyelinated axons confirms that the presence of EphrinB3 impairs CNS remyelination (PBS control *n* = 5, IgG-Fc control *n* = 6; EphrinB3 *n* = 5; *t* test; ***P < 0.0001). **o** Remyelination in controls was nearly complete at 28 dpi; however, **p** in EphrinB3-infused animals, the majority of axons remained demyelinated. **q**, **r** EM analysis demonstrated successful formation of compact myelin sheaths in controls, whereas **s**, **t** in EphrinB3-infused lesions axons remained demyelinated. **t** Occasionally, axons were contacted by oligodendroglial processes in EphrinB3-treated animals that failed to form compact myelin. *Error bars* ± SEM. *Scale bar* in **a** = 30 μm; in **b**, **c** = 50 μm; in **d** = 40 μm; in **f**–**h**, **j**–**l** = 200 μm; in **o**, **p** = 100 μm; in **q**, **r** = 2 μm; in **s**, **t** = 0.5 μm

We first investigated whether OPCs can respond to EphrinB3 in demyelinated lesions by assessing the expression of EphA4 receptor in CCP lesions (Fig. 4a–c). A large number of Nkx2.2^+^ OPCs were immunoreactive for EphA4. However, as expected, EphA4 expression was not restricted to OPCs. To confirm expression of EphA4 in the context of human MS, we conducted immunohistochemical stainings on postmortem CNS white matter tissue containing chronic active MS lesions. This confirmed that Nkx2.2^+^ OPCs express EphA4 receptors in MS lesions (Fig. 4d).

### EphrinB3 infusion does not affect the recruitment of OPCs

To assess the effects of EphrinB3 on remyelination, EphrinB3-Fc pre-clustered with anti IgG-Fc (to mimic the surface-bound presentation of EphrinB3 on myelin debris) was stereotactically infused into the CCP, starting at 10 days post lesion induction (dpi), and compared with controls infused with IgG-Fc (and vehicle-PBS only) (Fig. 4n). Successful delivery of recombinant EphrinB3-Fc was confirmed by immunohistochemistry (Supplementary Fig. 4e). Quantification of OPCs by in situ hybridisation using probes to platelet-derived growth factor α-positive (Pdgfr-α^+^) did not reveal any differences between EphrinB3-exposed and control animals (Fig. 4e–h).

### EphrinB3 does not alter the presence of macrophages

Because the innate immune response plays an important role in remyelination [29], we also assessed the number of macrophages positive for Macrophage scavenger receptor B (Msrb). We found that infusion of pre-clustered EphrinB3-Fc did not induce any changes in the number of macrophages (Fig. 4i–l).

### EphrinB3 inhibits CNS remyelination

The extent of remyelination at 28 dpi was assessed on semithin resin sections (Fig. 4m–p). As expected at this time point, remyelination in control animals was almost complete [53] with the majority of axons bearing thin myelin sheaths that are characteristic of remyelination [7] (Fig. 4o). In contrast, in lesions infused with EphrinB3-Fc, the majority of axons remained demyelinated (Fig. 4p). A significant difference was found between the groups by investigator-blinded rank analysis [28, 49] (Fig. 4m) and quantification of remyelinated and demyelinated axons (Fig. 4n, Supplementary Fig. 5a). The density of axons in the lesions was comparable (Supplementary Fig. 5SB). Stereotactic administration of pre-clustered EphrinB3-Fc into intact CCP white matter did not affect the integrity of mature myelin sheaths, as we could not detect any changes in naive (non-lesioned) CCPs following infusion (Supplementary Fig. 5Sd).

### The absence of EphrinB3 accelerates developmental myelination

**Fig. 5:**
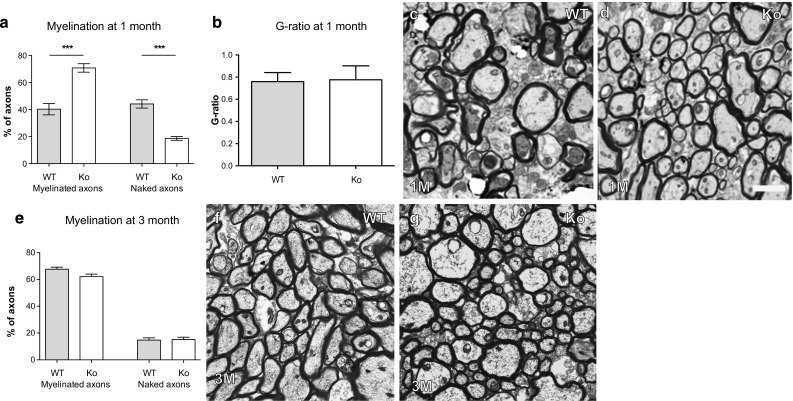
Accelerates developmental myelination in EphrinB3 knockout mice. **a**–**d** 30 days post partum significantly more axons in the corpus callosum of EphrinB3^−/−^ mice were myelinated as compared to WT animals. This was accompanied by a corresponding decrease in the number of nude axons. The relative thickness of the myelin sheaths (*G* ratios) remained unchanged (WT: *n* = 9; EphrinB3^−/−^: *n* = 9; ANOVA: ***P < 0.0001). **e**–**g** After 3 months, the number of the myelinated axons in the corpus callosum in wild-type and EphrinB3^−/−^ mice were comparable (WT: *n* = 5; EphrinB3^−/−^: *n* = 5; ANOVA: *P* > 0.1). *Error bars* ± SEM. Scale **bar** in **c**, **d**, **g**, **h** = 3 μm

The results so far demonstrate that EphrinB3 and its receptor EphA4 are expressed throughout the oligodendrocyte lineage and that the presence of extrinsic EphrinB3 mediates inhibitory effects on the differentiation of OPCs. We next analysed developmental myelination in EphrinB3-deficient mice and found more myelinated axons in the corpus callosum at 1 month in EphrinB3-deficient mice than wild-type mice (Fig. 5e–h). The relative thickness of myelin sheaths as measured by G ratios was the same in wild-type and knockout mice (Fig. 5a–d). At 3 months, the number of myelinated axons was comparable amongst the groups (Fig. 5e–g), indicating that loss or EphrinB3 results in accelerated myelination without affecting myelin thickness or the final extent of myelination.

### Antibody-mediated masking of EphrinB3 epitopes neutralizes the inhibitory effects of myelin extracts on OPC differentiation

**Fig. 6:**
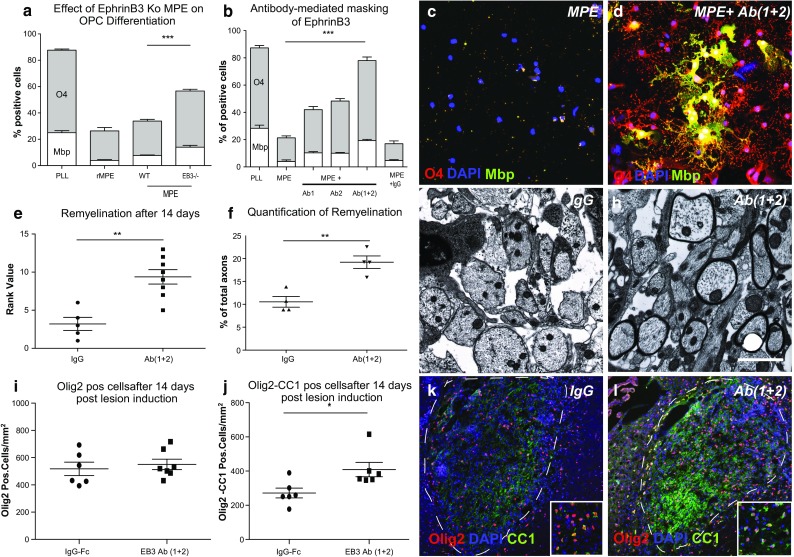
Loss of EphrinB3 and antibody-mediated masking of EphrinB3 epitopes neutralizes the inhibitory effects of myelin extracts on OPC differentiation and promotes CNS remyelination. **a** *Bar graph* demonstrating a significant increase of O4/Mbp-positive OL cells when they were differentiated in presence of MPE from EphrinB3 knock out mice in comparison to MPE from wild-type litter mates. MPE from rat (rMPE) served as internal control (2-day differentiation, *n* = 3; ANOVA: O4 ***P < 0.0001, Mbp ***P < 0.0001; Dunnett’s post hoc test WT-MPE vs. EB3^−/−^-MPE: *P* < 0.0001). **b**–**d** Neutralizing EphrinB3 epitopes using EphrinB3-specific antibodies (Ab1, Ab2) restored the ability of OPCs to differentiate in the presence of inhibitory MPE substrates, whereas the use of unspecific antibodies (IgG) had no effect (*n* = 3; ANOVA: O4 ***P < 0.0001, Mbp ***P < 0.0001; Dunnett’s post hoc test; MPE vs. MPE + Ab1 and Ab2: *P* < 0.0001; MPE vs. MPE + IgG; *P* > 0.1). **e** Quantitative analysis of Olig2-immunohistochemical staining cells showed comparable levels of total oligodendrocyte lineage cells in control and EphrinB3 antibodies infused lesions. **f** In contrast, EphrinB3-antibody-treated animals displayed increased OPC differentiation as the number of Olig2+/CC1+ mature oligodendrocytes was increased (control-IgG: *n* = 6, EphrinB3-Ab(1 + 2): *n* = 6, Student *t* test *P* > 0.05; Olig2-CC1). Representative images from control **k** and EphrinB3 antibody infused lesions **l** at 14 days post lesion induction stained for Olig2 and CC1. **i** Infusion of EphrinB3-specific antibodies (Ab1 + 2) into CCP lesions also accelerated CNS remyelination as evident from the rank analysis 14 dpi [control-IgG: *n* = 8, EphrinB3-Ab(1 + 2): *n* = 5, Mann–Whitney *U* test **P < 0.01]. **j** Manual quantification of remyelinated and demyelinated axons within lesions confirmed accelerated remyelination in Anti-EphrinB3-treated animals [control-IgG: *n* = 4, EphrinB3-Ab(1 + 2): *n* = 4, *t* test **P < 0.01]. **k**, **l** Electron micrographs demonstrating enhanced remyelination in EphrinB3-Ab(1 + 2) infused lesions as compared to IgG controls. *Error bars* ± SEM. *Scale bar* in **c**, **d** = 30 μm; in **g**, **h** = 200 μm (in *inset* = 50 μm), in **k**, **l** = 5 μm

To investigate the role of EphrinB3 with respect to the OPC-differentiation-inhibiting effects of myelin, we exposed OPCs to myelin protein extracts from EphrinB3 Ko mice and wild-type littermates (Fig. 6a). Myelin protein extract (MPE) from EphrinB3-deficient mice was significantly less inhibitory than wild-type MPE.

To determine whether antibody-mediated masking of EphrinB3 epitopes could represent a viable therapeutic approach, myelin protein extracts were treated with two different commercially available antibodies directed against two distinct peptide sequences of EphrinB3 (Fig. 6h). Both antibodies induced O4 and MBP expression of OPCs that were plated on the antibody-bound substrates (Fig. 6a–d). A combination of the two antibodies resulted in neutralization of the inhibitory effects of myelin, restoring OPC differentiation to levels observed in cells plated on PLL control substrate.

### Antibody-mediated neutralization of EphrinB3 promotes CNS remyelination

Focal areas of demyelination were induced using ethidium bromide in the CCPs of 9 to 12-month-old female Sprague–Dawley rats. Owing to their greater age, remyelination was expected to be less efficient and delayed. Aged animals serve as a clinically relevant model for remyelination in young to middle-aged adults [25, 42]. Stereotactic infusion of anti-EphrinB3 antibodies was initiated at 3 dpi. Remyelination was assessed on semithin resin-embedded sections at 14 dpi (Fig. 6e–h). As expected, remyelination in control animals that received infusions of control-IgG, or PBS alone was limited. In contrast, widespread remyelination was detected in animals that received anti-EphrinB3 antibodies. The increase in remyelination was confirmed by both, histological rank analysis and manual counts of axons within the lesions. Assessment of the lesions by electron microscopy (Fig. 6h) confirmed the presence of thin myelin sheaths typical for remyelination throughout the lesions in anti-EphrinB3-treated animals. Axons in control lesions remained largely demyelinated (Supplementary Fig. 5 g), but the relative density of axons between the groups was comparable (Supplementary Fig. 5d). Our data demonstrate that the administration of EphrinB3 antibodies did not alter the number of OPCs but instead enabled efficient differentiation (Fig. 6i–j).

### MS lesions contain EphrinB3

Because EphrinB3 in the adult CNS is predominantly expressed by oligodendrocytes, the main source of EphrinB3 present in MS white matter lesions is likely to be myelin debris that accumulates as a consequence of demyelination. Experimental findings demonstrated that the clearance of myelin debris is mediated by macrophages [28]. In MS, the presence of foamy macrophages defines the acute lesion stage. As time passes, macrophages disappear from the lesions whilst other inflammatory cells continue to persist. This demarcates the subacute, or chronic active lesion stage. Chronic (silent) lesions are defined by the complete absence of immune cells [30]. Investigating the presence of myelin proteins including EphrinB3 in subacute (chronic active) lesions therefore would be useful to determine (1) the efficiency of macrophage-mediated clearance of myelin debris, and (2) whether myelin-associated inhibitors are present for prolonged time periods at biologically relevant levels.

To address these questions, we prepared protein extracts from laser capture-microdissected acute (active) and subacute (chronic active) MS lesions. The lesions were visualized on fresh-frozen postmortem tissue using a rapid immunohistochemistry protocol for myelin oligodendrocyte glycoprotein (MOG) (Supplementary Fig. 6b). During the dissection, care was taken to avoid the MOG-positive normal-appearing white matter.

**Fig. 7:**
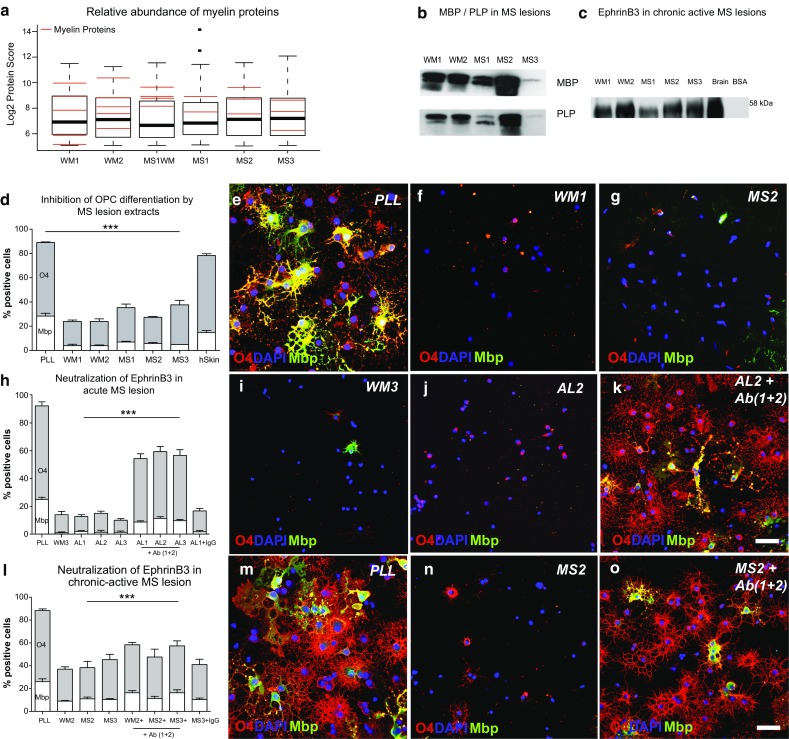
EphrinB3 is present in MS lesions and its neutralization promotes OPC differentiation. **a** Proteins in laser-microdissected tissue of MS lesions were identified using mass spectrometry. The relative abundance of individual myelin-associated proteins (*red bars*) detected in peri-lesional white matter (WM1, 2), normal-appearing white matter (MS1WM), and chronic active MS lesions (MS1-3) was comparable. This indicates that the phagocytosis of myelin debris in MS lesions remained incomplete. **b** Western blots confirmed the presence of myelin proteins (MBP, PLP) in chronic active MS lesion extracts. **c** EphrinB3 was detected in human white matter (WM1, 2) and chronic active MS lesion extracts (MS1-3) by immunoprecipitation and Western blotting. **d**–**g** OPCs cultured on extracts of chronic active MS lesions (MS1–3) and human white matter (WM1, WM2) show reduced O4 + and Mbp + expression compared to OPCs plated on control substrates (poly-l-lysine, PLL) and human skin extracts (hSkin) (2-day differentiation, *n* = 3; ANOVA: O4 ***P < 0.0001, Mbp ***P < 0.0001; Dunnett’s post hoc test: PLL vs. WM and MS extracts: *P* < 0.0001; PLL vs. hSkin: *P* > 0.05). **h**–**k** Neutralization of EphrinB3 in acute MS lesion extracts (AL1-3) using antibodies raised against two distinct EphrinB3 epitopes (Ab1, Ab2) induced OPCs differentiation into O4 + and Mbp + oligodendrocytes as compared to treatment with unspecific antibodies (IgG); (*n* = 3; ANOVA: O4 ***P < 0.0001, Mbp ***P < 0.0001; Dunnett’s post hoc test; MPE vs. MPE + Ab1 and Ab2: *P* < 0.0001; MPE vs. MPE + IgG; *P* > 0.1). **l**–**o** Similarly, treatment of chronic active MS lesion extracts (MS1–3) with EphrinB3-specific antibodies (Ab1, Ab2) significantly increased in the percentage of O4 + and Mbp + OPCs as compared to IgG controls (*n* = 3; ANOVA: O4 ***P < 0.0001, Mbp ***P < 0.0001; Dunnett’s post hoc test; MPE vs. MPE + Ab1 and Ab2: *P* < 0.0001; MPE vs. MPE + IgG; *P* > 0.1). *Error bars* ± SEM. *Scale bar* in **e**–**g**, **i**–**k**, **m**–**o** = 30 μm

We first asked whether protein extracts from subacute (chronic active) MS lesions contain EphrinB3. Immunoblots with antibodies specific to EphrinB3 confirmed the presence of EphrinB3 MS lesions (Fig. 7c). Furthermore, proteomic analysis of the lesion extracts demonstrated high levels of myelin proteins in demyelinated lesions (Fig. 7a, b; Supplementary Tables 2–6).

To confirm that the axons within the lesions were indeed demyelinated, electron micrographs were obtained from sections of the same lesions. As expected from the absence of MOG staining, the axons within the lesions were denuded and only very few remnants of myelin sheaths were found in association with individual axons (Supplementary Fig. 6f–h). As phagocytosing cells were not detected in the lesions, macrophage-ingested myelin cannot account for the myelin-associated proteins identified by mass spectrometry. However, in all images obtained of all the lesions investigated, the presence of extracellular granular material was noted. In some areas, this was distributed diffusely, in other areas clumped together in small pockets between cellular processes (Supplementary Fig. 6f, g). In the absence of intact myelin sheaths and phagocytic cells, the myelin proteins detected were most likely associated with this interstitial debris. These findings support the hypothesis that demyelination in MS results in accumulation of extracellular myelin debris [30]. Moreover, the fact that EphrinB3 (and other myelin molecules) were found in subacute lesions indicated that the phagocytic clearance of myelin debris in MS lesions may remain incomplete.

### The molecular environment of early MS lesions inhibits OPC differentiation

We next sought to investigate whether EphrinB3 was present in MS lesions at biologically relevant levels. A number of OPC inhibiting factors have been identified, which seem to be re-expressed in MS lesions. However, the impact of the molecular environment of MS lesions on OPC differentiation has not yet been studied [1, 12, 13, 27, 30, 51]. We therefore cultured OPCs on substrates prepared from the extracts from acute and subacute MS lesions. Unlike control protein extracts from human skin samples or poly-l-lysine (PLL)-only controls, active and chronic active MS lesion extracts induced a significant inhibition of OPC differentiation (Fig. 7a–d) (extracts from normal-appearing CNS white matter were included as inhibitory controls. Due to the presence of myelin proteins, these were expected to inhibit OPC differentiation). TUNEL assays demonstrated that the MS lesion extracts specifically inhibited OPC differentiation as no differences in cell survival were detected (Supplementary Fig. 7d, e).

### Neutralization of EphrinB3 epitopes in MS lesion extracts promotes OPC differentiation

Our data provide the first direct evidence that the molecular milieu of MS lesions inhibits the differentiation of OPCs. But how important is the contribution of EphrinB3 to the differentiation-inhibiting environment of MS lesions?

To address this question, we investigated the effects of antibody-mediated masking of EphrinB3 epitopes in MS lesions. OPCs were cultured on lesion extracts from acute and subacute (chronic active) lesions that were incubated with antibodies binding EphrinB3 or unspecific IgG.

We found that treatment of anti EphrinB3-antibodies efficiently reduced the inhibitory effects of acute MS lesion extracts (Fig. 7h–k). Consistent with the presence of EphrinB3 in chronic active lesions, we found that treatment with anti EphrinB3-antibodies was also able to induce OPC differentiation on extracts from later lesion stages (Fig. 7l–o). However, the inhibitory activity in chronic active lesions was less pronounced. This may be in part explained by the different lysis buffer used for extraction of proteins, which also allowed for proteomic analysis of the lesion content. An alternative hypothesis is that the lesion composition, and specifically the presence of OPC differentiation inhibitors in MS lesions changes over time [30]. In conclusion, our data suggest that inhibitory effects of myelin debris accumulating in acute MS lesions can be neutralized by antibody-mediated masking of EphrinB3 epitopes.

## Discussion

We and others have in the past demonstrated that the innate immune system plays an important role in the process of CNS remyelination. [29, 37]. Apart from the secretion of cytokines [31, 37, 44], the phagocytic removal of myelin debris by macrophages is an essential prerequisite for successful remyelination in experimental models [28]. However, the factors in myelin responsible for the OPC differentiation block remained unknown. In the present manuscript, we demonstrate that EphrinB3 plays an important role in the myelin-related inhibition of OPCs. Ephrin signalling in OPCs has not yet been investigated in detail: a previous study has implicated Ephrin signalling in OPC migration [40]. Our data indicate that EphrinB3 induces a concentration-dependent inhibition of OPC differentiation in vitro. Furthermore, highly reminiscent to the infusion of myelin extracts [28], the addition of EphrinB3 into demyelinating lesions resulted in a nearby complete block of remyelination in the toxin model used. Conversely, genetic elimination or antibody-mediated neutralization of EphrinB3 epitopes decreased the inhibitory activity of myelin extracts on OPCs, promoted OPC differentiation in vitro and during development in vivo, and accelerated CNS remyelination in vivo. Finally, our data demonstrate that acute and subacute MS lesion extracts contain inhibitors of OPC differentiation that can be partially neutralized by blocking EphrinB3 epitopes.

Together with NogoA, OMgp, and MAG, EphrinB3 is known as a myelin-associated inhibitor of neurite outgrowth [4, 18]. This is in contrast to our findings in the oligodendrocyte lineage where only EphrinB3 negatively affected OPC differentiation whereas NogoA, OMgp and MAG did not [48]. Ephrin-Eph signalling is characterized by high levels of redundancy. Although we found that EphA4-RTK is responsive to EphrinB3 and expressed at significant levels in OPCs, it is possible that other receptors may also play a role. A specific importance of EphA4 in mediating EphrinB3 signals is suggested by the phenotypic similarities of EphrinB3-knockout and Eph4A-knockout mice, which both display similar “hopping” behaviour due to axonal divergence as a consequence of the lost midline barrier in the developing spinal cord [33]. Genetic disruption of EphrinB3-EphA4 signalling results in aberrant axonal sprouting, and aberrant corticospinal tract innervation due to the absence of inhibitory midline cues in the developing spinal cord. As a consequence, mice lacking EphrinB3 or EphA4 are unable to control their hindlimbs independently. Our study is the first demonstrating that EphrinB3-ko mice also exhibit changes in myelination.

We found that the presence of EphrinB3 induced inhibitory RhoA and PKCα signalling in OPCs. In the past, we demonstrated that both cascades are responsible for the differentiation-inhibiting effects of myelin preparations, and that modulation these cascades is able to overcome the differentiation block mediated by myelin in OPCs. A drawback of the present study is that it does not investigate whether a causal relationship exists between RhoA and PKCα and the inhibitory effects of EphrinB3 on OPC differentiation. Instead, our priority was to investigate whether neutralizing its effect affects OPC differential and CNS remyelination and to clarify its role in MS lesions.

The notion that EphrinB3 plays an important role for the myelin-associated inhibition of OPC differentiation is further supported by the increasing presence of EphrinB3 in sub-fractioned myelin extracts enriched for inhibitory activity. Furthermore, we found that the inhibitory activity in myelin extracts generated from EphrinB3 ko mice was reduced but not completely eliminated. Genetic deletion of EphrinB3 is known to result in a compensatory increase of EphrinB1 and EphrinB2 expression [43]. EphrinB1 and B2 also inhibit OPC differentiation, albeit to a lesser extent (Fig. 2b). It is therefore not surprising that residual inhibitory activity was found in myelin from EphrinB3-ko animals.

A number of findings indicate that EphrinB3 also plays an important role in the differentiation block occurring in MS. Failure of CNS remyelination in MS lesions has been associated with a poorly understood inability of OPCs to differentiate into myelin-forming oligodendrocytes [11, 32]. It has been proposed that both intrinsic and extrinsic factors are responsible for this failure of OPC differentiation. So far the evidence that extrinsic factors may play a role for the differentiation block in MS was limited to detecting their expression in subacute and chronic MS lesions [1, 12, 13, 27, 30, 51]. In contrast, our data provide the first direct evidence that the molecular environment of MS lesions is inhibitory for OPC differentiation. We found that antibody-mediated masking of EphrinB3 epitopes was able to partially neutralize the inhibitory effects of the lesion environment on OPCs. It is likely that other extrinsic factors, including, e.g. hyaluronan also contribute to the inhibition of OPCs in MS lesions [1, 46]. Our results emphasize the importance of the lesion environment for the success of remyelination, which must be taken into account when devising therapeutic strategies aimed at promoting myelin regeneration in MS.

In conclusion, our findings suggest that EphrinB3 may be a relevant target for promoting remyelination in demyelinating disease. Ephrin-Eph-RTK signalling lends itself to pharmacological intervention at the levels of ligand-receptor binding, receptor kinases and downstream signalling effectors. Several compounds interfering with downstream signalling pathways (such as the RhoA pathway) have entered the clinical testing stage. Small molecules and other pharmacological agents acting at the ligand-receptor and receptor-RTK levels are currently being developed [39]. Finally, the application of EphrinB-neutralizing antibodies could also be considered, as accumulation of myelin debris occurs during active lesion stages that are associated with an open blood–brain barrier [36].

## Electronic supplementary material

Below is the link to the electronic supplementary material.

Supplementary material 1 (DOCX 816 kb)

Supplementary material 2 (PDF 16648 kb)

Supplementary material 3 (PDF 2071 kb)

Supplementary material 4 (PDF 15153 kb)

Supplementary material 5 (PDF 12750 kb)

Supplementary material 6 (PDF 305 kb)

Supplementary material 7 (PDF 15525 kb)
