# Supplementary material for: Antibody-mediated neutralization of myelin-associated EphrinB3 accelerates CNS remyelination
Source: Acta Neuropathol. 2015 Dec 19;131(2):281–98. doi: 10.1007/s00401-015-1521-1 (PMC4713754; doi:10.1007/s00401-015-1521-1)
Supplement: Supplementary file 1 — Supplementary material 1 (DOCX 816 kb) [file 401_2015_1521_MOESM1_ESM.docx]

**Antibody mediated neutralization of myelin associated EphrinB3 accelerates CNS remyelination.**

Yasir A. Syed^1,3^, Chao Zhao^1^, Don Mahad^2^, Wiebke Möbius^3^, Friedrich Altmann^4^, Aycan Sentürk^5^, Franziska Foss^5^, Amparo Acker-Palmer^5^, Gert Lubec^6^, Kathryn Lilley^7^, Robin J.M. Franklin^1^, Klaus-A. Nave^3^, Mark R. N. Kotter^1,3,8^*

Author’s affiliations:

^1^ Wellcome Trust and MRC Cambridge Stem Cell Institute, and Department of Clinical Neurosciences, University of Cambridge, Cambridge, CB2 0SZ, UK.

^2^ Centre for Neuroregeneration, Chancellor's Building, 49 Little France Crescent, Edinburgh, EH16 4SB, UK.

^3^ Max Planck Institute for Experimental Medicine, Department of Neurogenetics, 37075 Goettingen, Germany.

^4^ Department of Chemistry, University of Natural Resource and Life Sciences, Muthgasse 18, 1190 Vienna, Austria.

^5^ Frankfurt Institute for Molecular Life Sciences and Institute of Cell Biology and Neuroscience, Goethe University Frankfurt, Max-von-Laue-Str. 9, D-60438, Frankfurt am Main, Germany.

^6^ Department of Pediatrics, Medical University Vienna, Waehringer Guertel 18-20, 1090 Vienna, Austria.

^7^ Cambridge Centre for Proteomics, Department of Biochemistry, University of Cambridge, Tennis Court Road, Cambridge, CB2 1QW, UK.

^8^ Universitätsmedizin Göttingen, Universitätsklinik für Neurochirurgie, Robert-Koch-Straße 40, 37075 Göttingen, Germany.

^*^To whom correspondence should be addressed

Mark R. Kotter MD PhD

Wellcome Trust and MRC Cambridge Stem Cell Institute,

Anne McLaren Laboratory,

University of Cambridge,

Cambridge, CB2 0SZ, UK

[mrk25@cam.ac.uk](mailto:mrk25@cam.ac.uk)

Tel: +44 1223 747476

Fax: +44 1223 763350

**Supplemental Information**

**Supplementary Fig 1: Effect of EphrinB3 on survival, differentiated Oligodendrocytes and proliferation** (**a**) TUNEL assay showing no differences in cell death between cells exposed to control substrates (PLL), and substrates prepared with myelin protein extracts (MPE), and EphrinB3. This supports the notion that EphrinB3 selectively inhibits OPC differentiation (n = 3; ANOVA: P > 0.05). (**b**) Similarly, no changes with respect to OPC survival were found in OPCs cultured in the presence of rat/human myelin protein extracts as compared to PLL (n = 3; ANOVA: P > 0.05). (**c,d**) Representative images of OPCs differentiated in presence and absence of EphrinB3 immunostained for O4(Red), Mbp (green) and Olig2 (Cyan). (**e**) Bar graph depicting percentages of O4 and Mbp positive cells in PLL-controls after 72h differentiation, of cells that were initially cultured in control conditions and then changed to EphrinB3+IgG-Fc containing media (PLL(48h)▶︎EphrinB3+Fc(24h), and in cells cultured in the presence of clustered EphrinB3 during the entire period (EphrinB3+Fc(24h)). (3d differentiation; n = 3; ANOVA: Mbp ***P < 0.0001; Dunnett’s post-hoc test: PLL (72hr) vs. PLL(48h) EphrinB3-Fc (24h)). Representative images of cells visualized by immunocytochemistry for O4 (red) and Mbp (green) (**f**) after 72 hr of differentiation on PLL, (**g**) of cells initially cultured on PLL for 48h and subsequently in the presence of clustered EphrinB3 for 24h, (**h**) and of OPCs cultured in the presence of EphrinB3 for 72h. (**i**) Bar graph showing quantification of Olig2 and PCNA double-positive cells after 24hr of differentiation in the presence and absence of EphrinB3 (n = 3; Student t test; P > 0.05). Representative images of Olig2 (Red) and PCNA (green) double immunostaining on OPCs after 24 hr of differentiation on PLL (**j**) and EphrinB3 (**k**). Scale bars in C,D = 30 μm, F-G = 25μm, J-K= 35 μm.

**Supplementary Fig** **2:** **The inhibitory activity of myelin protein extracts can be enriched by column chromatography** (**a**) Flow chart of the three-step column chromatography protocol. Right, chromatograms of CM-HighQ, and S100 chromatography; fractions containing OPC-differentiation-inhibiting activity highlighted in green, salt elutions indicated by red line. (**b**) Bar graph indicating the percentage of O4^+^ OPCs cultured on substrates made of MPE and fractions obtained after CM, HighQ, and S100 column chromatography. (**c**) Immuno blot depicting EphrinB1 and EphrinB2 proteins in whole brain lysates (brain), myelin protein extracts (MPE), and after CM, HighQ sub-fractionation of MPE into inhibitory chromatographic fractions.

**Supplementary Fig 3: Expression of Ephrins and Eph-RTK receptors by oligodendrocyte lineage cells.** (**a**) RT-PCR on mRNA isolated from OPCs after 2 days of differentiation demonstrating expression of Ephrins and Eph receptors (internal control: Gapdh; Brain: whole brain extracts). (**b-m**) Mature oligodendrocytes following 5d differentiation characterized by expression of Mbp and formation of membranous sheets expressing the RTKs EphA4 (**b,d**), EphB1 (**e,g**), EphB2 (**h,j**) and EphB3 (**k,m**).
Scale bars in B-M = 50 μm.

**Supplementary Fig 4: Experimental design of *in vivo* experiments.** (**a**) Experimental design to test the effects of EphrinB3 on CNS remyelination. Animals undergoing lesioning at day 0 were randomly assigned to 4 groups. At 10 dpi IgG-Fc infusion was initiated in control animals whereas treatment-group animals received pre-clustered EphrinB3. An additional control group only received PBS infusions. 28 dpi animals were sacrificed and tissue was processed for analysis. A fourth group of animals sacrificed at 10 dpi was included to serve as the base line. (**b**) Schematic diagram showing the location of the caudal cerebellar peduncle (CCP). Demyelination was induced by stereotactic administration of ethidium bromide (EB) into the CCP; at the time of surgery an infusion cannula was also stereotactically placed targetting the same lesion and secured with acrylic glue. At 10 dpi, osmotic minipumps containing Fc-IgG or pre-clustered EphrinB3 were connected via a cannula and placed subcutaneously. (**c**) To confirm adequate delivery via the cannula, a dye experiment was conducted. Overlap of Eosin (red) directly injected via the stereotactic syringe and Evan’s blue administered via the stereotactically placed cannula in the CCP confirmed adequate administration. (**d**) Infusion of pre-clustered EphrinB3 into *intact* CCP white matter via osmotic minipumps at the concentration used for the experiment did not result in detectable morphological changes in the CCP. (**e**) Immuno-histochemistry at 28 dpi confirming the delivery of EphrinB3 into CCP lesions. (**f**) Infusion of EphrinB3 did not affect the lesion size (solochrome-cyanine-stained sections). 10 dpi: n = 44; IgG-Fc: n = 35; EphrinB3-Fc-IgG: n = 52; t-test P > 0.05. (**g,h**) Representative images of IgG-Fc and EphrinB3-Fc-IgG infused lesions. (**i**) Experimental design for studying the effects of anti-EphrinB3 antibody treatment on CNS remyelination. (**j**) Schematic diagram visualising the peptide range against which the two anti-EphrinB3 antibodies were raised.
Error bars indicate ± SEM. Scale bars in C = 1mm, D = 30 μm; in E = 100 μm; in G,H = 10 μm.

**Supplementary Fig 5 : Quantification of demyelination and axonal density in EphrinB3-antibody infused rats and controls.**

**(a**) Manual counts to establish the percentage of demyelinated and remyelinated axons demonstrated an increase in the number of demyelinated axons in EphrinB3 infused lesion. No difference was observed in level of demyelinated axons for vehicle PBS and IgG-Fc control (PBS n=5; IgG-Fc control n = 6; EphrinB3 n = 5, t test,IgG-Fc vs EphrinB3 ***P < 0.001). This corresponding well with the observed decrease of remyelination (Figure 4N). (**b**) The density of axons between groups remained comparable (IgG-Fc control n = 6; EphrinB3 n = 5, t test, P > 0.001). (**c**) Enhanced remyelination observed in EphrinB3 Ab(1+2) infused animals (Figure 6F) are associated with a corresponding decrease in the number of demyelinated axons as compared to controls (Control-IgG: n = 4, EphrinB3-Ab1+2: n = 4, t test, **P < 0.01; Control-IgG: n = 4, EphrinB3-Ab1+2: n = 4, t test, P > 0.01). (**d**) The density of axons also remained comparable between the groups.

**Supplementary Fig 6 : Experimental strategy.** (**a**) MS lesion containing tissue samples were split into three parts and used as follows. 1) The lesions were characterized by immunohistochemistry. 2) Tissue was embedded for ultrastructural analysis by electron microscopy, and 3) tissue was used for protein extraction and subsequent testing on OPCs and for protein identification by LC-MS/MS. **b**) To selectively obtain tissue from the core of MS lesions intact myelin sheaths were visualized using a rapid immunohistochemistry protocol for myelin oligodendrocyte glycoprotein (MOG) and the unstained lesion core was harvested using a laser capture micro-dissector. (**c**) Protein extracted from the lesions, perilesional white matter and intact white matter were separated by SDS-PAGE and visualized by silver staining. Proteins contained in gel fragments outlined in black boxes were extracted and subjected to mass spectrometric analysis. (**d,e**) TUNEL staining showed no significant difference in number of cells undergoing apoptosis on control substrate (PLL) and in presence of active and chronic active MS lesion extracts. (n = 3; ANOVA; P > 0.05). (**f-h**) Electron micrographs of the same chronic active MS lesions show demyelinated axons (black arrowheads), astroglial processes (blue arrowheads), and extracellular debris (red arrowheads). (**I**) Immunohistochemistry demonstrating the presence of degraded MBP (dMBP, green) but not intact MBP (red) in chronic active MS lesions. (Lesion edge demarcated by white dotted line. (**j,k**) Representative images of GFAP (red) and EphA4 (green) double IHC in the chronic active MS lesion indicates a weak expression EphA by astrocytes.

Scale Bar in (F-H) = 5 μm; in I ,J = 100 μm; in K= 20 μm;

| **Protein Accession Number** | **Uniprot Entry** | **Protein Description** | **Sub cellular location** | **Protein score** | **Protein Mass** | **Protein Matches** | **Peptide Query** |
| --- | --- | --- | --- | --- | --- | --- | --- |
|  |  |  |  |  |  |  |  |
| gi\|70166245 | P02688 | myelin basic protein isoform 1 [Rattus norvegicus] | Membrane | 7850 | 21546 | 457 | 6344 |
| gi\|70166270 | P02688 | myelin basic protein isoform 4 [Rattus norvegicus] | Membrane | 7437 | 17230 | 437 | 6344 |
| gi\|8393759 | P02688 | myelin basic protein isoform 5 [Rattus norvegicus] | Membrane | 6293 | 14202 | 370 | 6344 |
| gi\|6981420 | B3GVW5 | protease, serine, 1 (trypsin 1) [Rattus norvegicus] | Membrane | 8007 | 26627 | 332 | 42133 |
| gi\|13591880 | P60203 | proteolipid protein (myelin) 1 [Rattus norvegicus] | Membrane | 3434 | 30855 | 173 | 2656 |
| gi\|149049391 | B1WBM0 | CD9 [Rattus Norvegicus] | Membrane | 1583 | 18283 | 173 | 1561 |
| gi\|158138498 | P09812 | muscle glycogen phosphorylase [Rattus norvegicus] | Membrane | 3811 | 97740 | 150 | 6772 |
| gi\|114052915 |  | cell adhesion molecule 4 [Rattus norvegicus] | Membrane | 3181 | 43211 | 122 | 2719 |
| gi\|11560135 |  | brain abundant, membrane attached signal protein 1 [Rattus norvegicus] | Membrane | 3577 | 21777 | 110 | 18313 |
| gi\|38014843 | Q62745 | Cd81 protein [Rattus norvegicus] | Membrane | 2542 | 26571 | 96 | 33801 |
| gi\|149026460 |  | brain abundant, membrane attached signal protein 1, isoform CRA_b [Rattus norvegicus] | Membrane | 2474 | 14162 | 92 | 18313 |
| gi\|266495 | P30009 | Myristoylated alanine-rich C-kinase substrate (MARCKS) (Protein kinase C substrate 80 kDa protein) | Membrane | 1792 | 29834 | 82 | 11352 |
| gi\|9507073 | P97546 | neuroplastin [Rattus norvegicus] | Membrane | 997 | 44246 | 69 | 1865 |
| gi\|149041842 | P97546 | stromal cell derived factor receptor 1, isoform CRA_a [Rattus norvegicus] | Membrane | 949 | 44703 | 60 | 1865 |
| gi\|7709992 | P26453 | basigin isoform 2 [Rattus norvegicus] | Membrane | 938 | 29852 | 59 | 1485 |
| gi\|8393415 | P07936 | growth associated protein 43 [Rattus norvegicus] | Membrane | 1291 | 23703 | 58 | 7096 |
| gi\|2497314 | Q63345 | Myelin-oligodendrocyte glycoprotein precursor | Membrane | 900 | 28149 | 51 | 1170 |
| gi\|149054231 | P13233 | cyclic nucleotide phosphodiesterase 1, isoform CRA_b [Rattus norvegicus] | Membrane | 483 | 45227 | 46 | 3069 |
| gi\|9296930 | O70352 | CD82 antigen (Metastasis suppressor homolog) | Membrane | 838 | 30152 | 39 | 8529 |
| gi\|6679186 | Q99P82 | claudin 11 [Mus musculus] | Membrane | 447 | 22841 | 26 | 26645 |
| gi\|13540687 | Q9EPH2 | MARCKS-like 1 [Rattus norvegicus] | Membrane | 212 | 19892 | 25 | 18783 |
| gi\|206392 | P13852 | prion-related protein | Membrane | 474 | 24781 | 23 | 8573 |
| gi\|203039 | Q00954 | Na+, K+ -ATPase beta subunit protein precursor | Membrane | 362 | 35610 | 23 | 15381 |
| gi\|69988 | P07722 | myelin-associated glycoprotein precursor, short splice form - rat | Membrane | 325 | 65223 | 23 | 401 |
| gi\|127723 | P06907 | Myelin P0 protein precursor (Myelin protein zero) (Myelin peripheral protein) (MPP) | Membrane | 271 | 27724 | 20 | 12431 |
| gi\|62079059 |  | BM88 antigen [Rattus norvegicus] | Membrane | 380 | 15091 | 19 | 17528 |
| gi\|149062471 | Q13277 | syntaxin 3, isoform CRA_b [Rattus norvegicus] | Membrane | 39 | 29989 | 19 | 2786 |
| gi\|157818703 | P14141 | carbonic anhydrase 14 [Rattus norvegicus] | Membrane | 367 | 37730 | 14 | 1406 |
| gi\|203246 | B0BNK7 | cell adhesion-like molecule [Rattus norvegicus] | Membrane | 265 | 37717 | 13 | 8259 |
| gi\|13929130 | Q3ZAV1 | solute carrier family 12 (sodium/potassium/chloride transporters), member 2 [Rattus norvegicus] | Membrane | 308 | 130954 | 11 | 17015 |
| gi\|18677765 | Q920Q0 | paralemmin [Rattus norvegicus] | Membrane | 167 | 42072 | 11 | 2979 |
| gi\|51948420 | Q6AY58 | B-cell receptor-associated protein 31 [Rattus norvegicus] | Membrane | 63 | 27951 | 11 | 9952 |
| gi\|14279176 | Q9JM53 | apoptosis-inducing factor [Rattus norvegicus] | Membrane | 41 | 66421 | 11 | 6855 |
| gi\|13928706 | P13596 | neural cell adhesion molecule 1 [Rattus norvegicus] | Membrane | 160 | 95398 | 10 | 5548 |
| gi\|220904 | P29419 | subunit d of mitochondrial H-ATP synthase [Rattus norvegicus] | Membrane | 148 | 18827 | 10 | 11057 |
| gi\|114052913 | Q925N6 | immunoglobulin superfamily, member 4D [Rattus norvegicus] | Membrane | 106 | 43946 | 10 | 3544 |
| gi\|32965004 | Q4L2A2 | CD99 [Rattus norvegicus] | Membrane | 173 | 16591 | 9 | 10252 |
| gi\|2506815 | P55068 | Brevican core protein precursor (Brain-enriched hyaluronan-binding protein) (Protein BEHAB) [Contai | Membrane | 146 | 97537 | 9 | 781 |
| gi\|2190166 | P97710 | BIT [Rattus norvegicus] | Membrane | 101 | 56297 | 8 | 6774 |
| gi\|3891604 | P04166 | Chain A, Rat Outer Mitochondrial Membrane Cytochrome B5 | Membrane | 202 | 10400 | 7 | 22611 |
| gi\|11133735 | Q9WVJ4 | Synaptojanin-2-binding protein (Mitochondrial outer membrane protein 25) (NPW16) | Membrane | 141 | 22606 | 7 | 1305 |
| gi\|25742796 | Q62813 | limbic system-associated membrane protein [Rattus norvegicus] | Membrane | 115 | 37814 | 7 | 3081 |
| gi\|12018246 | Q9JJW1 | tetraspanin 2 [Rattus norvegicus] | Membrane | 63 | 24801 | 7 | 21174 |
| gi\|149063937 | D4A768 | chemokine-like factor super family 5 (predicted), isoform CRA_b [Rattus norvegicus] | Membrane | 152 | 17753 | 6 | 40415 |
| gi\|13929058 |  | activated leukocyte cell adhesion molecule [Rattus norvegicus] | Membrane | 120 | 65665 | 6 | 11566 |
| gi\|47846864 |  | spermatogenic immunoglobulin superfamily [Rattus norvegicus] | Membrane | 109 | 47293 | 6 | 6756 |
| gi\|158636014 | Q9JM53 | apoptosis-inducing factor, mitochondrion-associated 2 [Rattus norvegicus] | Membrane | 71 | 40892 | 6 | 10377 |
| gi\|204795 | P01835 | immunoglobulin kappa-chain [Rattus sordidus] | Membrane | 223 | 11820 | 5 | 27816 |
| gi\|7672728 | Q91XV6 | phosphohippolin [Rattus norvegicus] | Membrane | 172 | 10454 | 5 | 32562 |
| gi\|9790115 | P62168 | frequenin homolog [Mus musculus] | Membrane | 157 | 21922 | 4 | 17170 |
| gi\|11612663 | P59649 | FXYD domain-containing ion transport regulator 7 [Rattus norvegicus] | Membrane | 141 | 8652 | 4 | 9792 |
| gi\|54400736 | Q5XIN6 | leucine zipper-EF-hand containing transmembrane protein 1 [Rattus norvegicus] | Membrane | 137 | 83635 | 4 | 15002 |
| gi\|6980972 |  | glutamate oxaloacetate transaminase 2 [Rattus norvegicus] | Membrane | 135 | 47683 | 4 | 3546 |
| gi\|203033 | P10719 | F1-ATPase beta subunit | Membrane | 118 | 38747 | 4 | 35386 |
| gi\|60678266 | P84039 | ectonucleotide pyrophosphatase/phosphodiesterase 5 [Rattus norvegicus] | Membrane | 108 | 54483 | 4 | 11072 |
| gi\|3184552 |  | syntaxin 13 [Rattus norvegicus] | Membrane | 97 | 30563 | 4 | 1879 |
| gi\|51948508 | Q68FQ2 | junctional adhesion molecule 3 [Rattus norvegicus] | Membrane | 90 | 35388 | 4 | 3029 |
| gi\|16758970 | O08957 | neuritin 1 [Rattus norvegicus] | Membrane | 90 | 15621 | 4 | 862 |
| gi\|6996589 | Q8VII6 | choline transporter-like protein [Rattus norvegicus] | Membrane | 82 | 74537 | 4 | 15851 |
| gi\|13431334 | Q9Z1Y3 | Cadherin-2 precursor (Neural cadherin) (N-cadherin) (CD325 antigen) | Membrane | 67 | 100081 | 4 | 5940 |
| gi\|42538984 | Q71LX6 | xin actin-binding repeat containing 2 [Rattus norvegicus] | Membrane | 40 | 375861 | 4 | 651 |
| gi\|33414042 | Q7TNM3 | oligodendrocyte-myelin glycoprotein [Rattus norvegicus] | Membrane | 38 | 49690 | 4 | 10202 |
| gi\|299036 | VDAC2_RAT | B-36 VDAC=36 kda voltage dependent anion channel [rats, hippocampus, Peptide, 295 aa] | Membrane | 69 | 32327 | 3 | 4590 |
| gi\|11968106 | Q9QZA6 | CD151 antigen (Raph blood group) [Rattus norvegicus] | Membrane | 65 | 29192 | 3 | 30291 |
| gi\|114158668 |  | similar to receptor expression enhancing protein 2 [Rattus norvegicus] | Membrane | 62 | 28361 | 3 | 678 |
| gi\|12004240 | Q91XT9 | ceramidase [Rattus norvegicus] | Membrane | 40 | 44741 | 3 | 3421 |
| gi\|62821825 |  | transmembrane protein 10 [Rattus norvegicus] | Membrane | 37 | 15777 | 3 | 47552 |
| gi\|47059104 | Q6P503 | ATPase, H+ transporting, V1 subunit G isoform 2 [Rattus norvegicus] | Membrane | 114 | 13716 | 2 | 22552 |
| gi\|32189350 |  | solute carrier family 25, member 5 [Rattus norvegicus] | Membrane | 64 | 33108 | 2 | 3606 |
| gi\|207308 | P01830 | thy-1 glycoprotein | Membrane | 58 | 16336 | 2 | 17847 |
| gi\|11968122 | P60203 | plasma membrane proteolipid [Rattus norvegicus] | Membrane | 54 | 19934 | 2 | 24562 |
| gi\|1838935 |  | mitochondrial precursor receptor [Rattus sp.] | Membrane | 54 | 16432 | 2 | 47337 |
| gi\|118150639 |  | shisa homolog 4 [Rattus norvegicus] | Membrane | 50 | 22194 | 2 | 7902 |
| gi\|6681095 | P62898 | cytochrome c, somatic [Mus musculus] | Membrane | 47 | 11712 | 2 | 12579 |
| gi\|21312151 |  | putative breast adenocarcinoma marker [Mus musculus] | Membrane | 40 | 25118 | 2 | 8948 |
| gi\|9506891 |  | solute carrier family 3, member 2 [Rattus norvegicus] | Membrane | 37 | 58150 | 2 | 9888 |
| gi\|8393180 | P10888 | cytochrome c oxidase subunit IV isoform 1 [Rattus norvegicus] | Membrane | 58 | 19559 | 1 | 24566 |
| gi\|258788 |  | H(+)-ATP synthase subunit e {N-terminal} [rats, liver, Peptide Mitochondrial Partial, 44 aa] | Membrane | 48 | 4964 | 1 | 20699 |
| gi\|61740629 | Q5BJN5 | coiled-coil-helix-coiled-coil-helix domain containing 4 [Rattus norvegicus] | Membrane | 45 | 15856 | 1 | 54067 |
| gi\|16758534 | O88775 | embigin [Rattus norvegicus] | Membrane | 45 | 37438 | 1 | 16950 |
| gi\|16758048 | RAGE_RAT | advanced glycosylation end product-specific receptor [Rattus norvegicus] | Membrane | 45 | 42979 | 1 | 27022 |
| gi\|57164091 | Q5M9I5 | ubiquinol-cytochrome c reductase hinge protein [Rattus norvegicus] | Membrane | 44 | 10702 | 1 | 33967 |
| gi\|6678051 | P61808 | stannin [Mus musculus] | Membrane | 44 | 9666 | 1 | 35197 |
| gi\|24233541 | P11240 | cytochrome c oxidase, subunit Va [Rattus norvegicus] | Membrane | 44 | 16347 | 1 | 3343 |
| gi\|20302061 | Q06647 | mitochondrial ATP synthase, O subunit [Rattus norvegicus] | Membrane | 43 | 23440 | 1 | 11384 |
| gi\|1000439 | P48721 | grp75 [Rattus sp.] | Membrane | 41 | 73984 | 1 | 15433 |
| gi\|62079189 | Q5I0D1 | glyoxalase domain containing 4 [Rattus norvegicus] | Membrane | 39 | 33532 | 1 | 7016 |
| gi\|109483174 | B4F795 | PREDICTED: similar to choline transporter-like protein 2 [Rattus norvegicus] | Membrane | 39 | 79420 | 1 | 18814 |
| gi\|6647578 | P70580 | Membrane-associated progesterone receptor component 1 (Acidic 25 kDa protein) (25-DX) (Ventral midl | Membrane | 39 | 21699 | 1 | 26569 |
| gi\|1223894 | Q642F4 | Sec22 homolog | Membrane | 38 | 29152 | 1 | 8191 |
| gi\|20302073 | F1M7X3 | cadherin 13 [Rattus norvegicus] | Membrane | 37 | 78436 | 1 | 17062 |
|  |  |  |  |  |  |  |  |
|  |  |  |  |  |  |  |  |
| gi\|8393910 | P31044 | phosphatidylethanolamine binding protein [Rattus norvegicus] | Cytoplasm | 9022 | 20902 | 230 | 19717 |
| gi\|203658 | P07632 | Cu-Zn superoxide dismutase (EC 1.15.1.1) | Cytoplasm | 4085 | 15871 | 173 | 3794 |
| gi\|6755983 | P62762 | visinin-like 1 [Mus musculus] | Cytoplasm | 2243 | 22299 | 125 | 3400 |
| gi\|13242237 | P63018 | heat shock protein 8 [Rattus norvegicus] | Cytoplasm | 1366 | 71055 | 81 | 1126 |
| gi\|61557085 | P16086 | spectrin beta 2 [Rattus norvegicus] | Cytoplasm | 1676 | 274328 | 71 | 4602 |
| gi\|204570 |  | major beta-hemoglobin | Cytoplasm | 1408 | 16097 | 67 | 8703 |
| gi\|5030428 | P47819 | glial fibrillary acidic protein delta [Rattus norvegicus] | Cytoplasm | 1540 | 48809 | 49 | 10023 |
| gi\|8048915 | Q62670 | beta 2 globin [rats, Sprague-Dawley, Peptide, 146 aa] | Cytoplasm | 1075 | 15965 | 48 | 8703 |
| gi\|71042796 | P61023 | Chain A, The Crystal Structure Of Calcineurin B Homologous Proein 1 (Chp1) | Cytoplasm | 281 | 23937 | 39 | 18061 |
| gi\|6981010 | P01946 | hemoglobin alpha 1 chain [Rattus norvegicus] | Cytoplasm | 556 | 15490 | 38 | 4413 |
| gi\|1698704 | P97595 | mast cell protease 9 | Cytoplasm | 150 | 26754 | 38 | 45051 |
| gi\|157823391 | Q6PCT3 | tumor protein D52 [Rattus norvegicus] | Cytoplasm | 1329 | 24311 | 36 | 10909 |
| gi\|4501885 | P63259 | beta actin [Homo sapiens] | Cytoplasm | 792 | 42052 | 36 | 2584 |
| gi\|16758846 | O08839 | bridging integrator 1 [Rattus norvegicus] | Cytoplasm | 616 | 64721 | 34 | 13304 |
| gi\|16758644 | P11232 | thioredoxin [Rattus norvegicus] | Cytoplasm | 942 | 12008 | 29 | 3403 |
| gi\|13591886 | P34926 | microtubule-associated protein 1 A [Rattus norvegicus] | Cytoplasm | 480 | 300831 | 29 | 11466 |
| gi\|81894378 | Q7TMA5 | Apolipoprotein B-100 precursor (Apo B-100) [Contains: Apolipoprotein B-48 (Apo B-48)] | Cytoplasm | 38 | 537740 | 28 | 1500 |
| gi\|112889 | P17475 | Alpha-1-antiproteinase precursor (Alpha-1-antitrypsin) (Alpha-1-proteinase inhibitor) | Cytoplasm | 174 | 46278 | 27 | 844 |
| gi\|27806017 | Q5PQN0 | neurocalcin delta [Bos taurus] | Cytoplasm | 478 | 22345 | 26 | 4154 |
| gi\|6755588 | P60881 | synaptosomal-associated protein 25 [Mus musculus] | Cytoplasm | 551 | 23528 | 24 | 13812 |
| gi\|4501881 | Q9Z1P2 | actin, alpha 1, skeletal muscle [Homo sapiens] | Cytoplasm | 386 | 42366 | 24 | 2584 |
| gi\|28948873 |  | Chain A, Crystal Structure Of Endothelial Nitric Oxide Synthase Peptide Bound To Calmodulin | Cytoplasm | 365 | 16515 | 24 | 1582 |
| gi\|21668480 | Q3ZB98 | band83 [Rattus norvegicus] | Cytoplasm | 292 | 31256 | 24 | 5221 |
| gi\|7949055 | P62749 | hippocalcin-like 1 [Mus musculus] | Cytoplasm | 442 | 22438 | 23 | 2674 |
| gi\|149045175 | G5BAT4 | desmoplakin, isoform CRA_b [Rattus norvegicus] | Cytoplasm | 209 | 329694 | 23 | 983 |
| gi\|396270 | P14659 | heat shock protein 70 [Rattus norvegicus] | Cytoplasm | 612 | 70328 | 22 | 4640 |
| gi\|395937 | Q62670 | 0 beta-2 globin [Rattus norvegicus] | Cytoplasm | 331 | 16026 | 21 | 8743 |
| gi\|157818715 | D4A3P1 | ubiquilin 4 [Rattus norvegicus] | Cytoplasm | 45 | 63624 | 20 | 34740 |
| gi\|55622 | P23565 | alpha-internexin [Rattus norvegicus] | Cytoplasm | 701 | 55712 | 16 | 5452 |
| gi\|11968070 | P45479 | palmitoyl-protein thioesterase [Rattus norvegicus] | Cytoplasm | 411 | 34946 | 16 | 5283 |
| gi\|11560002 | O08838 | amphiphysin [Rattus norvegicus] | Cytoplasm | 312 | 74946 | 16 | 8969 |
| gi\|25742763 |  | heat shock protein 5 [Rattus norvegicus] | Cytoplasm | 379 | 72473 | 15 | 7937 |
| gi\|2501106 | Q63754 | Beta-synuclein (Phosphoneuroprotein 14) (PNP 14) | Cytoplasm | 285 | 14495 | 15 | 1334 |
| gi\|241081 | P37377 | synuclein SYN2 [Rattus sp.] | Cytoplasm | 529 | 15859 | 13 | 1334 |
| gi\|6981600 | P61265 | syntaxin 1B2 [Rattus norvegicus] | Cytoplasm | 442 | 33452 | 13 | 6365 |
| gi\|203246 | B0BNK7 | cell adhesion-like molecule [Rattus norvegicus] | Cytoplasm | 265 | 37717 | 13 | 8259 |
| gi\|149054217 | Q6P0K8 | junction plakoglobin, isoform CRA_b [Rattus norvegicus] | Cytoplasm | 160 | 72254 | 13 | 8543 |
| gi\|220924 | Q00981 | ubiquitin carboxyl-terminal hydrolase PGP9.5 [Rattus norvegicus] | Cytoplasm | 145 | 25108 | 13 | 2770 |
| gi\|8393861 | P35332 | hippocalcin-like 4 [Rattus norvegicus] | Cytoplasm | 122 | 22402 | 13 | 3400 |
| gi\|31982030 | Q5XI73 | Rho GDP dissociation inhibitor (GDI) alpha [Mus musculus] | Cytoplasm | 303 | 23450 | 12 | 16498 |
| gi\|149027867 | G3V964 | rCG22798 [Rattus norvegicus] | Cytoplasm | 250 | 38559 | 12 | 8259 |
| gi\|19111164 |  | SMT3 supressor of mif two 3 homolog 2 [Mus musculus] | Cytoplasm | 175 | 10921 | 11 | 8619 |
| gi\|157823235 | DNJB6_RAT | DnaJ (Hsp40) homolog, subfamily C, member 15 [Rattus norvegicus] | Cytoplasm | 38 | 15981 | 11 | 1432 |
| gi\|149034106 | P37377 | synuclein, gamma, isoform CRA_a [Rattus norvegicus] | Cytoplasm | 266 | 11998 | 10 | 3654 |
| gi\|46485429 |  | glyoxalase 1 [Rattus norvegicus] | Cytoplasm | 204 | 20977 | 10 | 2644 |
| gi\|1050930 | P0CG51 | polyubiquitin [Rattus norvegicus] | Cytoplasm | 168 | 11234 | 10 | 33940 |
| gi\|1051270 | P63102 | 14-3-3 zeta isoform | Cytoplasm | 299 | 27955 | 9 | 11670 |
| gi\|31745160 | O70593 | small glutamine rich protein with tetratricopeptide repeats 2 [Rattus norvegicus] | Cytoplasm | 152 | 33751 | 9 | 8318 |
| gi\|56090475 | Q6PEC4 | S-phase kinase-associated protein 1A [Rattus norvegicus] | Cytoplasm | 382 | 18831 | 8 | 24388 |
| gi\|7949027 | Q6AYU3 | DnaJ (Hsp40) homolog, subfamily C, member 5 [Mus musculus] | Cytoplasm | 167 | 22885 | 8 | 26308 |
| gi\|11693154 | P63004 | platelet-activating factor acetylhydrolase alpha 2 subunit [Rattus norvegicus] | Cytoplasm | 152 | 25736 | 8 | 3436 |
| gi\|14134101 | P04692 | tropomyosin alpha isoform [Rattus norvegicus] | Cytoplasm | 61 | 28553 | 8 | 17026 |
| gi\|13929098 | P19527 | neurofilament, light polypeptide [Rattus norvegicus] | Cytoplasm | 49 | 61355 | 7 | 23237 |
| gi\|576017 |  | Chain E, Crystal Structures Of Rat Anionic Trypsin Complexed With The Protein Inhibitors Appi And B | Cytoplasm | 46 | 24467 | 7 | 12997 |
| gi\|538426 | P48500 | triosephosphate isomerase | Cytoplasm | 126 | 27417 | 6 | 10978 |
| gi\|11182065 | P15146 | microtubule associated protein (MAP) [Rattus norvegicus] | Cytoplasm | 67 | 260006 | 6 | 12608 |
| gi\|6679227 | P63055 | Purkinje cell protein 4 [Mus musculus] | Cytoplasm | 198 | 6803 | 5 | 8712 |
| gi\|56605812 | Q5RJQ4 | sirtuin (silent mating type information regulation 2 homolog) 2 [Rattus norvegicus] | Cytoplasm | 116 | 39921 | 5 | 17154 |
| gi\|494573 | P02625 | Chain 1, Refined X-Ray Structure Of Rat Parvalbumin, A Mammalian Alpha-Lineage Parvalbumin, At 2.0 | Cytoplasm | 107 | 11787 | 5 | 2954 |
| gi\|149025116 |  | zinc binding alcohol dehydrogenase, domain containing 1, isoform CRA_b [Rattus norvegicus] | Cytoplasm | 69 | 38511 | 5 | 3049 |
| gi\|8394432 | P35704 | peroxiredoxin 2 [Rattus norvegicus] | Cytoplasm | 60 | 21941 | 5 | 799 |
| gi\|5690429 | Q99JD5 | cytosolic branch chain aminotransferase BCATc [Rattus norvegicus] | Cytoplasm | 44 | 41252 | 5 | 2124 |
| gi\|203474 | P07335 | creatine kinase | Cytoplasm | 166 | 42984 | 4 | 29315 |
| gi\|6978966 | Q9JMB2 | type II brain 4.1 minor isoform [Rattus norvegicus] | Cytoplasm | 159 | 107463 | 4 | 18229 |
| gi\|6756039 | A2A5N2 | tyrosine 3-monooxygenase/tryptophan 5-monooxygenase activation protein, theta polypeptide [Mus musc | Cytoplasm | 148 | 28046 | 4 | 16828 |
| gi\|157823715 | Q5PPN5 | tubulin polymerization promoting protein [Rattus norvegicus] | Cytoplasm | 132 | 23703 | 4 | 22950 |
| gi\|228542 | D3ZJP6 | myosin:SUBUNIT=regulatory light chain | Cytoplasm | 102 | 19693 | 4 | 16183 |
| gi\|158138568 | Q63036 | albumin [Rattus norvegicus] | Cytoplasm | 98 | 70710 | 4 | 11569 |
| gi\|56199809 | Q5QD51 | A kinase anchoring protein 12 gamma [Rattus norvegicus] | Cytoplasm | 92 | 170601 | 4 | 1769 |
| gi\|62543513 |  | zinc binding alcohol dehydrogenase, domain containing 1 [Rattus norvegicus] | Cytoplasm | 69 | 30220 | 4 | 3049 |
| gi\|13624295 | Q63228 | glia maturation factor, beta [Rattus norvegicus] | Cytoplasm | 117 | 16897 | 3 | 3930 |
| gi\|109471992 | D3ZA73 | PREDICTED: similar to contactin associated protein-like 2 isoform a [Rattus norvegicus] | Cytoplasm | 106 | 100219 | 3 | 24881 |
| gi\|8394009 | P10111 | peptidylprolyl isomerase A [Rattus norvegicus] | Cytoplasm | 105 | 18091 | 3 | 10526 |
| gi\|117606182 | A0JN30 | canopy 2 homolog [Rattus norvegicus] | Cytoplasm | 94 | 21038 | 3 | 19619 |
| gi\|149047422 | Q63028 | adducin 1 (alpha), isoform CRA_c [Rattus norvegicus] | Cytoplasm | 71 | 25242 | 3 | 11560 |
| gi\|40018606 | Q6P7A9 | acid alpha-glucosidase [Rattus norvegicus] | Cytoplasm | 69 | 106880 | 3 | 34089 |
| gi\|56090241 | Q8CGV7 | thiamine triphosphatase [Rattus norvegicus] | Cytoplasm | 64 | 24585 | 3 | 16648 |
| gi\|8393896 | Q9Z0W5 | protein kinase C and casein kinase substrate in neurons 1 [Rattus norvegicus] | Cytoplasm | 61 | 50760 | 3 | 4152 |
| gi\|203237 | P07171 | calbindin-d28k | Cytoplasm | 48 | 30225 | 3 | 14845 |
| gi\|157786640 | Q5XHX6 | thioredoxin domain containing 17 [Rattus norvegicus] | Cytoplasm | 46 | 14368 | 3 | 1549 |
| gi\|17986258 | Q64119 | myosin, light chain 6, alkali, smooth muscle and non-muscle isoform 1 [Homo sapiens] | Cytoplasm | 43 | 17090 | 3 | 5238 |
| gi\|6753844 | G5B5C1 | fibroblast growth factor 11 [Mus musculus] | Cytoplasm | 42 | 25504 | 3 | 17439 |
| gi\|83642824 | A1L108 | actin related protein 2/3 complex, subunit 5-like [Rattus norvegicus] | Cytoplasm | 41 | 11228 | 3 | 10405 |
| gi\|11560131 | O08557 | dimethylarginine dimethylaminohydrolase 1 [Rattus norvegicus] | Cytoplasm | 40 | 31805 | 3 | 8297 |
| gi\|26023949 | P04764 | enolase 2, gamma, neuronal [Rattus norvegicus] | Cytoplasm | 89 | 47510 | 2 | 12866 |
| gi\|4505705 | Q5U318 | phosphoprotein enriched in astrocytes 15 [Homo sapiens] | Cytoplasm | 46 | 15088 | 2 | 40484 |
|  |  |  |  |  |  |  |  |
|  |  |  |  |  |  |  |  |
| gi\|25006237 | Q6IN37 | GM2 activator protein [Rattus norvegicus] | Nucleus | 383 | 21963 | 22 | 764 |
| gi\|226791 | P22057 | prostaglandin D synthetase | Nucleus | 594 | 21492 | 20 | 7801 |
| gi\|149049493 | D3ZDS4 | EMG1 nucleolar protein homolog (S. cerevisiae) (predicted), isoform CRA_a [Rattus norvegicus] | Nucleus | 109 | 19581 | 18 | 34760 |
| gi\|6981498 | P04631 | S100 protein, beta polypeptide, neural [Rattus norvegicus] | Nucleus | 367 | 10851 | 15 | 32190 |
| gi\|17865351 |  | valosin-containing protein [Rattus norvegicus] | Nucleus | 190 | 89977 | 9 | 1256 |
| gi\|67078430 | Q4V8G6 | methyltransferase-like 3 [Rattus norvegicus] | Nucleus | 43 | 65329 | 9 | 4602 |
| gi\|157823369 | Q5EAN7 | telomeric repeat binding factor 2 [Rattus norvegicus] | Nucleus | 38 | 56264 | 9 | 35788 |
| gi\|71480173 | Q5M844 | spectrin repeat containing, nuclear envelope 1 [Rattus norvegicus] | Nucleus | 39 | 924756 | 7 | 16525 |
| gi\|4504301 |  | histone cluster 1, H4a [Homo sapiens] | Nucleus | 88 | 11360 | 6 | 10882 |
| gi\|157820325 | O55099 | chromosome segregation 1-like [Rattus norvegicus] | Nucleus | 96 | 110942 | 5 | 3185 |
| gi\|157823383 | D3ZJ29 | zinc finger protein 512B [Rattus norvegicus] | Nucleus | 42 | 97576 | 4 | 20779 |
| gi\|226474 | O35986 | Zn binding protein | Nucleus | 111 | 11421 | 3 | 7960 |
| gi\|4504245 |  | histone cluster 1, H2ac [Homo sapiens] | Nucleus | 67 | 14097 | 3 | 1127 |
| gi\|149016335 | P13383 | nucleolin, isoform CRA_e [Rattus norvegicus] | Nucleus | 63 | 55321 | 3 | 18613 |
| gi\|223096 |  | histone H2B | Nucleus | 42 | 13766 | 3 | 12608 |
| gi\|54112117 |  | splicing factor 3b, subunit 1 isoform 1 [Homo sapiens] | Nucleus | 40 | 146479 | 3 | 3052 |
| gi\|81868408 |  | Leucine zipper protein 1 (Leucine zipper motif-containing protein) | Nucleus | 40 | 117675 | 3 | 454 |
| gi\|157823053 | E9PTS4 | minichromosome maintenance deficient 5, cell division cycle 46 [Rattus norvegicus] | Nucleus | 58 | 40196 | 2 | 5374 |
| gi\|82658782 | Q1PS21 | minichromosome maintenance protein 7 [Rattus norvegicus] | Nucleus | 55 | 81665 | 2 | 5452 |
| gi\|117940021 | P81795 | eukaryotic translation initiation factor 3, subunit J [Rattus norvegicus] | Nucleus | 40 | 29284 | 2 | 16994 |
| gi\|33468857 | P62959 | histidine triad nucleotide binding protein 1 [Mus musculus] | Nucleus | 56 | 13882 | 1 | 21170 |
| gi\|49169847 | Q6JP77 | A-kinase anchoring protein 18 ,isoform delta [Rattus norvegicus] | Nucleus | 54 | 39621 | 1 | 23573 |
| gi\|286206 | P61023 | calcineurin B [Rattus sp.] | Nucleus | 45 | 25078 | 1 | 33876 |
| gi\|6671746 | P45591 | cofilin 2, muscle [Mus musculus] | Nucleus | 42 | 18812 | 1 | 19276 |
| gi\|5931734 |  | H protein [Rattus norvegicus] | Nucleus | 40 | 18713 | 1 | 53231 |
| gi\|149060755 |  | apolipoprotein D, isoform CRA_a [Rattus norvegicus] | Secreted | 1672 | 23407 | 96 | 20365 |
| gi\|56090361 | Q5XII0 | ependymin related protein 1 [Rattus norvegicus] | Secreted | 610 | 26022 | 37 | 7593 |
| gi\|136467 | P02767 | Transthyretin precursor (Prealbumin) (TBPA) | Secreted | 165 | 15824 | 7 | 974 |
| gi\|1127275 |  | Chain A, Crystal Structures Of Recombinant Rat Cathepsin B And A Cathepsin B-Inhibitor Complex: Imp | Secreted | 118 | 28489 | 7 | 733 |
| gi\|8569265 | P60881 | Chain A, Crystal Structure Of The Neuronal T-Snare Syntaxin-1a | Secreted | 191 | 15000 | 5 | 5470 |
| gi\|27465565 | Q8CHN5 | epididymal secretory protein E1 [Rattus norvegicus] | Secreted | 92 | 16753 | 5 | 8686 |
| gi\|4894188 | Q63564 | vesicle associated membrane protein 2B [Rattus norvegicus] | Secreted | 165 | 14557 | 3 | 30767 |
| gi\|115720 | P24268 | Cathepsin D precursor [Contains: Cathepsin D 12 kDa light chain; Cathepsin D 9 kDa light chain; Cat | Secreted | 101 | 45165 | 2 | 34506 |
| gi\|115893 | P15087 | Carboxypeptidase E precursor (CPE) (Carboxypeptidase H) (CPH) (Enkephalin convertase) (Prohormone-p | Secreted | 68 | 53675 | 2 | 17943 |
| gi\|76780271 | Q3KR80 | Arylsulfatase A [Rattus norvegicus] | Secreted | 59 | 53288 | 1 | 12571 |
| gi\|203341 | P00786 | cathepsin H | Secreted | 52 | 33459 | 1 | 23380 |
| gi\|16751921 | Q71DI1 | dermcidin preproprotein [Homo sapiens] | Unknown | 642 | 11391 | 45 | 10444 |
| gi\|55825 |  | unnamed protein product [Rattus norvegicus] | Unknown | 578 | 16039 | 38 | 8703 |
| gi\|149024209 | B2RZ27 | SH3 domain binding glutamic acid-rich protein-like 3 (predicted), isoform CRA_a [Rattus norvegicus] | Unknown | 587 | 10543 | 29 | 6887 |
| gi\|109490823 |  | PREDICTED: similar to obscurin, cytoskeletal calmodulin and titin-interacting RhoGEF [Rattus norveg | Unknown | 140 | 729464 | 19 | 1937 |
| gi\|7514122 |  | gene rSSTR4 protein - rat (fragment) | Unknown | 58 | 1071 | 19 | 7765 |
| gi\|27716987 | P17078 | PREDICTED: similar to 60S ribosomal protein L35 [Rattus norvegicus] | Unknown | 38 | 14645 | 18 | 34305 |
| gi\|109482140 |  | PREDICTED: similar to oxidation resistance 1 [Rattus norvegicus] | Unknown | 134 | 87024 | 14 | 610 |
| gi\|158508473 |  | hypothetical protein LOC362090 [Rattus norvegicus] | Unknown | 41 | 49776 | 13 | 10084 |
| gi\|157823033 |  | hypothetical protein LOC294732 [Rattus norvegicus] | Unknown | 142 | 42278 | 10 | 4487 |
| gi\|109469965 |  | PREDICTED: similar to MORC family CW-type zinc finger 4 (Zinc finger CW-type coiled-coil domain pro | Unknown | 43 | 117594 | 10 | 14233 |
| gi\|109457636 |  | PREDICTED: similar to Band 4.1-like protein 2 (Generally expressed protein 4.1) (4.1G) [Rattus norv | Unknown | 233 | 147390 | 9 | 13534 |
| gi\|109487222 | G5B875 | PREDICTED: similar to neurobeachin-like 1 [Rattus norvegicus] | Unknown | 41 | 301180 | 8 | 15944 |
| gi\|157820337 |  | hypothetical protein LOC362368 [Rattus norvegicus] | Unknown | 138 | 21503 | 7 | 13817 |
| gi\|157820337 |  | hypothetical protein LOC362368 [Rattus norvegicus] | Unknown | 138 | 21503 | 7 | 13817 |
| gi\|62661785 |  | PREDICTED: similar to 60S acidic ribosomal protein P2 [Rattus norvegicus] | Unknown | 193 | 11699 | 5 | 15888 |
| gi\|149028719 |  | rCG58876, isoform CRA_a [Rattus norvegicus] | Unknown | 43 | 21306 | 5 | 38927 |
| gi\|109473713 |  | PREDICTED: hypothetical protein [Rattus norvegicus] | Unknown | 37 | 4089 | 5 | 19972 |
| gi\|57429 |  | unnamed protein product [Rattus norvegicus] | Unknown | 115 | 50387 | 4 | 8261 |
| gi\|109480904 | Q6MG53 | PREDICTED: similar to lymphocyte antigen 6 complex, locus H [Rattus norvegicus] | Unknown | 103 | 27388 | 3 | 26821 |
| gi\|157819293 |  | hypothetical protein LOC360617 [Rattus norvegicus] | Unknown | 46 | 12489 | 3 | 11889 |
| gi\|56621 |  | unnamed protein product [Rattus norvegicus] | Unknown | 38 | 199331 | 3 | 18387 |
| gi\|109459980 | D4A9E7 | PREDICTED: similar to ankyrin repeat domain 15 [Rattus norvegicus] | Unknown | 37 | 149915 | 3 | 39363 |
| gi\|62641302 | Q2MHH0 | PREDICTED: similar to tumor suppressor candidate 5 [Rattus norvegicus] | Unknown | 55 | 36980 | 2 | 21140 |
| gi\|109486556 |  | PREDICTED: similar to RAN binding protein 3 isoform RANBP3-b [Rattus norvegicus] | Unknown | 40 | 52694 | 2 | 125 |
| gi\|149059582 | B2GUV5 | rCG55259, isoform CRA_a [Rattus norvegicus] | Unknown | 59 | 6959 | 1 | 20381 |
| gi\|149024180 |  | rCG30697 [Rattus norvegicus] | Unknown | 42 | 41129 | 1 | 11590 |

**Supplementary** **Table 1:** LC-MCMC results - CM-HighQ-S100 Fraction

**Supplementary** **Table 2:** Proteins identified by LC-MSMS, sample WM1

| **Protein Accession Number** | **Uniprot Entry** | **Protein Description** | **Sub cellular location** | **Protein Score** | **Protein Mass** | **Protein matches** | **Protein coverage** |
| --- | --- | --- | --- | --- | --- | --- | --- |
| IPI00219661 | P60201 | PLP1 Isoform 1 of Myelin proteolipid protein | Myelin | 486 | 30855 | 53 | 29.2 |
| IPI00220993 | P09543 | CNP Isoform CNPI of 2~,3~-cyclic-nucleotide 3~-phosphodiesterase | Myelin | 998 | 45469 | 42 | 50.4 |
| IPI00556079 | Q16653 | MOG Isoform 1 of Myelin-oligodendrocyte glycoprotein | Myelin | 485 | 28560 | 12 | 30.8 |
| IPI00478921 | P25189 | MPZ myelin protein zero | Myelin | 229 | 28684 | 5 | 19.4 |
| IPI00021907 | P02686 | MBP Isoform 1 of Myelin basic protein | Myelin | 58 | 33097 | 3 | 5.9 |
| IPI00026237 | P20916 | MAG Myelin-associated glycoprotein | Myelin | 36 | 69880 | 1 | 1.3 |
| IPI00303476 | P06576 | ATP5B ATP synthase subunit beta, mitochondrial | Membrane | 778 | 56525 | 19 | 34.8 |
| IPI00216171 | P09104 | ENO2 Gamma-enolase | Membrane | 702 | 47581 | 19 | 34.6 |
| IPI00024853 | Q9BXM0 | PRX Isoform 1 of Periaxin | Membrane | 693 | 155248 | 15 | 8.8 |
| IPI00017855 | Q99798 | ACO2 Aconitate hydratase, mitochondrial | Membrane | 722 | 86113 | 14 | 20.4 |
| IPI00006482 | P05023 | ATP1A1 Isoform Long of Sodium/potassium-transporting ATPase subunit alpha-1 | Membrane | 714 | 114135 | 11 | 14 |
| IPI00302840 | P13637 | ATP1A3 Sodium/potassium-transporting ATPase subunit alpha-3 | Membrane | 699 | 113102 | 11 | 13.2 |
| IPI00418169 | P07355 | ANXA2 Isoform 2 of Annexin A2 | Membrane | 529 | 40671 | 9 | 28.6 |
| IPI00178352 | Q14315 | FLNC Isoform 1 of Filamin-C | Membrane | 529 | 293407 | 9 | 4.8 |
| IPI00013164 | P41219 | PRPH Isoform 1 of Peripherin | Membrane | 310 | 53732 | 9 | 14.3 |
| IPI00784154 | P10809 | HSPD1 60 kDa heat shock protein, mitochondrial | Membrane | 193 | 61187 | 9 | 7.3 |
| IPI00440493 | P25705 | ATP5A1 ATP synthase subunit alpha, mitochondrial | Membrane | 496 | 59828 | 8 | 17 |
| IPI00016801 | P00367 | GLUD1 Glutamate dehydrogenase 1, mitochondrial | Membrane | 279 | 61701 | 6 | 10.6 |
| IPI00219365 | P26038 | MSN Moesin | Membrane | 226 | 67892 | 5 | 9 |
| IPI00239405 | Q8WXH0 | SYNE2 Isoform 1 of Nesprin-2 | Membrane | 102 | 801817 | 5 | 0.4 |
| IPI00415044 | Q6PCT2 | FBXL19 Isoform 2 of F-box/LRR-repeat protein 19 | Membrane | 52 | 71002 | 5 | 2.9 |
| IPI00185038 | Q9NRD9 | DUOX1 Isoform 1 of Dual oxidase 1 | Membrane | 51 | 178719 | 5 | 1.1 |
| IPI00007682 | P38606 | ATP6V1A V-type proton ATPase catalytic subunit A | Membrane | 258 | 68660 | 4 | 9.9 |
| IPI00291006 | P40926 | MDH2 Malate dehydrogenase, mitochondrial | Membrane | 213 | 35937 | 4 | 16.9 |
| IPI00007765 | P38646 | HSPA9 Stress-70 protein, mitochondrial | Membrane | 183 | 73920 | 4 | 5.4 |
| IPI00179109 | Q8IXJ6 | SIRT2 Isoform 1 of NAD-dependent deacetylase sirtuin-2 | Membrane | 136 | 43782 | 4 | 7.2 |
| IPI00020599 | P27797 | CALR Calreticulin | Membrane | 88 | 48283 | 3 | 11 |
| IPI00031522 | P40939 | HADHA Trifunctional enzyme subunit alpha, mitochondrial | Membrane | 147 | 83688 | 2 | 4.2 |
| IPI00000190 | P60033 | CD81 CD81 antigen | Membrane | 136 | 26476 | 2 | 16.5 |
| IPI00013847 | P31930 | UQCRC1 Cytochrome b-c1 complex subunit 1, mitochondrial | Membrane | 126 | 53297 | 2 | 8.5 |
| IPI00006663 | P05091 | ALDH2 Aldehyde dehydrogenase, mitochondrial | Membrane | 124 | 56859 | 2 | 4.1 |
| IPI00007834 | Q01484 | ANK2 Isoform 1 of Ankyrin-2 | Membrane | 118 | 432499 | 2 | 0.6 |
| IPI00289758 | P17655 | CAPN2 Calpain-2 catalytic subunit | Membrane | 113 | 80814 | 2 | 2.1 |
| IPI00930179 | IPI00930179 | HLA-C MHC class I antigen (Fragment) | Membrane | 110 | 31820 | 2 | 14.7 |
| IPI00026053 | O75508 | CLDN11 Claudin-11 | Membrane | 104 | 22720 | 2 | 8.2 |
| IPI00215997 | P21926 | CD9 CD9 antigen | Membrane | 101 | 25969 | 2 | 14 |
| IPI00007812 | P21281 | ATP6V1B2 V-type proton ATPase subunit B, brain isoform | Membrane | 80 | 56807 | 2 | 2.7 |
| IPI00019888 | P51649 | ALDH5A1 Succinate-semialdehyde dehydrogenase, mitochondrial | Membrane | 74 | 58034 | 2 | 11.6 |
| IPI00022202 | Q00325 | SLC25A3 Isoform A of Phosphate carrier protein, mitochondrial | Membrane | 70 | 40525 | 2 | 3.3 |
| IPI00028946 | O95197 | RTN3 Isoform 3 of Reticulon-3 | Membrane | 62 | 25764 | 2 | 4.7 |
| IPI00783604 | Q6UWM0 | EPHA6 EPH receptor A6 isoform a | Membrane | 47 | 128248 | 2 | 1.3 |
| IPI00007411 | Q9UKA4 | AKAP11 A-kinase anchor protein 11 | Membrane | 47 | 212946 | 2 | 1.3 |
| IPI00013303 | Q13449 | LSAMP Limbic system-associated membrane protein | Membrane | 79 | 37883 | 1 | 4.7 |
| IPI00020984 | B4DGP8 | CANX cDNA FLJ55574, highly similar to Calnexin | Membrane | 78 | 71971 | 1 | 2.6 |
| IPI00157414 | Q6UWR7 | ENPP6 Ectonucleotide pyrophosphatase/phosphodiesterase family member 6 | Membrane | 67 | 50551 | 1 | 4.3 |
| IPI00293655 | Q92499 | DDX1 ATP-dependent RNA helicase DDX1 | Membrane | 67 | 83349 | 1 | 2.8 |
| IPI00022891 | P12235 | SLC25A4 ADP/ATP translocase 1 | Membrane | 54 | 33271 | 1 | 3 |
| IPI00015473 | P43003 | SLC1A3 Excitatory amino acid transporter 1 | Membrane | 52 | 59705 | 1 | 3.7 |
| IPI00037448 | Q9UBQ7 | GRHPR Glyoxylate reductase/hydroxypyruvate reductase | Membrane | 50 | 36045 | 1 | 7.6 |
| IPI00013421 | Q13491 | GPM6B Isoform 1 of Neuronal membrane glycoprotein M6-b | Membrane | 46 | 29882 | 1 | 4.9 |
| IPI00299571 | Q15084 | PDIA6 Isoform 2 of Protein disulfide-isomerase A6 | Membrane | 44 | 54380 | 1 | 4.9 |
| IPI00015911 | P09622 | DLD Dihydrolipoyl dehydrogenase, mitochondrial | Membrane | 37 | 54713 | 1 | 2.8 |
| IPI00297160 | P16070 | CD44 Isoform 12 of CD44 antigen | Membrane | 36 | 39904 | 1 | 2.2 |
| IPI00025363 | P14136 | GFAP Isoform 1 of Glial fibrillary acidic protein | Cytoplasm | 2889 | 49907 | 425 | 81 |
| IPI00237671 | P07196 | NEFL Neurofilament light polypeptide | Cytoplasm | 2050 | 61536 | 148 | 61.7 |
| IPI00180675 | Q71U36 | TUBA1A Tubulin alpha-1A chain | Cytoplasm | 1382 | 50788 | 97 | 59.9 |
| IPI00792677 | B4DDU2 | TUBA1B cDNA FLJ60097, highly similar to Tubulin alpha-ubiquitous chain | Cytoplasm | 1386 | 46797 | 95 | 64.9 |
| IPI00013475 | Q13885 | TUBB2A Tubulin beta-2A chain | Cytoplasm | 1551 | 50274 | 85 | 72.4 |
| IPI00910602 | P12036 | NEFH Isoform 1 of Neurofilament heavy polypeptide | Cytoplasm | 1528 | 112639 | 85 | 27.9 |
| IPI00031370 | Q9BVA1 | TUBB2B Tubulin beta-2B chain | Cytoplasm | 1526 | 50377 | 85 | 72.4 |
| IPI00007752 | P68371 | TUBB2C Tubulin beta-2C chain | Cytoplasm | 1471 | 50255 | 80 | 64.3 |
| IPI00909140 | Q13509 | TUBB Tubulin beta chain | Cytoplasm | 1415 | 50095 | 80 | 66.7 |
| IPI00217507 | P07197 | NEFM Neurofilament medium polypeptide | Cytoplasm | 1120 | 102468 | 76 | 22.4 |
| IPI00007750 | P68366 | TUBA4A Tubulin alpha-4A chain | Cytoplasm | 1182 | 50634 | 73 | 51.6 |
| IPI00021439 | P60709 | ACTB Actin, cytoplasmic 1 | Cytoplasm | 1195 | 42052 | 70 | 60 |
| IPI00023598 | P04350 | TUBB4 Tubulin beta-4 chain | Cytoplasm | 1398 | 50010 | 65 | 63.5 |
| IPI00013683 | Q13509 | TUBB3 Tubulin beta-3 chain | Cytoplasm | 1190 | 50856 | 64 | 49.1 |
| IPI00257508 | Q16555 | DPYSL2 Dihydropyrimidinase-related protein 2 | Cytoplasm | 1543 | 62711 | 59 | 59.8 |
| IPI00219018 | P04406 | GAPDH Glyceraldehyde-3-phosphate dehydrogenase | Cytoplasm | 1236 | 36201 | 47 | 66.6 |
| IPI00465248 | P06733 | ENO1 Isoform alpha-enolase of Alpha-enolase | Cytoplasm | 1520 | 47481 | 44 | 55.5 |
| IPI00418471 | P08670 | VIM Vimentin | Cytoplasm | 616 | 53676 | 41 | 26.6 |
| IPI00479186 | P14618 | PKM2 Isoform M2 of Pyruvate kinase isozymes M1/M2 | Cytoplasm | 1059 | 58470 | 34 | 39 |
| IPI00220644 | P14618 | PKM2 Isoform M1 of Pyruvate kinase isozymes M1/M2 | Cytoplasm | 1015 | 58538 | 34 | 37.5 |
| IPI00005614 | Q01082 | SPTBN1 Isoform Long of Spectrin beta chain, brain 1 | Cytoplasm | 1625 | 275237 | 32 | 15.5 |
| IPI00382470 | P07900 | HSP90AA1 Isoform 2 of Heat shock protein HSP 90-alpha | Cytoplasm | 918 | 98670 | 29 | 18.1 |
| IPI00008603 | P62736 | ACTA2 Actin, aortic smooth muscle | Cytoplasm | 540 | 42381 | 29 | 24.1 |
| IPI00414676 | P08238 | HSP90AB1 Heat shock protein HSP 90-beta | Cytoplasm | 788 | 83554 | 25 | 22.1 |
| IPI00219217 | P07195 | LDHB L-lactate dehydrogenase B chain | Cytoplasm | 915 | 36900 | 22 | 44.6 |
| IPI00022977 | P12277 | CKB Creatine kinase B-type | Cytoplasm | 666 | 42902 | 22 | 34.6 |
| IPI00221226 | P08133 | ANXA6 Annexin A6 | Cytoplasm | 974 | 76168 | 19 | 27.9 |
| IPI00004358 | P09104 | PYGB Glycogen phosphorylase, brain form | Cytoplasm | 735 | 97319 | 16 | 20.5 |
| IPI00294187 | Q9Y2J8 | PADI2 Protein-arginine deiminase type-2 | Cytoplasm | 630 | 76257 | 15 | 22.9 |
| IPI00003021 | P50993 | ATP1A2 Sodium/potassium-transporting ATPase subunit alpha-2 | Cytoplasm | 727 | 113505 | 13 | 16.1 |
| IPI00007702 | P54652 | HSPA2 Heat shock-related 70 kDa protein 2 | Cytoplasm | 642 | 70263 | 13 | 17.8 |
| IPI00215628 | P13611 | VCAN Isoform V1 of Versican core protein | Cytoplasm | 500 | 266882 | 13 | 4.3 |
| IPI00304925 | P08107 | HSPA1A;HSPA1B Heat shock 70 kDa protein 1A/1B | Cytoplasm | 534 | 70294 | 12 | 18.4 |
| IPI00010154 | P31150 | GDI1 Rab GDP dissociation inhibitor alpha | Cytoplasm | 517 | 51177 | 12 | 23.7 |
| IPI00843765 | Q13813 | SPTAN1 Isoform 3 of Spectrin alpha chain, brain | Cytoplasm | 501 | 282906 | 11 | 4.6 |
| IPI00465439 | P04075 | ALDOA Fructose-bisphosphate aldolase A | Cytoplasm | 498 | 39851 | 11 | 22 |
| IPI00014898 | Q15149 | PLEC1 Isoform 1 of Plectin-1 | Cytoplasm | 348 | 533462 | 11 | 2.8 |
| IPI00739539 | A5A3E0 | POTEF POTE ankyrin domain family member F | Cytoplasm | 266 | 123020 | 11 | 6.2 |
| IPI00014424 | Q05639 | EEF1A2 Elongation factor 1-alpha 2 | Cytoplasm | 114 | 50780 | 10 | 6.3 |
| IPI00418262 | P09972 | ALDOC Fructose-bisphosphate aldolase | Cytoplasm | 558 | 49062 | 9 | 22.4 |
| IPI00178352 | Q14315 | FLNC Isoform 1 of Filamin-C | Cytoplasm | 529 | 293407 | 9 | 4.8 |
| IPI00456969 | Q14204 | DYNC1H1 Cytoplasmic dynein 1 heavy chain 1 | Cytoplasm | 516 | 534809 | 9 | 3 |
| IPI00177728 | Q96KP4 | CNDP2 Isoform 1 of Cytosolic non-specific dipeptidase | Cytoplasm | 329 | 53187 | 9 | 21.1 |
| IPI00218130 | P11217 | PYGM Glycogen phosphorylase, muscle form | Cytoplasm | 264 | 97487 | 9 | 7.6 |
| IPI00008868 | P46821 | MAP1B Microtubule-associated protein 1B | Cytoplasm | 447 | 271651 | 8 | 5.1 |
| IPI00029111 | Q6DEN2 | DPYSL3 Collapsin response mediator protein 4 long variant | Cytoplasm | 365 | 74321 | 8 | 15.1 |
| IPI00169383 | P00558 | PGK1 Phosphoglycerate kinase 1 | Cytoplasm | 302 | 44985 | 7 | 14.4 |
| IPI00916111 | P40925 | MDH1 Malate dehydrogenase | Cytoplasm | 296 | 38916 | 7 | 17.9 |
| IPI00384444 | P02533 | KRT14 Keratin, type I cytoskeletal 14 | Cytoplasm | 260 | 51872 | 6 | 9.3 |
| IPI00019502 | P35579 | MYH9 Isoform 1 of Myosin-9 | Cytoplasm | 168 | 227646 | 6 | 3 |
| IPI00217966 | P00338 | LDHA Isoform 1 of L-lactate dehydrogenase A chain | Cytoplasm | 320 | 36950 | 5 | 16 |
| IPI00011200 | O43175 | PHGDH D-3-phosphoglycerate dehydrogenase | Cytoplasm | 278 | 57356 | 5 | 9.4 |
| IPI00022774 | P55072 | VCP Transitional endoplasmic reticulum ATPase | Cytoplasm | 267 | 89950 | 5 | 8.3 |
| IPI00302592 | Q60FE6 | FLNA Isoform 2 of Filamin-A | Cytoplasm | 207 | 282581 | 4 | 2.6 |
| IPI00747849 | P05026 | ATP1B1 Isoform 1 of Sodium/potassium-transporting ATPase subunit beta-1 | Cytoplasm | 161 | 35438 | 4 | 17.2 |
| IPI00219029 | P17174 | GOT1 Aspartate aminotransferase, cytoplasmic | Cytoplasm | 160 | 46447 | 4 | 9.9 |
| IPI00011932 | O43301 | HSPA12A Heat shock 70 kDa protein 12A | Cytoplasm | 140 | 75217 | 4 | 8.4 |
| IPI00479143 | O95613 | PCNT Pericentrin | Cytoplasm | 39 | 380644 | 4 | 0.6 |
| IPI00219585 | P08237 | PFKM Isoform 2 of 6-phosphofructokinase, muscle type | Cytoplasm | 250 | 82579 | 3 | 5.5 |
| IPI00027497 | P06744 | GPI Glucose-6-phosphate isomerase | Cytoplasm | 226 | 63335 | 3 | 7.7 |
| IPI00006451 | P46459 | NSF Vesicle-fusing ATPase | Cytoplasm | 197 | 83055 | 3 | 4.6 |
| IPI00024067 | Q00610 | CLTC Isoform 1 of Clathrin heavy chain 1 | Cytoplasm | 108 | 193260 | 3 | 3.6 |
| IPI00397809 | Q2UVF0 | KIF21B Kinesin-like protein KIF21B variant | Cytoplasm | 52 | 188037 | 3 | 0.9 |
| IPI00783950 | Q8WZ42 | TTN Isoform 6 of Titin | Cytoplasm | 44 | 634993 | 3 | 0.2 |
| IPI00016461 | P51178 | PLCD1 phospholipase C, delta 1 isoform 1 | Cytoplasm | 163 | 88878 | 2 | 5.3 |
| IPI00219526 | P36871 | PGM1 Isoform 1 of Phosphoglucomutase-1 | Cytoplasm | 144 | 61696 | 2 | 5 |
| IPI00027626 | P40227 | CCT6A T-complex protein 1 subunit zeta | Cytoplasm | 142 | 58444 | 2 | 6 |
| IPI00413641 | P15121 | AKR1B1 Aldose reductase | Cytoplasm | 120 | 36230 | 2 | 9.5 |
| IPI00335509 | Q9BPU6 | DPYSL5 Dihydropyrimidinase-related protein 5 | Cytoplasm | 115 | 61952 | 2 | 5.1 |
| IPI00008215 | P48163 | ME1 NADP-dependent malic enzyme | Cytoplasm | 104 | 64679 | 2 | 8.2 |
| IPI00219301 | P29966 | MARCKS Myristoylated alanine-rich C-kinase substrate | Cytoplasm | 103 | 31707 | 2 | 9.9 |
| IPI00005159 | P61160 | ACTR2 Actin-related protein 2 | Cytoplasm | 100 | 45017 | 2 | 9.1 |
| IPI00289334 | O75369 | FLNB Isoform 1 of Filamin-B | Cytoplasm | 94 | 280188 | 2 | 0.9 |
| IPI00015309 | Q99456 | KRT12 Keratin, type I cytoskeletal 12 | Cytoplasm | 82 | 53592 | 2 | 4 |
| IPI00848226 | P63244 | GNB2L1 Guanine nucleotide-binding protein subunit beta-2-like 1 | Cytoplasm | 77 | 35511 | 2 | 14.2 |
| IPI00021753 | Q9NQT8 | KIF13B Kinesin-like protein KIF13B | Cytoplasm | 43 | 203909 | 2 | 0.9 |
| IPI00013455 | P30622 | CLIP1 Isoform 1 of CAP-Gly domain-containing linker protein 1 | Cytoplasm | 42 | 162888 | 2 | 0.8 |
| IPI00419903 | A6ND91 | ASPDH Putative L-aspartate dehydrogenase | Cytoplasm | 42 | 30213 | 2 | 2.8 |
| IPI00216142 | P51530 | DNA2 DNA replication helicase 2 homolog | Cytoplasm | 42 | 131594 | 2 | 1.6 |
| IPI00944623 | Q08378 | GOLGA3 Isoform 3 of Golgin subfamily A member 3 | Cytoplasm | 41 | 126451 | 2 | 1.3 |
| IPI00307165 | Q96LD4 | TRIM47 Tripartite motif-containing protein 47 | Cytoplasm | 38 | 70968 | 2 | 3.1 |
| IPI00030578 | Q9HCJ6 | VAT1L Synaptic vesicle membrane protein VAT-1 homolog-like | Cytoplasm | 90 | 46212 | 1 | 5 |
| IPI00645078 | P22314 | UBA1 Ubiquitin-like modifier-activating enzyme 1 | Cytoplasm | 77 | 118858 | 1 | 2.2 |
| IPI00014177 | Q15019 | SEPT2 Isoform 1 of Septin-2 | Cytoplasm | 75 | 41689 | 1 | 3.3 |
| IPI00218914 | P00352 | ALDH1A1 Retinal dehydrogenase 1 | Cytoplasm | 74 | 55454 | 1 | 2.6 |
| IPI00008994 | Q9UN36 | NDRG2 Isoform 1 of Protein NDRG2 | Cytoplasm | 70 | 41114 | 1 | 5.9 |
| IPI00219525 | P52209 | PGD 6-phosphogluconate dehydrogenase, decarboxylating | Cytoplasm | 69 | 53619 | 1 | 4.8 |
| IPI00410714 | P69905 | HBA2;HBA1 Hemoglobin subunit alpha | Hb | 65 | 15305 | 1 | 10.6 |
| IPI00419237 | P28838 | LAP3 Isoform 1 of Cytosol aminopeptidase | Cytoplasm | 63 | 56530 | 1 | 2.3 |
| IPI00046057 | P61764 | STXBP1 Isoform 2 of Syntaxin-binding protein 1 | Cytoplasm | 62 | 69091 | 1 | 1.7 |
| IPI00022082 | Q92599 | SEPT8 Isoform 2 of Septin-8 | Cytoplasm | 59 | 50068 | 1 | 2.1 |
| IPI00026781 | P49327 | FASN Fatty acid synthase | Cytoplasm | 52 | 275877 | 1 | 1 |
| IPI00456623 | Q96GW7 | BCAN Isoform 1 of Brevican core protein | Cytoplasm | 52 | 100539 | 1 | 1.2 |
| IPI00289862 | Q12765 | SCRN1 Secernin-1 | Cytoplasm | 51 | 46980 | 1 | 2.7 |
| IPI00173346 | Q6PCE3 | PGM2L1 Glucose 1,6-bisphosphate synthase | Cytoplasm | 49 | 71380 | 1 | 1.8 |
| IPI00641181 | P49006 | MARCKSL1 MARCKS-related protein | Cytoplasm | 44 | 19574 | 1 | 6.7 |
| IPI00010466 | P05771 | PRKCB Isoform Beta-I of Protein kinase C beta type | Cytoplasm | 42 | 77960 | 1 | 1.3 |
| IPI00429191 | P62495 | ETF1 Eukaryotic peptide chain release factor subunit 1 | Cytoplasm | 39 | 49228 | 1 | 6.2 |
| IPI00216337 | Q9UJY5 | GGA1 Isoform 2 of ADP-ribosylation factor-binding protein GGA1 | Cytoplasm | 34 | 66692 | 1 | 2.1 |
| IPI00003865 | P11142 | HSPA8 Isoform 1 of Heat shock cognate 71 kDa protein | Nucleus | 1101 | 71082 | 27 | 33.7 |
| IPI00642259 | Q03001 | DST Dystonin | Nucleus | 62 | 862054 | 7 | 0.3 |
| IPI00026673 | P39880 | CUX1 Isoform 1 of Homeobox protein cut-like 1 | Nucleus | 64 | 164629 | 4 | 1.2 |
| IPI00173359 | Q9BZF9 | UACA Uveal autoantigen with coiled-coil domains and ankyrin repeats | Nucleus | 50 | 163545 | 3 | 0.9 |
| IPI00001639 | Q14974 | KPNB1 Importin subunit beta-1 | Nucleus | 163 | 98420 | 2 | 3.1 |
| IPI00221234 | P49419 | ALDH7A1 aldehyde dehydrogenase 7 family, member A1 | Nucleus | 129 | 59020 | 2 | 4.8 |
| IPI00023748 | Q13765 | NACA Nascent polypeptide-associated complex subunit alpha | Nucleus | 85 | 23370 | 2 | 6 |
| IPI00032958 | Q9NQW6 | ANLN Isoform 2 of Actin-binding protein anillin | Nucleus | 68 | 121253 | 2 | 2.7 |
| IPI00793443 | O00410 | IPO5 Isoform 1 of Importin-5 | Nucleus | 53 | 125032 | 2 | 1.8 |
| IPI00009286 | Q03164 | MLL Isoform 1 of Histone-lysine N-methyltransferase MLL | Nucleus | 49 | 436058 | 2 | 0.6 |
| IPI00291796 | Q8IY92 | BTBD12 Isoform 1 of Structure-specific endonuclease subunit SLX4 | Nucleus | 44 | 201713 | 2 | 1.5 |
| IPI00143753 | O15042 | SR140 Isoform 1 of U2-associated protein SR140 | Nucleus | 43 | 118675 | 2 | 2 |
| IPI00910646 | B4DDI4 | BHLHE40 cDNA FLJ58052, weakly similar to Class B basic helix-loop-helix protein 2 | Nucleus | 43 | 18135 | 2 | 11.6 |
| IPI00303832 | Q92541 | RTF1 Paf1/RNA polymerase II complex component | Nucleus | 42 | 80493 | 2 | 2.8 |
| IPI00001722 | P78549 | NTHL1 Endonuclease III-like protein 1 | Nucleus | 42 | 34767 | 2 | 6.1 |
| IPI00304621 | Q9C0D4 | ZNF518B Zinc finger protein 518B | Nucleus | 39 | 120939 | 2 | 1.6 |
| IPI00876962 | Q27J81 | INF2 Isoform 2 of Inverted formin-2 | Nucleus | 93 | 135788 | 1 | 1.1 |
| IPI00550069 | P13489 | RNH1 Ribonuclease inhibitor | Nucleus | 48 | 51766 | 1 | 7.2 |
| IPI00013421 | Q13491 | GPM6B Isoform 1 of Neuronal membrane glycoprotein M6-b | Nucleus | 46 | 29882 | 1 | 4.9 |
| IPI00645181 | Q5JVD2 | CEP110 Centrosomal protein 110kDa | Nucleus | 36 | 94017 | 1 | 1.1 |
| IPI00745872 | P02768 | ALB Isoform 1 of Serum albumin | Secreted | 1916 | 71317 | 85 | 60.9 |
| IPI00026314 | P06396 | GSN Isoform 1 of Gelsolin | Secreted | 993 | 86043 | 28 | 30.8 |
| IPI00160552 | Q92752 | TNR Isoform 1 of Tenascin-R | Secreted | 493 | 151791 | 6 | 8.3 |
| IPI00218725 | P24043 | LAMA2 laminin alpha 2 subunit isoform b precursor | Secreted | 109 | 352589 | 3 | 1.4 |
| IPI00297646 | P02452 | COL1A1 Collagen alpha-1(I) chain | Secreted | 81 | 139853 | 3 | 2.1 |
| IPI00021885 | P02671 | FGA Isoform 1 of Fibrinogen alpha chain | Secreted | 44 | 95656 | 3 | 2.5 |
| IPI00022200 | P12111 | COL6A3 Isoform 1 of Collagen alpha-3(VI) chain | Secreted | 161 | 345163 | 2 | 1.1 |
| IPI00304962 | P08123 | COL1A2 Collagen alpha-2(I) chain | Secreted | 122 | 129723 | 2 | 2.4 |
| IPI00020987 | P51888 | PRELP Prolargin | Secreted | 98 | 44181 | 2 | 5.8 |
| IPI00176193 | Q05707 | COL14A1 Isoform 1 of Collagen alpha-1(XIV) chain | Secreted | 84 | 194478 | 2 | 1.6 |
| IPI00260630 | Q76M96 | CCDC80 Isoform 1 of Coiled-coil domain-containing protein 80 | Secreted | 49 | 108505 | 2 | 1.9 |
| IPI00010790 | P21810 | BGN Biglycan | Secreted | 73 | 42027 | 1 | 3 |
| IPI00007047 | P05109 | S100A8 Protein S100-A8 | Secreted | 72 | 10885 | 1 | 22.6 |
| IPI00292836 | Q9P1Z9 | KIAA1529 Isoform 1 of Unknown protein KIAA1529 | Unknown | 63 | 192404 | 34 | 1.8 |
| IPI00643920 | P29401 | TKT cDNA FLJ54957, highly similar to Transketolase | Unknown | 418 | 69382 | 10 | 15.8 |
| IPI00031461 | P50395 | GDI2 cDNA FLJ60299, highly similar to Rab GDP dissociation inhibitor beta | Unknown | 473 | 51577 | 9 | 22 |
| IPI00220342 | O94760 | DDAH1 N(G),N(G)-dimethylarginine dimethylaminohydrolase 1 | Unknown | 272 | 31444 | 7 | 26.7 |
| IPI00942772 |  | TUBA4B Putative Unknown protein ENSP00000403894 | Unknown | 169 | 30377 | 7 | 12.8 |
| IPI00872463 |  | TUBAL3 50 kDa protein | Unknown | 163 | 50674 | 7 | 12.8 |
| IPI00738806 | C9J6C0 | MYO7B Putative Unknown protein MYO7B | Unknown | 53 | 243711 | 5 | 0.9 |
| IPI00745035 |  | - Putative Unknown protein ENSP00000339838 | Unknown | 67 | 15103 | 3 | 25.6 |
| IPI00384938 | Q7Z351 | IGHV4-31;LOC100290320;LOC100294459;IGHG1 Putative Unknown protein DKFZp686N02209 | Unknown | 131 | 53503 | 2 | 7.5 |
| IPI00033025 | E7EPK1 | SEPT7 51 kDa protein | Unknown | 122 | 50820 | 2 | 7.1 |
| IPI00025465 | P20774 | OGN cDNA FLJ59205, highly similar to Mimecan | Unknown | 98 | 40870 | 1 | 4.5 |
| IPI00424869 | Q6U7G8 | RAP1GDS1 RAP1, GTP-GDP dissociation stimulator 1 isoform 6 | Unknown | 90 | 57070 | 1 | 3.7 |
| IPI00383164 | Q8WY24 | IGHA1 SNC66 protein | Unknown | 55 | 54601 | 1 | 2 |
| IPI00179330 | P62979 | UBC;RPS27A;UBB ubiquitin and ribosomal protein S27a precursor | Unknown | 52 | 18296 | 1 | 10.3 |
| IPI00024638 |  | CKMT1B;CKMT1A Putative Unknown protein CKMT1A | Unknown | 50 | 16440 | 1 | 17.6 |
| IPI00168184 | B4DE69 | PPP2R1A cDNA FLJ34068 fis, clone FCBBF3001918, highly similar to SERINE/THREONINE PROTEIN PHOSPHATASE 2A, 65 kDa REGULATORY SUBUNIT A, ALPHA ISOFORM | Unknown | 41 | 57781 | 1 | 2 |

**Supplementary** **Table 3:** Proteins identified by LC-MSMS, sample WM2

| **Protein Accession Number** | **Uniprot Entry** | **Protein Description** | **Sub cellular location** | **Protein Score** | **Protein Mass** | **Protein matches** | **Protein coverage** |
| --- | --- | --- | --- | --- | --- | --- | --- |
| IPI00219661 | P60201 | PLP1 Isoform 1 of Myelin proteolipid protein | Myelin | 453 | 30855 | 96 | 29.2 |
| IPI00220993 | P09543 | CNP Isoform CNPI of 2~3~-cyclic-nucleotide 3~-phosphodiesterase | Myelin | 1322 | 45469 | 39 | 50.9 |
| IPI00026237 | P20916 | MAG Myelin-associated glycoprotein | Myelin | 276 | 69880 | 10 | 9.3 |
| IPI00556079 | Q16653 | MOG Isoform 1 of Myelin-oligodendrocyte glycoprotein | Myelin | 190 | 28560 | 3 | 13.8 |
| IPI00478921 | P25189 | MPZ myelin protein zero | Myelin | 85 | 28684 | 3 | 11.2 |
| IPI00465248 | P06733 | ENO1 Isoform alpha-enolase of Alpha-enolase | Membrane | 1294 | 47481 | 33 | 51.6 |
| IPI00292836 | Q9P1Z9 | KIAA1529 Isoform 1 of Unknown protein KIAA1529 | Membrane | 37 | 192404 | 33 | 1.2 |
| IPI00302840 | P13637 | ATP1A3 Sodium/potassium-transporting ATPase subunit alpha-3 | Membrane | 898 | 113102 | 21 | 17.8 |
| IPI00024067 | Q00610 | CLTC Isoform 1 of Clathrin heavy chain 1 | Membrane | 980 | 193260 | 20 | 14.9 |
| IPI00003021 | P50993 | ATP1A2 Sodium/potassium-transporting ATPase subunit alpha-2 | Membrane | 856 | 113505 | 20 | 16 |
| IPI00303476 | P06576 | ATP5B ATP synthase subunit beta mitochondrial | Membrane | 683 | 56525 | 17 | 38.2 |
| IPI00216171 | P09104 | ENO2 Gamma-enolase | Membrane | 799 | 47581 | 14 | 37.1 |
| IPI00220737 | P13591 | NCAM1 Isoform 3 of Neural cell adhesion molecule 1 | Membrane | 556 | 84345 | 11 | 15.8 |
| IPI00219365 | P26038 | MSN Moesin | Membrane | 341 | 67892 | 11 | 12.7 |
| IPI00440493 | P25705 | ATP5A1 ATP synthase subunit alpha mitochondrial | Membrane | 487 | 59828 | 7 | 16.5 |
| IPI00024853 | Q9BXM0 | PRX Isoform 1 of Periaxin | Membrane | 179 | 155248 | 6 | 3.1 |
| IPI00747849 | P05026 | ATP1B1 Isoform 1 of Sodium/potassium-transporting ATPase subunit beta-1 | Membrane | 252 | 35438 | 5 | 21.8 |
| IPI00017367 | P35241 | RDX Radixin isoform CRA_a | Membrane | 199 | 71176 | 5 | 8.9 |
| IPI00007682 | P38606 | ATP6V1A V-type proton ATPase catalytic subunit A | Membrane | 150 | 68660 | 5 | 4.2 |
| IPI00219301 | P29966 | MARCKS Myristoylated alanine-rich C-kinase substrate | Membrane | 176 | 31707 | 4 | 20.5 |
| IPI00008274 | Q01518 | CAP1 Isoform 1 of Adenylyl cyclase-associated protein 1 | Membrane | 138 | 52222 | 4 | 8.6 |
| IPI00178352 | Q14315 | FLNC Isoform 1 of Filamin-C | Membrane | 127 | 293407 | 4 | 2.2 |
| IPI00022799 | P55087 | AQP4 Isoform 2 of Aquaporin-4 | Membrane | 106 | 35263 | 4 | 9.9 |
| IPI00046057 | P61764 | STXBP1 Isoform 2 of Syntaxin-binding protein 1 | Membrane | 232 | 69091 | 3 | 6 |
| IPI00018206 | P00505 | GOT2 Aspartate aminotransferase mitochondrial | Membrane | 193 | 47844 | 3 | 10.9 |
| IPI00291006 | P40926 | MDH2 Malate dehydrogenase mitochondrial | Membrane | 188 | 35937 | 3 | 10.9 |
| IPI00031522 | P40939 | HADHA Trifunctional enzyme subunit alpha mitochondrial | Membrane | 160 | 83688 | 3 | 4.2 |
| IPI00009960 | Q16891 | IMMT Isoform 1 of Mitochondrial inner membrane protein | Membrane | 107 | 84026 | 3 | 4.4 |
| IPI00029751 | Q12860 | CNTN1 Isoform 1 of Contactin-1 | Membrane | 53 | 114104 | 3 | 1.1 |
| IPI00470809 | Q68CR1 | SEL1L3 Isoform 1 of Protein sel-1 homolog 3 | Membrane | 49 | 129512 | 3 | 1.4 |
| IPI00300020 | P43004 | SLC1A2 Isoform 1 of Excitatory amino acid transporter 2 | Membrane | 47 | 62577 | 3 | 2.6 |
| IPI00015872 | P19075 | TSPAN8 Tetraspanin-8 | Membrane | 136 | 26711 | 2 | 10.1 |
| IPI00658109 | P12532 | CKMT1B;CKMT1A Isoform 1 of Creatine kinase U-type mitochondrial | Membrane | 114 | 47406 | 2 | 11.3 |
| IPI00009532 | P80404 | ABAT cDNA FLJ56034 highly similar to 4-aminobutyrate aminotransferase mitochondrial | Membrane | 98 | 58876 | 2 | 5.2 |
| IPI00289758 | P17655 | CAPN2 Calpain-2 catalytic subunit | Membrane | 92 | 80814 | 2 | 2.1 |
| IPI00007812 | P21281 | ATP6V1B2 V-type proton ATPase subunit B brain isoform | Membrane | 90 | 56807 | 2 | 4.5 |
| IPI00026053 | O75508 | CLDN11 Claudin-11 | Membrane | 82 | 22720 | 2 | 8.2 |
| IPI00641181 | P49006 | MARCKSL1 MARCKS-related protein | Membrane | 60 | 19574 | 2 | 6.7 |
| IPI00215997 | P21926 | CD9 CD9 antigen | Membrane | 51 | 25969 | 2 | 11 |
| IPI00020210 | O75923 | DYSF Isoform 14 of Dysferlin | Membrane | 50 | 239397 | 2 | 0.6 |
| IPI00305383 | P22695 | UQCRC2 Cytochrome b-c1 complex subunit 2 mitochondrial | Membrane | 43 | 48584 | 2 | 4.4 |
| IPI00016461 | P51178 | PLCD1 phospholipase C delta 1 isoform 1 | Membrane | 81 | 88878 | 1 | 1.9 |
| IPI00018931 | Q96QK1 | VPS35 Vacuolar protein sorting-associated protein 35 | Membrane | 80 | 92447 | 1 | 1.4 |
| IPI00293971 | P14415 | ATP1B2 Sodium/potassium-transporting ATPase subunit beta-2 | Membrane | 78 | 33745 | 1 | 4.1 |
| IPI00157414 | Q6UWR7 | ENPP6 Ectonucleotide pyrophosphatase/phosphodiesterase family member 6 | Membrane | 65 | 50551 | 1 | 4.3 |
| IPI00293074 | Q8IWA5 | SLC44A2 Isoform 2 of Choline transporter-like protein 2 | Membrane | 59 | 82003 | 1 | 1.7 |
| IPI00294578 | P21980 | TGM2 Isoform 1 of Protein-glutamine gamma-glutamyltransferase 2 | Membrane | 56 | 78420 | 1 | 2.3 |
| IPI00298289 | Q9NQC3 | RTN4 Isoform 2 of Reticulon-4 | Membrane | 52 | 40350 | 1 | 3.8 |
| IPI00328156 | P27338 | MAOB Amine oxidase [flavin-containing] B | Membrane | 48 | 59238 | 1 | 2.1 |
| IPI00220002 | O75781 | PALM Isoform 2 of Paralemmin | Membrane | 42 | 37306 | 1 | 6.4 |
| IPI00297160 | P16070 | CD44 Isoform 12 of CD44 antigen | Membrane | 41 | 39904 | 1 | 2.2 |
| IPI00015473 | P43003 | SLC1A3 Excitatory amino acid transporter 1 | Membrane | 41 | 59705 | 1 | 3.7 |
| IPI00019171 | Q99962 | SH3GL2 Endophilin-A1 | Membrane | 39 | 40108 | 1 | 2 |
| IPI00017480 | Q15465 | SHH Sonic hedgehog protein precursor | Membrane | 38 | 50293 | 1 | 2.2 |
| IPI00176903 | Q6NZI2 | PTRF Isoform 1 of Polymerase I and transcript release factor | Membrane | 36 | 43450 | 1 | 2.8 |
| IPI00219299 | Q9Y4G6 | TLN2 Talin-2 | Membrane | 36 | 273781 | 1 | 0.3 |
|  |  |  |  |  |  |  |  |
| IPI00025363 | P14136 | GFAP Isoform 1 of Glial fibrillary acidic protein | Cytoplasm | 2441 | 49907 | 427 | 68.3 |
| IPI00237671 | P07196 | NEFL Neurofilament light polypeptide | Cytoplasm | 1836 | 61536 | 128 | 59.5 |
| IPI00853115 | A5YM63 | NEFM NEFM protein | Cytoplasm | 1633 | 98383 | 111 | 39.1 |
| IPI00792677 | B4DDU2 | TUBA1B cDNA FLJ60097 highly similar to Tubulin alpha-ubiquitous chain | Cytoplasm | 1119 | 46797 | 78 | 52.9 |
| IPI00180675 | Q71U36 | TUBA1A Tubulin alpha-1A chain | Cytoplasm | 1102 | 50788 | 78 | 49 |
| IPI00910602 | P12036 | NEFH Isoform 1 of Neurofilament heavy polypeptide | Cytoplasm | 1184 | 112639 | 72 | 24.2 |
| IPI00218343 | Q9BQE3 | TUBA1C Tubulin alpha-1C chain | Cytoplasm | 900 | 50548 | 69 | 42.3 |
| IPI00871535 | Q13813 | SPTAN1 Isoform 2 of Spectrin alpha chain brain | Cytoplasm | 2334 | 285717 | 64 | 22.2 |
| IPI00031370 | Q9BVA1 | TUBB2B Tubulin beta-2B chain | Cytoplasm | 1350 | 50377 | 62 | 56.2 |
| IPI00013475 | Q13885 | TUBB2A Tubulin beta-2A chain | Cytoplasm | 1395 | 50274 | 60 | 56.2 |
| IPI00007750 | P68366 | TUBA4A Tubulin alpha-4A chain | Cytoplasm | 1054 | 50634 | 59 | 47.1 |
| IPI00007752 | P68371 | TUBB2C Tubulin beta-2C chain | Cytoplasm | 1371 | 50255 | 57 | 56.2 |
| IPI00021439 | P60709 | ACTB Actin cytoplasmic 1 | Cytoplasm | 1198 | 42052 | 53 | 60.3 |
| IPI00909140 | P07436 | TUBB Tubulin beta chain | Cytoplasm | 1201 | 50095 | 51 | 49.1 |
| IPI00023598 | P04350 | TUBB4 Tubulin beta-4 chain | Cytoplasm | 1174 | 50010 | 50 | 52.3 |
| IPI00257508 | Q16555 | DPYSL2 Dihydropyrimidinase-related protein 2 | Cytoplasm | 1330 | 62711 | 46 | 55.8 |
| IPI00382470 | P07900 | HSP90AA1 Isoform 2 of Heat shock protein HSP 90-alpha | Cytoplasm | 1159 | 98670 | 44 | 29.9 |
| IPI00013683 | Q13509 | TUBB3 Tubulin beta-3 chain | Cytoplasm | 1049 | 50856 | 43 | 42.4 |
| IPI00418471 | P08670 | VIM Vimentin | Cytoplasm | 957 | 53676 | 41 | 40.1 |
| IPI00219018 | P04406 | GAPDH Glyceraldehyde-3-phosphate dehydrogenase | Cytoplasm | 1023 | 36201 | 35 | 62.4 |
| IPI00414676 | P08238 | HSP90AB1 Heat shock protein HSP 90-beta | Cytoplasm | 918 | 83554 | 35 | 27.3 |
| IPI00005614 | Q01082 | SPTBN1 Isoform Long of Spectrin beta chain brain 1 | Cytoplasm | 1360 | 275237 | 31 | 10.7 |
| IPI00514530 | A6NL76 | ACTA1 Putative Unknown protein ACTA1 | Cytoplasm | 521 | 32596 | 31 | 38.4 |
| IPI00008603 | P62736 | ACTA2 Actin aortic smooth muscle | Cytoplasm | 540 | 42381 | 29 | 29.2 |
| IPI00641706 | Q2NKY5 | TUBB6 46 kDa protein | Cytoplasm | 512 | 46248 | 29 | 23.5 |
| IPI00022977 | P12277 | CKB Creatine kinase B-type | Cytoplasm | 647 | 42902 | 27 | 31.5 |
| IPI00954527 | Q13707 | ACTA2 ACTA2 protein (Fragment) | Cytoplasm | 451 | 37125 | 24 | 30.9 |
| IPI00003269 | Q562R1 | ACTBL2 Beta-actin-like protein 2 | Cytoplasm | 369 | 42318 | 24 | 17.8 |
| IPI00026314 | P06396 | GSN Isoform 1 of Gelsolin | Cytoplasm | 914 | 86043 | 22 | 33.6 |
| IPI00017855 | Q99798 | ACO2 Aconitate hydratase mitochondrial | Cytoplasm | 916 | 86113 | 21 | 27.3 |
| IPI00219217 | P07195 | LDHB L-lactate dehydrogenase B chain | Cytoplasm | 807 | 36900 | 18 | 44 |
| IPI00004358 | P11216 | PYGB Glycogen phosphorylase brain form | Cytoplasm | 576 | 97319 | 15 | 16.8 |
| IPI00739539 | A5A3E0 | POTEF POTE ankyrin domain family member F | Cytoplasm | 263 | 123020 | 15 | 5.9 |
| IPI00010154 | P31150 | GDI1 Rab GDP dissociation inhibitor alpha | Cytoplasm | 749 | 51177 | 14 | 36.7 |
| IPI00304925 | P08107 | HSPA1A;HSPA1B Heat shock 70 kDa protein 1A/1B | Cytoplasm | 556 | 70294 | 14 | 18.7 |
| IPI00643920 | P29401 | TKT cDNA FLJ54957 highly similar to Transketolase | Cytoplasm | 517 | 69382 | 13 | 20.1 |
| IPI00418262 | P09972 | ALDOC Fructose-bisphosphate aldolase | Cytoplasm | 512 | 49062 | 11 | 23.1 |
| IPI00007702 | P54652 | HSPA2 Heat shock-related 70 kDa protein 2 | Cytoplasm | 438 | 70263 | 11 | 13.6 |
| IPI00915869 | B9A041 | MDH1 Malate dehydrogenase | Cytoplasm | 425 | 23195 | 11 | 44.3 |
| IPI00218130 | P11217 | PYGM Glycogen phosphorylase muscle form | Cytoplasm | 387 | 97487 | 10 | 12.7 |
| IPI00217966 | P00338 | LDHA Isoform 1 of L-lactate dehydrogenase A chain | Cytoplasm | 451 | 36950 | 9 | 26.5 |
| IPI00465439 | P04075 | ALDOA Fructose-bisphosphate aldolase A | Cytoplasm | 424 | 39851 | 9 | 31.9 |
| IPI00169383 | P00558 | PGK1 Phosphoglycerate kinase 1 | Cytoplasm | 442 | 44985 | 8 | 24.5 |
| IPI00221226 | P08133 | ANXA6 Annexin A6 | Cytoplasm | 426 | 76168 | 8 | 15.6 |
| IPI00008868 | P46821 | MAP1B Microtubule-associated protein 1B | Cytoplasm | 243 | 271651 | 8 | 2.6 |
| IPI00398002 | Q15149 | PLEC1 Isoform 3 of Plectin-1 | Cytoplasm | 228 | 519655 | 8 | 1.5 |
| IPI00398625 | Q86YZ3 | HRNR Hornerin | Cytoplasm | 119 | 283140 | 8 | 1.1 |
| IPI00003362 | P11021 | HSPA5 HSPA5 protein | Cytoplasm | 400 | 72492 | 7 | 11.8 |
| IPI00784154 | P10809 | HSPD1 60 kDa heat shock protein mitochondrial | Cytoplasm | 255 | 61187 | 7 | 11.7 |
| IPI00029111 | Q14195 | DPYSL3 Collapsin response mediator protein 4 long variant | Cytoplasm | 245 | 74321 | 7 | 6.7 |
| IPI00456969 | Q14204 | DYNC1H1 Cytoplasmic dynein 1 heavy chain 1 | Cytoplasm | 282 | 534809 | 6 | 1.7 |
| IPI00177728 | Q96KP4 | CNDP2 Isoform 1 of Cytosolic non-specific dipeptidase | Cytoplasm | 316 | 53187 | 5 | 12.4 |
| IPI00020984 | B4DGP8 | CANX cDNA FLJ55574 highly similar to Calnexin | Cytoplasm | 314 | 71971 | 5 | 9.9 |
| IPI00027230 | P14625 | HSP90B1 Endoplasmin | Cytoplasm | 287 | 92696 | 5 | 5.6 |
| IPI00013508 | P12814 | ACTN1 Alpha-actinin-1 | Cytoplasm | 233 | 103563 | 5 | 4 |
| IPI00008994 | Q9UN36 | NDRG2 Isoform 1 of Protein NDRG2 | Cytoplasm | 210 | 41114 | 5 | 19.9 |
| IPI00022465 | O14578 | CIT Isoform 1 of Citron Rho-interacting kinase | Cytoplasm | 85 | 233339 | 5 | 1.1 |
| IPI00879751 |  | - 44 kDa protein | Cytoplasm | 45 | 44886 | 5 | 4.6 |
| IPI00645078 | P22314 | UBA1 Ubiquitin-like modifier-activating enzyme 1 | Cytoplasm | 248 | 118858 | 4 | 6.3 |
| IPI00219525 | P52209 | PGD 6-phosphogluconate dehydrogenase decarboxylating | Cytoplasm | 204 | 53619 | 4 | 12.8 |
| IPI00294187 | Q9Y2J8 | PADI2 Protein-arginine deiminase type-2 | Cytoplasm | 135 | 76257 | 4 | 5.7 |
| IPI00419922 | Q6IPM2 | IQCE Isoform 1 of IQ domain-containing protein E | Cytoplasm | 73 | 77649 | 4 | 3.9 |
| IPI00293251 |  | DST Isoform 6 of Bullous pemphigoid antigen 1 isoforms 6/9/10 | Cytoplasm | 42 | 593763 | 4 | 0.3 |
| IPI00011932 | O43301 | HSPA12A Heat shock 70 kDa protein 12A | Cytoplasm | 151 | 75217 | 3 | 8.6 |
| IPI00003348 | P62879 | GNB2 Guanine nucleotide-binding protein G(I)/G(S)/G(T) subunit beta-2 | Cytoplasm | 117 | 38048 | 3 | 5.9 |
| IPI00014177 | Q15019 | SEPT2 Isoform 1 of Septin-2 | Cytoplasm | 116 | 41689 | 3 | 5.5 |
| IPI00219029 | P17174 | GOT1 Aspartate aminotransferase cytoplasmic | Cytoplasm | 102 | 46447 | 3 | 4.8 |
| IPI00219585 | P08237 | PFKM Isoform 2 of 6-phosphofructokinase muscle type | Cytoplasm | 100 | 82579 | 3 | 2.8 |
| IPI00019223 | Q99996 | AKAP9 Isoform 1 of A-kinase anchor protein 9 | Cytoplasm | 53 | 455725 | 3 | 0.4 |
| IPI00011200 | O43175 | PHGDH D-3-phosphoglycerate dehydrogenase | Cytoplasm | 171 | 57356 | 2 | 4.9 |
| IPI00179109 | Q8IXJ6 | SIRT2 Isoform 1 of NAD-dependent deacetylase sirtuin-2 | Cytoplasm | 157 | 43782 | 2 | 6.2 |
| IPI00002966 | P34932 | HSPA4 Heat shock 70 kDa protein 4 | Cytoplasm | 143 | 95127 | 2 | 3.5 |
| IPI00221332 | Q9UQ16 | DNM3 Isoform 1 of Dynamin-3 | Cytoplasm | 89 | 98084 | 2 | 2.8 |
| IPI00010133 | P31146 | CORO1A Coronin-1A | Cytoplasm | 85 | 51678 | 2 | 5.9 |
| IPI00654755 | P68871 | HBB Hemoglobin subunit beta | Cytoplasm | 80 | 16102 | 2 | 8.8 |
| IPI00009790 | Q01813 | PFKP 6-phosphofructokinase type C | Cytoplasm | 68 | 86454 | 2 | 2.2 |
| IPI00930224 | Q5TCS8 | AKD1 adenylate kinase domain containing 1 isoform 1 | Cytoplasm | 54 | 222698 | 2 | 0.7 |
| IPI00394818 | Q6NY19 | KANK3 Isoform 1 of KN motif and ankyrin repeat domain-containing protein 3 | Cytoplasm | 52 | 88999 | 2 | 2.4 |
| IPI00220342 | O94760 | DDAH1 N(G)N(G)-dimethylarginine dimethylaminohydrolase 1 | Cytoplasm | 51 | 31444 | 2 | 3.5 |
| IPI00165984 | Q9P2L0 | WDR35 Isoform 1 of WD repeat-containing protein 35 | Cytoplasm | 46 | 135229 | 2 | 1.6 |
| IPI00294653 | Q9Y3R5 | DOPEY2 Isoform 1 of Protein dopey-2 | Cytoplasm | 46 | 260245 | 2 | 1 |
| IPI00015973 | O43491 | EPB41L2 Band 4.1-like protein 2 | Cytoplasm | 36 | 113032 | 2 | 2 |
| IPI00291175 | P18206 | VCL Isoform 1 of Vinculin | Cytoplasm | 105 | 117220 | 1 | 1.8 |
| IPI00006663 | P05091 | ALDH2 Aldehyde dehydrogenase mitochondrial | Cytoplasm | 93 | 56859 | 1 | 2.3 |
| IPI00424869 | Q6U7G8 | RAP1GDS1 RAP1 GTP-GDP dissociation stimulator 1 isoform 6 | Cytoplasm | 65 | 57070 | 1 | 3.7 |
| IPI00025252 | P30101 | PDIA3 Protein disulfide-isomerase A3 | Cytoplasm | 65 | 57146 | 1 | 2.2 |
| IPI00008215 | P48163 | ME1 NADP-dependent malic enzyme | Cytoplasm | 63 | 64679 | 1 | 3.7 |
| IPI00299402 | P11498 | PC Pyruvate carboxylase mitochondrial | Cytoplasm | 62 | 130293 | 1 | 1.2 |
| IPI00419237 | P28838 | LAP3 Isoform 1 of Cytosol aminopeptidase | Cytoplasm | 60 | 56530 | 1 | 2.3 |
| IPI00218914 | P00352 | ALDH1A1 Retinal dehydrogenase 1 | Cytoplasm | 58 | 55454 | 1 | 2.6 |
| IPI00289862 | Q12765 | SCRN1 Secernin-1 | Cytoplasm | 52 | 46980 | 1 | 3.9 |
| IPI00744692 | P37837 | TALDO1 Transaldolase | Cytoplasm | 49 | 37688 | 1 | 3 |
| IPI00746777 | P11766 | ADH5P4;ADH5 Alcohol dehydrogenase class-3 | Cytoplasm | 44 | 40554 | 1 | 2.1 |
| IPI00037448 | Q9UBQ7 | GRHPR Glyoxylate reductase/hydroxypyruvate reductase | Cytoplasm | 42 | 36045 | 1 | 7.6 |
| IPI00018465 | Q99832 | CCT7 T-complex protein 1 subunit eta | Cytoplasm | 38 | 59842 | 1 | 1.7 |
| IPI00419903 | A6ND91 | ASPDH Putative L-aspartate dehydrogenase | Cytoplasm | 38 | 30213 | 1 | 2.8 |
| IPI00479877 | P49189 | ALDH9A1 aldehyde dehydrogenase 9A1 | Cytoplasm | 36 | 57168 | 1 | 2.1 |
| IPI00550069 | P13489 | RNH1 Ribonuclease inhibitor | Cytoplasm | 35 | 51766 | 1 | 4.8 |
| IPI00385055 | P26232 | CTNNA2 Isoform 1 of Catenin alpha-2 | Cytoplasm | 34 | 106132 | 1 | 0.8 |
| IPI00745872 | P02768 | ALB Isoform 1 of Serum albumin | Secreted | 1876 | 71317 | 90 | 65.8 |
| IPI00418169 | P07355 | ANXA2 Isoform 2 of Annexin A2 | Secreted | 467 | 40671 | 9 | 30.5 |
| IPI00297646 | P02452 | COL1A1 Collagen alpha-1(I) chain | Secreted | 249 | 139853 | 9 | 3.5 |
| IPI00009802 | P13611 | VCAN Isoform V0 of Versican core protein | Secreted | 235 | 374585 | 8 | 1.7 |
| IPI00022463 | P02787 | TF Serotransferrin | Secreted | 306 | 79280 | 7 | 9.3 |
| IPI00020987 | P51888 | PRELP Prolargin | Secreted | 227 | 44181 | 6 | 14.1 |
| IPI00022200 | P12111 | COL6A3 Isoform 1 of Collagen alpha-3(VI) chain | Secreted | 210 | 345163 | 6 | 2.3 |
| IPI00553177 | P01009 | SERPINA1 Isoform 1 of Alpha-1-antitrypsin | Secreted | 140 | 46878 | 5 | 8.1 |
| IPI00304962 | P08123 | COL1A2 Collagen alpha-2(I) chain | Secreted | 165 | 129723 | 4 | 3.1 |
| IPI00010790 | P21810 | BGN Biglycan | Secreted | 85 | 42027 | 4 | 3 |
| IPI00550991 | P01011 | SERPINA3 cDNA FLJ35730 fis clone TESTI2003131 highly similar to ALPHA-1-ANTICHYMOTRYPSIN | Secreted | 212 | 50737 | 3 | 10.5 |
| IPI00298497 | P02675 | FGB Fibrinogen beta chain | Secreted | 107 | 56577 | 3 | 9.4 |
| IPI00025465 | P20774 | OGN cDNA FLJ59205 highly similar to Mimecan | Secreted | 65 | 40870 | 2 | 4.5 |
| IPI00020599 | P27797 | CALR Calreticulin | Secreted | 44 | 48283 | 2 | 2.2 |
| IPI00022429 | P02763 | ORM1 Alpha-1-acid glycoprotein 1 | Secreted | 69 | 23725 | 1 | 7 |
| IPI00783987 | P01024 | C3 Complement C3 (Fragment) | Secreted | 68 | 188569 | 1 | 1.3 |
| IPI00022082 | Q92599 | SEPT8 Isoform 2 of Septin-8 | Secreted | 61 | 50068 | 1 | 2.1 |
| IPI00032258 | P0C0L4 | C4A Complement C4-A | Secreted | 49 | 194247 | 1 | 1.5 |
| IPI00555812 | P02774 | GC Isoform 1 of Vitamin D-binding protein | Secreted | 45 | 54526 | 1 | 4.6 |
| IPI00291136 | P12109 | COL6A1 Collagen alpha-1(VI) chain | Secreted | 35 | 109602 | 1 | 2.3 |
| IPI00012119 | P07585 | DCN Isoform A of Decorin | Secreted | 34 | 40064 | 1 | 3.1 |
| IPI00479186 | P14618 | PKM2 Isoform M2 of Pyruvate kinase isozymes M1/M2 | Nucleus | 1012 | 58470 | 28 | 39 |
| IPI00220644 | P14618 | PKM2 Isoform M1 of Pyruvate kinase isozymes M1/M2 | Nucleus | 860 | 58538 | 25 | 35.4 |
| IPI00003865 | P11142 | HSPA8 Isoform 1 of Heat shock cognate 71 kDa protein | Nucleus | 951 | 71082 | 22 | 30.7 |
| IPI00022774 | P55072 | VCP Transitional endoplasmic reticulum ATPase | Nucleus | 910 | 89950 | 20 | 26.1 |
| IPI00645255 | Q5SX87 | GDI2 GDP dissociation inhibitor 2 | Nucleus | 278 | 29951 | 8 | 25.1 |
| IPI00007765 | P38646 | HSPA9 Stress-70 protein mitochondrial | Nucleus | 263 | 73920 | 4 | 7.1 |
| IPI00952748 | Q8WZ42 | TTN Isoform 5 of Titin | Nucleus | 73 | 3680117 | 4 | 0.1 |
| IPI00005264 | Q99959 |  | Nucleus | 49 | 97852 | 4 | 2 |
| IPI00853074 | Q8TD31 | CCHCR1 Coiled-coil alpha-helical rod protein 1 | Nucleus | 47 | 36497 | 4 | 6.6 |
| IPI00026216 | P55786 | NPEPPS Puromycin-sensitive aminopeptidase | Nucleus | 150 | 103895 | 3 | 2.6 |
| IPI00001639 | Q14974 | KPNB1 Importin subunit beta-1 | Nucleus | 149 | 98420 | 3 | 3.1 |
| IPI00221234 | P49419 | ALDH7A1 aldehyde dehydrogenase 7 family member A1 | Nucleus | 83 | 59020 | 3 | 3.7 |
| IPI00856045 | Q8IVF2 | AHNAK2 Isoform 1 of Protein AHNAK2 | Nucleus | 69 | 617383 | 3 | 0.5 |
| IPI00003494 | O95997 | PTTG1 Securin | Nucleus | 46 | 22125 | 3 | 7.9 |
| IPI00025447 | P68104 | EEF1A1 Elongation factor 1-alpha | Nucleus | 64 | 48181 | 2 | 2.9 |
| IPI00186808 | Q13625 | TP53BP2 Isoform 1 of Apoptosis-stimulating of p53 protein 2 | Nucleus | 53 | 126222 | 2 | 1.6 |
| IPI00179330 | P62979 | UBC;RPS27A;UBB ubiquitin and ribosomal protein S27a precursor | Nucleus | 51 | 18296 | 2 | 10.3 |
| IPI00644680 | Q96JG9 | ZNF469 Zinc finger protein 469 | Nucleus | 49 | 414169 | 2 | 0.4 |
| IPI00400922 | Q14690 | PDCD11 Protein RRP5 homolog | Nucleus | 47 | 209939 | 2 | 1 |
| IPI00014575 | Q99741 | CDC6 Cell division control protein 6 homolog | Nucleus | 46 | 63650 | 2 | 3.6 |
| IPI00003519 | Q15029 | EFTUD2 116 kDa U5 small nuclear ribonucleoprotein component | Nucleus | 45 | 110336 | 2 | 1.7 |
| IPI00646294 | Q6P087 | RPUSD3 RNA pseudouridylate synthase domain containing 3 isoform 1 | Nucleus | 43 | 38779 | 2 | 4.8 |
| IPI00409658 | O95935 | TBX18 T-box transcription factor TBX18 | Nucleus | 42 | 65225 | 2 | 3.8 |
| IPI00431645 | Q6NSB4 | HP HP protein | Nucleus | 37 | 31647 | 1 | 3.6 |
| IPI00384938 | Q7Z351 | IGHV4-31;LOC100290320;LOC100294459;IGHG1 Putative Unknown protein DKFZp686N02209 | Unknown | 193 | 53503 | 5 | 12.7 |
| IPI00942772 |  | TUBA4B Putative Unknown protein ENSP00000403894 | Unknown | 78 | 30377 | 4 | 7.5 |
| IPI00927152 |  | FSIP2 Putative Unknown protein FSIP2 | Unknown | 74 | 13990 | 4 | 4.8 |
| IPI00879430 |  | NCOR2 Putative Unknown protein NCOR2 | Unknown | 49 | 13777 | 2 | 7.6 |

**Supplementary** **Table 4:** Proteins identified by LC-MSMS, sample MS1

| **Protein Accession Number** | **Uniprot Entry** | **Protein Description** | **Sub cellular location** | **Protein Score** | **Protein Mass** | **Protein matches** | **Protein coverage** |
| --- | --- | --- | --- | --- | --- | --- | --- |
| IPI00478921 | P25189 | Tax_Id=9606 Gene_Symbol=MPZ myelin protein zero | Myelin | 483 | 28684 | 39 | 38.8 |
| IPI00219661 | P60201 | Tax_Id=9606 Gene_Symbol=PLP1 Isoform 1 of Myelin proteolipid protein | Myelin | 209 | 30855 | 5 | 9.4 |
| IPI00216171 | P09104 | Tax_Id=9606 Gene_Symbol=ENO2 Gamma-enolase | Membrane | 1073 | 47581 | 22 | 35.9 |
| IPI00303476 | P06576 | Tax_Id=9606 Gene_Symbol=ATP5B ATP synthase subunit beta, mitochondrial | Membrane | 910 | 56525 | 22 | 42.2 |
| IPI00178352 | Q14315 | Tax_Id=9606 Gene_Symbol=FLNC Isoform 1 of Filamin-C | Membrane | 526 | 293407 | 15 | 7.4 |
| IPI00384938 | Q7Z351 | Tax_Id=9606 Gene_Symbol=IGHV4-31;LOC100290320;LOC100294459;IGHG1 Putative Unknown protein DKFZp686N02209 | Membrane | 308 | 53503 | 13 | 20.5 |
| IPI00440493 | P25705 | Tax_Id=9606 Gene_Symbol=ATP5A1 ATP synthase subunit alpha, mitochondrial | Membrane | 439 | 59828 | 8 | 15.9 |
| IPI00031461 | P50395 | Tax_Id=9606 Gene_Symbol=GDI2 cDNA FLJ60299, highly similar to Rab GDP dissociation inhibitor beta | Membrane | 293 | 51577 | 7 | 19.2 |
| IPI00024067 | Q00610 | Tax_Id=9606 Gene_Symbol=CLTC Isoform 1 of Clathrin heavy chain 1 | Membrane | 177 | 193260 | 6 | 4.8 |
| IPI00220737 | P13591 | Tax_Id=9606 Gene_Symbol=NCAM1 Isoform 3 of Neural cell adhesion molecule 1 | Membrane | 137 | 84345 | 6 | 4.5 |
| IPI00219365 | P26038 | Tax_Id=9606 Gene_Symbol=MSN Moesin | Membrane | 61 | 67892 | 5 | 2.9 |
| IPI00029751 | Q12860 | Tax_Id=9606 Gene_Symbol=CNTN1 Isoform 1 of Contactin-1 | Membrane | 236 | 114104 | 4 | 4.7 |
| IPI00008274 | Q01518 | Tax_Id=9606 Gene_Symbol=CAP1 Isoform 1 of Adenylyl cyclase-associated protein 1 | Membrane | 149 | 52222 | 4 | 3.8 |
| IPI00215997 | P21926 | Tax_Id=9606 Gene_Symbol=CD9 CD9 antigen | Membrane | 120 | 25969 | 4 | 18.4 |
| IPI00016801 | P00367 | Tax_Id=9606 Gene_Symbol=GLUD1 Glutamate dehydrogenase 1, mitochondrial | Membrane | 107 | 61701 | 4 | 5 |
| IPI00302840 | P13637 | Tax_Id=9606 Gene_Symbol=ATP1A3 Sodium/potassium-transporting ATPase subunit alpha-3 | Membrane | 141 | 113102 | 3 | 4.8 |
| IPI00006663 | P05091 | Tax_Id=9606 Gene_Symbol=ALDH2 Aldehyde dehydrogenase, mitochondrial | Membrane | 94 | 56859 | 3 | 4.1 |
| IPI00022793 | P55084 | Tax_Id=9606 Gene_Symbol=HADHB Trifunctional enzyme subunit beta, mitochondrial | Membrane | 75 | 51547 | 3 | 7.2 |
| IPI00843975 | P15311 | Tax_Id=9606 Gene_Symbol=EZR Ezrin | Membrane | 37 | 69484 | 3 | 5.8 |
| IPI00219585 | P08237 | Tax_Id=9606 Gene_Symbol=PFKM Isoform 2 of 6-phosphofructokinase, muscle type | Membrane | 130 | 82579 | 2 | 3.6 |
| IPI00002406 | P50895 | Tax_Id=9606 Gene_Symbol=BCAM Basal cell adhesion molecule | Membrane | 130 | 68161 | 2 | 1.9 |
| IPI00328156 | P27338 | Tax_Id=9606 Gene_Symbol=MAOB Amine oxidase [flavin-containing] B | Membrane | 76 | 59238 | 2 | 4.8 |
| IPI00013421 | Q13491 | Tax_Id=9606 Gene_Symbol=GPM6B Isoform 1 of Neuronal membrane glycoprotein M6-b | Membrane | 66 | 29882 | 2 | 4.9 |
| IPI00297160 | P16070 | Tax_Id=9606 Gene_Symbol=CD44 Isoform 12 of CD44 antigen | Membrane | 59 | 39904 | 2 | 5.5 |
| IPI00009532 | P80404 | Tax_Id=9606 Gene_Symbol=ABAT cDNA FLJ56034, highly similar to 4-aminobutyrate aminotransferase, mitochondrial | Membrane | 99 | 58876 | 1 | 2.3 |
| IPI00000190 | P60033 | Tax_Id=9606 Gene_Symbol=CD81 CD81 antigen | Membrane | 97 | 26476 | 1 | 6.8 |
| IPI00026268 | P62873 | Tax_Id=9606 Gene_Symbol=GNB1 Guanine nucleotide-binding protein G(I)/G(S)/G(T) subunit beta-1 | Membrane | 92 | 38151 | 1 | 5.3 |
| IPI00028908 | Q14112 | Tax_Id=9606 Gene_Symbol=NID2 Nidogen-2 | Membrane | 81 | 154093 | 1 | 2.1 |
| IPI00021831 | P10644 | Tax_Id=9606 Gene_Symbol=PRKAR1A cAMP-dependent protein kinase type I-alpha regulatory subunit | Membrane | 79 | 43183 | 1 | 4.7 |
| IPI00007812 | P21281 | Tax_Id=9606 Gene_Symbol=ATP6V1B2 V-type proton ATPase subunit B, brain isoform | Membrane | 75 | 56807 | 1 | 4.3 |
| IPI00657936 | Q2UY09 | Tax_Id=9606 Gene_Symbol=COL28A1 Isoform 1 of Collagen alpha-1(XXVIII) chain | Membrane | 73 | 117440 | 1 | 2.3 |
| IPI00031522 | P40939 | Tax_Id=9606 Gene_Symbol=HADHA Trifunctional enzyme subunit alpha, mitochondrial | Membrane | 69 | 83688 | 1 | 1.8 |
| IPI00007682 | P38606 | Tax_Id=9606 Gene_Symbol=ATP6V1A V-type proton ATPase catalytic subunit A | Membrane | 67 | 68660 | 1 | 1.8 |
| IPI00015473 | P43003 | Tax_Id=9606 Gene_Symbol=SLC1A3 Excitatory amino acid transporter 1 | Membrane | 61 | 59705 | 1 | 3.7 |
| IPI00027252 | Q99623 | Tax_Id=9606 Gene_Symbol=PHB2 Prohibitin-2 | Membrane | 59 | 33276 | 1 | 3 |
| IPI00010796 | P07237 | Tax_Id=9606 Gene_Symbol=P4HB Protein disulfide-isomerase | Membrane | 55 | 57480 | 1 | 5.9 |
| IPI00019952 | P51674 | Tax_Id=9606 Gene_Symbol=GPM6A Neuronal membrane glycoprotein M6-a | Membrane | 51 | 31930 | 1 | 4 |
| IPI00024689 | P29972 | Tax_Id=9606 Gene_Symbol=AQP1 Aquaporin-1 | Membrane | 51 | 28736 | 1 | 2.6 |
| IPI00295469 | F5GXN1 | Tax_Id=9606 Gene_Symbol=CPNE6 cDNA FLJ55997, highly similar to Copine-6 | Membrane | 43 | 68598 | 1 | 1.1 |
| IPI00006471 | Q14680 | Tax_Id=9606 Gene_Symbol=MELK Maternal embryonic leucine zipper kinase | Membrane | 39 | 75507 | 1 | 1.1 |
| IPI00037448 | Q9UBQ7 | Tax_Id=9606 Gene_Symbol=GRHPR Glyoxylate reductase/hydroxypyruvate reductase | Membrane | 39 | 36045 | 1 | 7.6 |
| IPI00025363 | P14136 | Tax_Id=9606 Gene_Symbol=GFAP Isoform 1 of Glial fibrillary acidic protein | Cytoplasm | 18144 | 49907 | 696 | 80.1 |
| IPI00745872 | P02768 | Tax_Id=9606 Gene_Symbol=ALB Isoform 1 of Serum albumin | Cytoplasm | 5829 | 71317 | 219 | 76.7 |
| IPI00237671 | P07196 | Tax_Id=9606 Gene_Symbol=NEFL Neurofilament light polypeptide | Cytoplasm | 2760 | 61536 | 107 | 57.1 |
| IPI00418471 | P08670 | Tax_Id=9606 Gene_Symbol=VIM Vimentin | Cytoplasm | 2611 | 53676 | 104 | 68.5 |
| IPI00217507 | P07197 | Tax_Id=9606 Gene_Symbol=NEFM Neurofilament medium polypeptide | Cytoplasm | 1952 | 102468 | 81 | 27.2 |
| IPI00843765 | Q13813 | Tax_Id=9606 Gene_Symbol=SPTAN1 Isoform 3 of Spectrin alpha chain, brain | Cytoplasm | 2233 | 282906 | 64 | 26.2 |
| IPI00180675 | Q71U36 | Tax_Id=9606 Gene_Symbol=TUBA1A Tubulin alpha-1A chain | Cytoplasm | 1745 | 50788 | 54 | 51.9 |
| IPI00792677 | B4DDU2 | Tax_Id=9606 Gene_Symbol=TUBA1B cDNA FLJ60097, highly similar to Tubulin alpha-ubiquitous chain | Cytoplasm | 1700 | 46797 | 54 | 56.2 |
| IPI00031370 | Q9BVA1 | Tax_Id=9606 Gene_Symbol=TUBB2B Tubulin beta-2B chain | Cytoplasm | 1655 | 50377 | 52 | 62.9 |
| IPI00013475 | Q13885 | Tax_Id=9606 Gene_Symbol=TUBB2A Tubulin beta-2A chain | Cytoplasm | 1654 | 50274 | 52 | 62.9 |
| IPI00909140 | P07436 | Tax_Id=9606 Gene_Symbol=TUBB Tubulin beta chain | Cytoplasm | 1400 | 50095 | 49 | 53.2 |
| IPI00465248 | P06733 | Tax_Id=9606 Gene_Symbol=ENO1 Isoform alpha-enolase of Alpha-enolase | Cytoplasm | 1764 | 47481 | 46 | 65 |
| IPI00007752 | P68371 | Tax_Id=9606 Gene_Symbol=TUBB2C Tubulin beta-2C chain | Cytoplasm | 1339 | 50255 | 45 | 54.2 |
| IPI00923396 | P12036 | Tax_Id=9606 Gene_Symbol=NEFH Isoform 2 of Neurofilament heavy polypeptide | Cytoplasm | 1187 | 105804 | 43 | 19.8 |
| IPI00021439 | P60709 | Tax_Id=9606 Gene_Symbol=ACTB Actin, cytoplasmic 1 | Cytoplasm | 1146 | 42052 | 37 | 54.7 |
| IPI00013683 | Q13509 | Tax_Id=9606 Gene_Symbol=TUBB3 Tubulin beta-3 chain | Cytoplasm | 1024 | 50856 | 36 | 38.4 |
| IPI00023598 | P04350 | Tax_Id=9606 Gene_Symbol=TUBB4 Tubulin beta-4 chain | Cytoplasm | 1042 | 50010 | 34 | 49.8 |
| IPI00005614 | Q01082 | Tax_Id=9606 Gene_Symbol=SPTBN1 Isoform Long of Spectrin beta chain, brain 1 | Cytoplasm | 893 | 275237 | 34 | 12.5 |
| IPI00257508 | Q16555 | Tax_Id=9606 Gene_Symbol=DPYSL2 Dihydropyrimidinase-related protein 2 | Cytoplasm | 1266 | 62711 | 30 | 49.8 |
| IPI00022977 | P12277 | Tax_Id=9606 Gene_Symbol=CKB Creatine kinase B-type | Cytoplasm | 1185 | 42902 | 29 | 36.2 |
| IPI00219018 | P04406 | Tax_Id=9606 Gene_Symbol=GAPDH Glyceraldehyde-3-phosphate dehydrogenase | Cytoplasm | 994 | 36201 | 27 | 59.7 |
| IPI00382470 | P07900 | Tax_Id=9606 Gene_Symbol=HSP90AA1 Isoform 2 of Heat shock protein HSP 90-alpha | Cytoplasm | 595 | 98670 | 25 | 27.9 |
| IPI00021428 | P68133 | Tax_Id=9606 Gene_Symbol=ACTA1 Actin, alpha skeletal muscle | Cytoplasm | 549 | 42366 | 21 | 31.3 |
| IPI00654755 | P68871 | Tax_Id=9606 Gene_Symbol=HBB Hemoglobin subunit beta | Cytoplasm | 656 | 16102 | 20 | 61.9 |
| IPI00013164 | P41219 | Tax_Id=9606 Gene_Symbol=PRPH Isoform 1 of Peripherin | Cytoplasm | 584 | 53732 | 20 | 34.9 |
| IPI00014898 | Q15149 | Tax_Id=9606 Gene_Symbol=PLEC1 Isoform 1 of Plectin-1 | Cytoplasm | 347 | 533462 | 19 | 4.3 |
| IPI00029111 | Q6DEN2 | Tax_Id=9606 Gene_Symbol=DPYSL3 Collapsin response mediator protein 4 long variant | Cytoplasm | 704 | 74321 | 18 | 32.3 |
| IPI00954527 | Q13707 | Tax_Id=9606 Gene_Symbol=ACTA2 ACTA2 protein (Fragment) | Cytoplasm | 527 | 37125 | 18 | 30.3 |
| IPI00414676 | P08238 | Tax_Id=9606 Gene_Symbol=HSP90AB1 Heat shock protein HSP 90-beta | Cytoplasm | 324 | 83554 | 15 | 19.9 |
| IPI00004358 | P11216 | Tax_Id=9606 Gene_Symbol=PYGB Glycogen phosphorylase, brain form | Cytoplasm | 443 | 97319 | 13 | 17.2 |
| IPI00010154 | P31150 | Tax_Id=9606 Gene_Symbol=GDI1 Rab GDP dissociation inhibitor alpha | Cytoplasm | 529 | 51177 | 12 | 40 |
| IPI00643920 | P29401 | Tax_Id=9606 Gene_Symbol=TKT cDNA FLJ54957, highly similar to Transketolase | Cytoplasm | 273 | 69382 | 12 | 20 |
| IPI00218130 | P11217 | Tax_Id=9606 Gene_Symbol=PYGM Glycogen phosphorylase, muscle form | Cytoplasm | 267 | 97487 | 12 | 12.4 |
| IPI00911039 | P08107 | Tax_Id=9606 Gene_Symbol=HSPA1A;HSPA1B cDNA FLJ54408, highly similar to Heat shock 70 kDa protein 1 | Cytoplasm | 348 | 64170 | 11 | 16.2 |
| IPI00784154 | P10809 | Tax_Id=9606 Gene_Symbol=HSPD1 60 kDa heat shock protein, mitochondrial | Cytoplasm | 222 | 61187 | 11 | 20.8 |
| IPI00304925 | P08107 | Tax_Id=9606 Gene_Symbol=HSPA1A;HSPA1B Heat shock 70 kDa protein 1A/1B | Cytoplasm | 334 | 70294 | 10 | 17.9 |
| IPI00007702 | P54652 | Tax_Id=9606 Gene_Symbol=HSPA2 Heat shock-related 70 kDa protein 2 | Cytoplasm | 272 | 70263 | 9 | 10 |
| IPI00219217 | P07195 | Tax_Id=9606 Gene_Symbol=LDHB L-lactate dehydrogenase B chain | Cytoplasm | 400 | 36900 | 8 | 27.2 |
| IPI00291136 | P12109 | Tax_Id=9606 Gene_Symbol=COL6A1 Collagen alpha-1(VI) chain | Cytoplasm | 122 | 109602 | 8 | 12.1 |
| IPI00026216 | P55786 | Tax_Id=9606 Gene_Symbol=NPEPPS Puromycin-sensitive aminopeptidase | Cytoplasm | 291 | 103895 | 7 | 6.5 |
| IPI00177728 | Q96KP4 | Tax_Id=9606 Gene_Symbol=CNDP2 Isoform 1 of Cytosolic non-specific dipeptidase | Cytoplasm | 272 | 53187 | 7 | 19.6 |
| IPI00022774 | P55072 | Tax_Id=9606 Gene_Symbol=VCP Transitional endoplasmic reticulum ATPase | Cytoplasm | 274 | 89950 | 6 | 8.4 |
| IPI00002459 | E5RJR0 | Tax_Id=9606 Gene_Symbol=ANXA6 annexin VI isoform 2 | Cytoplasm | 163 | 75571 | 6 | 13.8 |
| IPI00011200 | O43175 | Tax_Id=9606 Gene_Symbol=PHGDH D-3-phosphoglycerate dehydrogenase | Cytoplasm | 193 | 57356 | 4 | 13.3 |
| IPI00410714 | P69905 | Tax_Id=9606 Gene_Symbol=HBA2;HBA1 Hemoglobin subunit alpha | Cytoplasm | 170 | 15305 | 4 | 10.6 |
| IPI00645078 | P22314 | Tax_Id=9606 Gene_Symbol=UBA1 Ubiquitin-like modifier-activating enzyme 1 | Cytoplasm | 170 | 118858 | 4 | 6.2 |
| IPI00033025 | E7EPK1 | Tax_Id=9606 Gene_Symbol=SEPT7 51 kDa protein | Cytoplasm | 155 | 50820 | 4 | 7.1 |
| IPI00479877 | P49189 | Tax_Id=9606 Gene_Symbol=ALDH9A1 aldehyde dehydrogenase 9A1 | Cytoplasm | 125 | 57168 | 4 | 6.4 |
| IPI00019376 | Q9NVA2 | Tax_Id=9606 Gene_Symbol=SEPT11 Isoform 2 of Septin-11 | Cytoplasm | 55 | 51076 | 4 | 8.4 |
| IPI00003362 | P11021 | Tax_Id=9606 Gene_Symbol=HSPA5 HSPA5 protein | Cytoplasm | 81 | 72492 | 3 | 5.2 |
| IPI00002460 | P20073 | Tax_Id=9606 Gene_Symbol=ANXA7 Isoform 1 of Annexin A7 | Cytoplasm | 48 | 52991 | 3 | 6.8 |
| IPI00550069 | P13489 | Tax_Id=9606 Gene_Symbol=RNH1 Ribonuclease inhibitor | Cytoplasm | 115 | 51766 | 2 | 7.4 |
| IPI00746030 | P51178 | Tax_Id=9606 Gene_Symbol=PLCD1 1-phosphatidylinositol-4,5-bisphosphate phosphodiesterase delta-1 | Cytoplasm | 92 | 86352 | 2 | 6.1 |
| IPI00008994 | Q9UN36 | Tax_Id=9606 Gene_Symbol=NDRG2 Isoform 1 of Protein NDRG2 | Cytoplasm | 86 | 41114 | 2 | 11.6 |
| IPI00294187 | Q9Y2J8 | Tax_Id=9606 Gene_Symbol=PADI2 Protein-arginine deiminase type-2 | Cytoplasm | 85 | 76257 | 2 | 5.3 |
| IPI00020984 | B4DGP8 | Tax_Id=9606 Gene_Symbol=CANX cDNA FLJ55574, highly similar to Calnexin | Cytoplasm | 79 | 71971 | 2 | 4.8 |
| IPI00218820 | P07951 | Tax_Id=9606 Gene_Symbol=TPM2 Isoform 3 of Tropomyosin beta chain | Cytoplasm | 76 | 28666 | 2 | 13.3 |
| IPI00169383 | P00558 | Tax_Id=9606 Gene_Symbol=PGK1 Phosphoglycerate kinase 1 | Cytoplasm | 74 | 44985 | 2 | 5.8 |
| IPI00156689 | Q99536 | Tax_Id=9606 Gene_Symbol=VAT1 Synaptic vesicle membrane protein VAT-1 homolog | Cytoplasm | 67 | 42122 | 2 | 12.5 |
| IPI00018465 | Q99832 | Tax_Id=9606 Gene_Symbol=CCT7 T-complex protein 1 subunit eta | Cytoplasm | 65 | 59842 | 2 | 7 |
| IPI00909108 | B4DS85 | Tax_Id=9606 Gene_Symbol=NEUROD6 cDNA FLJ56829, highly similar to Neurogenic differentiation factor 6 | Cytoplasm | 63 | 59985 | 2 | 6 |
| IPI00289862 | Q12765 | Tax_Id=9606 Gene_Symbol=SCRN1 Secernin-1 | Cytoplasm | 55 | 46980 | 2 | 7.2 |
| IPI00020599 | P27797 | Tax_Id=9606 Gene_Symbol=CALR Calreticulin | Cytoplasm | 55 | 48283 | 2 | 2.2 |
| IPI00220271 | P14550 | Tax_Id=9606 Gene_Symbol=AKR1A1 Alcohol dehydrogenase [NADP+] | Cytoplasm | 51 | 36892 | 2 | 12.9 |
| IPI00419903 | 6ND91 | Tax_Id=9606 Gene_Symbol=ASPDH Putative L-aspartate dehydrogenase | Cytoplasm | 49 | 30213 | 2 | 2.8 |
| IPI00027230 | P14625 | Tax_Id=9606 Gene_Symbol=HSP90B1 Endoplasmin | Cytoplasm | 44 | 92696 | 2 | 3.9 |
| IPI00012268 | Q13200 | Tax_Id=9606 Gene_Symbol=PSMD2 26S proteasome non-ATPase regulatory subunit 2 | Cytoplasm | 44 | 100877 | 2 | 2.4 |
| IPI00413641 | P15121 | Tax_Id=9606 Gene_Symbol=AKR1B1 Aldose reductase | Cytoplasm | 39 | 36230 | 2 | 14.6 |
| IPI00166680 | Q8N4C8 | Tax_Id=9606 Gene_Symbol=MINK1 Isoform 3 of Misshapen-like kinase 1 | Cytoplasm | 35 | 150401 | 2 | 0.6 |
| IPI00216951 | P14868 | Tax_Id=9606 Gene_Symbol=DARS Aspartyl-tRNA synthetase, cytoplasmic | Cytoplasm | 35 | 57499 | 2 | 1.6 |
| IPI00219525 | P52209 | Tax_Id=9606 Gene_Symbol=PGD 6-phosphogluconate dehydrogenase, decarboxylating | Cytoplasm | 99 | 53619 | 1 | 2.9 |
| IPI00479201 | O43865 | Tax_Id=9606 Gene_Symbol=AHCYL1 Isoform 2 of Putative adenosylhomocysteinase 2 | Cytoplasm | 86 | 54802 | 1 | 3.3 |
| IPI00025252 | P30101 | Tax_Id=9606 Gene_Symbol=PDIA3 Protein disulfide-isomerase A3 | Cytoplasm | 86 | 57146 | 1 | 2.2 |
| IPI00001639 | Q14974 | Tax_Id=9606 Gene_Symbol=KPNB1 Importin subunit beta-1 | Cytoplasm | 83 | 98420 | 1 | 1.7 |
| IPI00221234 | P49419 | Tax_Id=9606 Gene_Symbol=ALDH7A1 aldehyde dehydrogenase 7 family, member A1 | Cytoplasm | 83 | 59020 | 1 | 2.2 |
| IPI00291175 | P18206 | Tax_Id=9606 Gene_Symbol=VCL Isoform 1 of Vinculin | Cytoplasm | 79 | 117220 | 1 | 1.8 |
| IPI00056334 | Q969G5 | Tax_Id=9606 Gene_Symbol=PRKCDBP Protein kinase C delta-binding protein | Cytoplasm | 75 | 27609 | 1 | 4.2 |
| IPI00294578 | P21980 | Tax_Id=9606 Gene_Symbol=TGM2 Isoform 1 of Protein-glutamine gamma-glutamyltransferase 2 | Cytoplasm | 67 | 78420 | 1 | 2.3 |
| IPI00000874 | Q06830 | Tax_Id=9606 Gene_Symbol=PRDX1 Peroxiredoxin-1 | Cytoplasm | 61 | 22324 | 1 | 5.5 |
| IPI00220342 | O94760 | Tax_Id=9606 Gene_Symbol=DDAH1 N(G),N(G)-dimethylarginine dimethylaminohydrolase 1 | Cytoplasm | 56 | 31444 | 1 | 4.2 |
| IPI00419237 | P28838 | Tax_Id=9606 Gene_Symbol=LAP3 Isoform 1 of Cytosol aminopeptidase | Cytoplasm | 53 | 56530 | 1 | 2.3 |
| IPI00332887 |  | Tax_Id=9606 Gene_Symbol=SIRPA signal-regulatory protein alpha precursor | Cytoplasm | 49 | 55446 | 1 | 2.2 |
| IPI00167821 | Q8N9V2 | Tax_Id=9606 Gene_Symbol=TRIML1 Tripartite motif family-like protein 1 | Cytoplasm | 48 | 53824 | 1 | 1.9 |
| IPI00217872 | P36871 | Tax_Id=9606 Gene_Symbol=PGM1 Isoform 2 of Phosphoglucomutase-1 | Cytoplasm | 40 | 64092 | 1 | 1.4 |
| IPI00791397 |  | Tax_Id=9606 Gene_Symbol=CCNE2 33 kDa protein | Cytoplasm | 39 | 33641 | 1 | 2.4 |
| IPI00008868 | P46821 | Tax_Id=9606 Gene_Symbol=MAP1B Microtubule-associated protein 1B | Cytoplasm | 35 | 271651 | 1 | 0.7 |
|  |  |  |  |  |  |  |  |
| IPI00479186 | P14618 | Tax_Id=9606 Gene_Symbol=PKM2 Isoform M2 of Pyruvate kinase isozymes M1/M2 | Nucleus | 970 | 58470 | 33 | 35.2 |
| IPI00024853 | Q9BXM0 | Tax_Id=9606 Gene_Symbol=PRX Isoform 1 of Periaxin | Nucleus | 1061 | 155248 | 31 | 17.9 |
| IPI00003865 | P11142 | Tax_Id=9606 Gene_Symbol=HSPA8 Isoform 1 of Heat shock cognate 71 kDa protein | Nucleus | 406 | 71082 | 15 | 22.9 |
| IPI00760877 | A6H8Y1 | Tax_Id=9606 Gene_Symbol=BDP1 Isoform 1 of Transcription factor TFIIIB component B~~ homolog | Nucleus | 36 | 295427 | 4 | 0.9 |
| IPI00179330 | P62979 | Tax_Id=9606 Gene_Symbol=UBC;RPS27A;UBB ubiquitin and ribosomal protein S27a precursor | Nucleus | 82 | 18296 | 3 | 21.8 |
| IPI00783392 | Q8TDY2 | Tax_Id=9606 Gene_Symbol=RB1CC1 RB1-inducible coiled-coil protein 1 | Nucleus | 40 | 185085 | 3 | 0.9 |
| IPI00154528 | Q96SB8 | Tax_Id=9606 Gene_Symbol=SMC6 Isoform 1 of Structural maintenance of chromosomes protein 6 | Nucleus | 40 | 127216 | 3 | 1.3 |
| IPI00021626 | Q9BTD8 | Tax_Id=9606 Gene_Symbol=RBM42 Isoform 1 of RNA-binding protein 42 | Nucleus | 38 | 50496 | 3 | 1.7 |
| IPI00012303 | Q13228 | Tax_Id=9606 Gene_Symbol=SELENBP1 Selenium binding protein 1 | Nucleus | 138 | 57457 | 2 | 8.2 |
| IPI00396378 | P22626 | Tax_Id=9606 Gene_Symbol=HNRNPA2B1 Isoform B1 of Heterogeneous nuclear ribonucleoproteins A2/B1 | Nucleus | 64 | 37464 | 2 | 2.8 |
| IPI00018353 | Q9UJ98 | Tax_Id=9606 Gene_Symbol=STAG3 Isoform 1 of Cohesin subunit SA-3 | Nucleus | 36 | 140257 | 2 | 0.7 |
| IPI00220834 | P13010 | Tax_Id=9606 Gene_Symbol=XRCC5 ATP-dependent DNA helicase 2 subunit 2 | Nucleus | 47 | 83222 | 1 | 3.6 |
| IPI00479786 | Q92945 | Tax_Id=9606 Gene_Symbol=KHSRP KH-type splicing regulatory protein | Nucleus | 37 | 73355 | 1 | 2.1 |
| IPI00022463 | P02787 | Tax_Id=9606 Gene_Symbol=TF Serotransferrin | Secreted | 1164 | 79280 | 32 | 42.3 |
| IPI00026314 | P06396 | Tax_Id=9606 Gene_Symbol=GSN Isoform 1 of Gelsolin | Secreted | 800 | 86043 | 25 | 35.5 |
| IPI00020987 | P51888 | Tax_Id=9606 Gene_Symbol=PRELP Prolargin | Secreted | 307 | 44181 | 17 | 22.8 |
| IPI00022200 | P12111 | Tax_Id=9606 Gene_Symbol=COL6A3 Isoform 1 of Collagen alpha-3(VI) chain | Secreted | 396 | 345163 | 15 | 4.9 |
| IPI00010790 | P21810 | Tax_Id=9606 Gene_Symbol=BGN Biglycan | Secreted | 276 | 42027 | 11 | 16.6 |
| IPI00418169 | P07355 | Tax_Id=9606 Gene_Symbol=ANXA2 Isoform 2 of Annexin A2 | Secreted | 371 | 40671 | 9 | 23.5 |
| IPI00553177 | P01009 | Tax_Id=9606 Gene_Symbol=SERPINA1 Isoform 1 of Alpha-1-antitrypsin | Secreted | 313 | 46878 | 9 | 23.4 |
| IPI00297646 | Q14042 | Tax_Id=9606 Gene_Symbol=COL1A1 Collagen alpha-1(I) chain | Secreted | 291 | 139853 | 9 | 3.5 |
| IPI00012119 | P07585 | Tax_Id=9606 Gene_Symbol=DCN Isoform A of Decorin | Secreted | 131 | 40064 | 7 | 14.2 |
| IPI00020986 | P51884 | Tax_Id=9606 Gene_Symbol=LUM Lumican | Secreted | 137 | 38747 | 6 | 14.2 |
| IPI00550991 | P01011 | Tax_Id=9606 Gene_Symbol=SERPINA3 cDNA FLJ35730 fis, clone TESTI2003131, highly similar to ALPHA-1-ANTICHYMOTRYPSIN | Secreted | 324 | 50737 | 5 | 10.7 |
| IPI00025465 | P20774 | Tax_Id=9606 Gene_Symbol=OGN cDNA FLJ59205, highly similar to Mimecan | Secreted | 224 | 40870 | 5 | 19.7 |
| IPI00176193 | Q05707 | Tax_Id=9606 Gene_Symbol=COL14A1 Isoform 1 of Collagen alpha-1(XIV) chain | Secreted | 80 | 194478 | 5 | 3.3 |
| IPI00298281 | P11047 | Tax_Id=9606 Gene_Symbol=LAMC1 Laminin subunit gamma-1 | Secreted | 141 | 183191 | 4 | 3.8 |
| IPI00304840 | P12110 | Tax_Id=9606 Gene_Symbol=COL6A2 Isoform 2C2 of Collagen alpha-2(VI) chain | Secreted | 44 | 109709 | 4 | 6.3 |
| IPI00304962 | P08123 | Tax_Id=9606 Gene_Symbol=COL1A2 Collagen alpha-2(I) chain | Secreted | 153 | 129723 | 3 | 2.4 |
| IPI00296922 | P55268 | Tax_Id=9606 Gene_Symbol=LAMB2 Laminin subunit beta-2 | Secreted | 133 | 202982 | 3 | 2.2 |
| IPI00022429 | P02763 | Tax_Id=9606 Gene_Symbol=ORM1 Alpha-1-acid glycoprotein 1 | Secreted | 113 | 23725 | 3 | 14.4 |
| IPI00783987 | P01024 | Tax_Id=9606 Gene_Symbol=C3 Complement C3 (Fragment) | Secreted | 97 | 188569 | 3 | 2 |
| IPI00022488 | P02790 | Tax_Id=9606 Gene_Symbol=HPX Hemopexin | Secreted | 55 | 52385 | 3 | 10 |
| IPI00218725 | P24043 | Tax_Id=9606 Gene_Symbol=LAMA2 laminin alpha 2 subunit isoform b precursor | Secreted | 50 | 352589 | 3 | 1 |
| IPI00024621 | Q9NRN5 | Tax_Id=9606 Gene_Symbol=OLFML3 Isoform 1 of Olfactomedin-like protein 3 | Secreted | 76 | 46380 | 2 | 2.5 |
| IPI00478003 | P01023 | Tax_Id=9606 Gene_Symbol=A2M Alpha-2-macroglobulin | Secreted | 68 | 164614 | 2 | 3.1 |
| IPI00298497 | P02675 | Tax_Id=9606 Gene_Symbol=FGB Fibrinogen beta chain | Secreted | 58 | 56577 | 2 | 8.8 |
| IPI00013976 | P07942 | Tax_Id=9606 Gene_Symbol=LAMB1 Laminin subunit beta-1 | Secreted | 62 | 205178 | 1 | 1.2 |
| IPI00555812 | P02774 | Tax_Id=9606 Gene_Symbol=GC Isoform 1 of Vitamin D-binding protein | Secreted | 54 | 54526 | 1 | 4.6 |
| IPI00024284 | P98160 | Tax_Id=9606 Gene_Symbol=HSPG2 Basement membrane-specific heparan sulfate proteoglycan core protein | Secreted | 53 | 479221 | 1 | 0.4 |
| IPI00292836 | Q9P1Z9 | Tax_Id=9606 Gene_Symbol=KIAA1529 Isoform 1 of Unknown protein KIAA1529 | Unknown | 55 | 192404 | 28 | 1.8 |
| IPI00942772 |  | Tax_Id=9606 Gene_Symbol=TUBA4B Putative Unknown protein ENSP00000403894 | Unknown | 57 | 30377 | 4 | 7.5 |
| IPI00745035 |  | Tax_Id=9606 Gene_Symbol=- Putative Unknown protein ENSP00000339838 | Unknown | 47 | 15103 | 2 | 25.6 |

**Supplementary** **S5:** Proteins identified by LC-MSMS, sample MS2

| **Protein hit** | **Protein Accession Number** | **Uniprot Entry** | **Protein Description** | **Sub cellular location** | **Protein Score** | **Protein Mass** | **Protein matches** | **Protein coverage** |
| --- | --- | --- | --- | --- | --- | --- | --- | --- |
| 19 | IPI00219661 | P60201 | Tax_Id=9606 Gene_Symbol=PLP1 Isoform 1 of Myelin proteolipid protein | Membrane | 418 | 30855 | 36 | 26.7 |
| 33 | IPI00220993 | P09543 | Tax_Id=9606 Gene_Symbol=CNP Isoform CNPI of 2~,3~-cyclic-nucleotide 3~-phosphodiesterase | Cyto | 786 | 45469 | 20 | 40.4 |
| 80 | IPI00556079 | Q16653 | Tax_Id=9606 Gene_Symbol=MOG Isoform 1 of Myelin-oligodendrocyte glycoprotein | Membrane | 188 | 28560 | 4 | 14.6 |
| 36 | IPI00303476 | P06576 | Tax_Id=9606 Gene_Symbol=ATP5B ATP synthase subunit beta, mitochondrial | Membrane | 765 | 56525 | 17 | 41.4 |
| 38 | IPI00017855 | Q99798 | Tax_Id=9606 Gene_Symbol=ACO2 Aconitate hydratase, mitochondrial | Membrane | 625 | 86113 | 15 | 20.3 |
| 40 | IPI00022774 | P55072 | Tax_Id=9606 Gene_Symbol=VCP Transitional endoplasmic reticulum ATPase | Membrane | 611 | 89950 | 14 | 18 |
| 42 | IPI00219365 | P26038 | Tax_Id=9606 Gene_Symbol=MSN Moesin | Membrane | 374 | 67892 | 12 | 14.2 |
| 43 | IPI00304925 | P08107 | Tax_Id=9606 Gene_Symbol=HSPA1A;HSPA1B Heat shock 70 kDa protein 1A/1B | Membrane | 371 | 70294 | 11 | 13.3 |
| 44 | IPI00003021 | P50993 | Tax_Id=9606 Gene_Symbol=ATP1A2 Sodium/potassium-transporting ATPase subunit alpha-2 | Membrane | 451 | 113505 | 10 | 10 |
| 46 | IPI00216171 | P09104 | Tax_Id=9606 Gene_Symbol=ENO2 Gamma-enolase | Membrane | 409 | 47581 | 10 | 24 |
| 49 | IPI00024067 | Q00610 | Tax_Id=9606 Gene_Symbol=CLTC Isoform 1 of Clathrin heavy chain 1 | Membrane | 476 | 193260 | 9 | 8.2 |
| 51 | IPI00647102 | B3KW93 | Tax_Id=9606 Gene_Symbol=ATP1A2 cDNA FLJ42590 fis, clone BRACE3009708, highly similar to Sodium/potassium-transporting ATPase alpha-2chain | Membrane | 411 | 102434 | 9 | 11.5 |
| 56 | IPI00239405 | Q8WXH0 | Tax_Id=9606 Gene_Symbol=SYNE2 Isoform 1 of Nesprin-2 | Membrane | 64 | 801817 | 8 | 0.2 |
| 67 | IPI00220737 | P13591 | Tax_Id=9606 Gene_Symbol=NCAM1 Isoform 3 of Neural cell adhesion molecule 1 | Membrane | 361 | 84345 | 5 | 8 |
| 71 | IPI00007682 | P38606 | Tax_Id=9606 Gene_Symbol=ATP6V1A V-type proton ATPase catalytic subunit A | Membrane | 234 | 68660 | 5 | 5.7 |
| 76 | IPI00220558 | P21817 | Tax_Id=9606 Gene_Symbol=RYR1 Isoform 2 of Ryanodine receptor 1 | Membrane | 71 | 570033 | 5 | 0.5 |
| 79 | IPI00658109 | P12532 | Tax_Id=9606 Gene_Symbol=CKMT1B;CKMT1A Isoform 1 of Creatine kinase U-type, mitochondrial | Membrane | 212 | 47406 | 4 | 13.4 |
| 82 | IPI00016801 | P00367 | Tax_Id=9606 Gene_Symbol=GLUD1 Glutamate dehydrogenase 1, mitochondrial | Membrane | 150 | 61701 | 4 | 4.8 |
| 83 | IPI00297160 | P16070 | Tax_Id=9606 Gene_Symbol=CD44 Isoform 12 of CD44 antigen | Membrane | 108 | 39904 | 4 | 5.8 |
| 84 | IPI00748783 | Q13507 | Tax_Id=9606 Gene_Symbol=TRPC3 Short transient receptor potential channel 3 | Membrane | 61 | 98090 | 4 | 1.7 |
| 96 | IPI00291006 | P40926 | Tax_Id=9606 Gene_Symbol=MDH2 Malate dehydrogenase, mitochondrial | Membrane | 143 | 35937 | 2 | 8 |
| 97 | IPI00046057 | P61764 | Tax_Id=9606 Gene_Symbol=STXBP1 Isoform 2 of Syntaxin-binding protein 1 | Membrane | 142 | 69091 | 2 | 3.8 |
| 99 | IPI00219585 | P08237 | Tax_Id=9606 Gene_Symbol=PFKM Isoform 2 of 6-phosphofructokinase, muscle type | Membrane | 139 | 82579 | 2 | 3.9 |
| 104 | IPI00328156 | P27338 | Tax_Id=9606 Gene_Symbol=MAOB Amine oxidase [flavin-containing] B | Membrane | 96 | 59238 | 2 | 6 |
| 108 | IPI00018246 | P19367 | Tax_Id=9606 Gene_Symbol=HK1 Isoform 1 of Hexokinase-1 | Membrane | 72 | 103561 | 2 | 1.3 |
| 109 | IPI00015473 | P43003 | Tax_Id=9606 Gene_Symbol=SLC1A3 Excitatory amino acid transporter 1 | Membrane | 65 | 59705 | 2 | 3.7 |
| 113 | IPI00784154 | P10809 | Tax_Id=9606 Gene_Symbol=HSPD1 60 kDa heat shock protein, mitochondrial | Membrane | 57 | 61187 | 2 | 5.4 |
| 118 | IPI00328762 | Q86UQ4 | Tax_Id=9606 Gene_Symbol=ABCA13 Isoform 1 of ATP-binding cassette sub-family A member 13 | Membrane | 45 | 580604 | 2 | 0.4 |
| 132 | IPI00006663 | P05091 | Tax_Id=9606 Gene_Symbol=ALDH2 Aldehyde dehydrogenase, mitochondrial | Membrane | 74 | 56859 | 1 | 2.3 |
| 135 | IPI00007188 | P05141 | Tax_Id=9606 Gene_Symbol=SLC25A5 ADP/ATP translocase 2 | Membrane | 70 | 33102 | 1 | 4 |
| 136 | IPI00219301 | P29966 | Tax_Id=9606 Gene_Symbol=MARCKS Myristoylated alanine-rich C-kinase substrate | Membrane | 69 | 31707 | 1 | 5.7 |
| 137 | IPI00007812 | P21281 | Tax_Id=9606 Gene_Symbol=ATP6V1B2 V-type proton ATPase subunit B, brain isoform | Membrane | 68 | 56807 | 1 | 2.7 |
| 138 | IPI00747849 | P05026 | Tax_Id=9606 Gene_Symbol=ATP1B1 Isoform 1 of Sodium/potassium-transporting ATPase subunit beta-1 | Membrane | 68 | 35438 | 1 | 3.6 |
| 141 | IPI00299402 | P11498 | Tax_Id=9606 Gene_Symbol=PC Pyruvate carboxylase, mitochondrial | Membrane | 62 | 130293 | 1 | 1.2 |
| 143 | IPI00215997 | P21926 | Tax_Id=9606 Gene_Symbol=CD9 CD9 antigen | Membrane | 60 | 25969 | 1 | 11 |
| 144 | IPI00305383 | P22695 | Tax_Id=9606 Gene_Symbol=UQCRC2 Cytochrome b-c1 complex subunit 2, mitochondrial | Membrane | 58 | 48584 | 1 | 4.4 |
| 145 | IPI00022202 | Q00325 | Tax_Id=9606 Gene_Symbol=SLC25A3 Isoform A of Phosphate carrier protein, mitochondrial | Membrane | 58 | 40525 | 1 | 3.3 |
| 151 | IPI00019912 | P51659 | Tax_Id=9606 Gene_Symbol=HSD17B4 Peroxisomal multifunctional enzyme type 2 | Membrane | 44 | 80092 | 1 | 1.9 |
| 152 | IPI00167215 | Q14CZ8 | Tax_Id=9606 Gene_Symbol=HEPACAM Isoform 1 of Hepatocyte cell adhesion molecule | Membrane | 42 | 46226 | 1 | 4.6 |
| 154 | IPI00024143 | Q9NRG9 | Tax_Id=9606 Gene_Symbol=AAAS Aladin | Membrane | 42 | 60392 | 1 | 1.3 |
| 157 | IPI00180292 | Q9UQB8 | Tax_Id=9606 Gene_Symbol=BAIAP2 Isoform 5 of Brain-specific angiogenesis inhibitor 1-associated protein 2 | Membrane | 37 | 57637 | 1 | 1.9 |
| 158 | IPI00376956 | Q9P0W8 | Tax_Id=9606 Gene_Symbol=SPATA7 Isoform 2 of Spermatogenesis-associated protein 7 | Membrane | 35 | 64576 | 1 | 1.2 |
| 1 | IPI00025363 | P14136 | Tax_Id=9606 Gene_Symbol=GFAP Isoform 1 of Glial fibrillary acidic protein | Cytoplasm | 3019 | 49907 | 823 | 82.9 |
| 2 | IPI00237671 | P07196 | Tax_Id=9606 Gene_Symbol=NEFL Neurofilament light polypeptide | Cytoplasm | 1876 | 61536 | 104 | 51.4 |
| 3 | IPI00853115 | A5YM63 | Tax_Id=9606 Gene_Symbol=NEFM NEFM protein | Cytoplasm | 1471 | 98383 | 89 | 30.7 |
| 4 | IPI00745872 | P02768 | Tax_Id=9606 Gene_Symbol=ALB Isoform 1 of Serum albumin | Cytoplasm | 2056 | 71317 | 75 | 67 |
| 5 | IPI00418471 | P08670 | Tax_Id=9606 Gene_Symbol=VIM Vimentin | Cytoplasm | 1430 | 53676 | 65 | 56.4 |
| 6 | IPI00792677 | B4DDU2 | Tax_Id=9606 Gene_Symbol=TUBA1B cDNA FLJ60097, highly similar to Tubulin alpha-ubiquitous chain | Cytoplasm | 1207 | 46797 | 55 | 60.8 |
| 7 | IPI00180675 | Q71U36 | Tax_Id=9606 Gene_Symbol=TUBA1A Tubulin alpha-1A chain | Cytoplasm | 1204 | 50788 | 55 | 56.1 |
| 8 | IPI00843765 | Q13813 | Tax_Id=9606 Gene_Symbol=SPTAN1 Isoform 3 of Spectrin alpha chain, brain | Cytoplasm | 2217 | 282906 | 50 | 23.8 |
| 9 | IPI00013475 | Q13885 | Tax_Id=9606 Gene_Symbol=TUBB2A Tubulin beta-2A chain | Cytoplasm | 1380 | 50274 | 48 | 63.1 |
| 10 | IPI00007750 | P68366 | Tax_Id=9606 Gene_Symbol=TUBA4A Tubulin alpha-4A chain | Cytoplasm | 1071 | 50634 | 48 | 50 |
| 11 | IPI00218343 | Q9BQE3 | Tax_Id=9606 Gene_Symbol=TUBA1C Tubulin alpha-1C chain | Cytoplasm | 1068 | 50548 | 48 | 52.6 |
| 12 | IPI00007752 | P68371 | Tax_Id=9606 Gene_Symbol=TUBB2C Tubulin beta-2C chain | Cytoplasm | 1311 | 50255 | 46 | 57.1 |
| 13 | IPI00923396 | P12036 | Tax_Id=9606 Gene_Symbol=NEFH Isoform 2 of Neurofilament heavy polypeptide | Cytoplasm | 711 | 105804 | 45 | 13.1 |
| 14 | IPI00219018 | P04406 | Tax_Id=9606 Gene_Symbol=GAPDH Glyceraldehyde-3-phosphate dehydrogenase | Cytoplasm | 1167 | 36201 | 43 | 66.3 |
| 15 | IPI00909140 | P68371 | Tax_Id=9606 Gene_Symbol=TUBB Tubulin beta chain | Cytoplasm | 1192 | 50095 | 42 | 56.1 |
| 16 | IPI00023598 | P30883 | Tax_Id=9606 Gene_Symbol=TUBB4 Tubulin beta-4 chain | Cytoplasm | 1049 | 50010 | 39 | 47.7 |
| 17 | IPI00021439 | P60709 | Tax_Id=9606 Gene_Symbol=ACTB Actin, cytoplasmic 1 | Cytoplasm | 1155 | 42052 | 38 | 64.3 |
| 18 | IPI00257508 | Q16555 | Tax_Id=9606 Gene_Symbol=DPYSL2 Dihydropyrimidinase-related protein 2 | Cytoplasm | 1175 | 62711 | 36 | 51.9 |
| 20 | IPI00013683 | Q13509 | Tax_Id=9606 Gene_Symbol=TUBB3 Tubulin beta-3 chain | Cytoplasm | 1108 | 50856 | 35 | 43.3 |
| 21 | IPI00186711 | Q15149 | Tax_Id=9606 Gene_Symbol=PLEC1 Isoform 2 of Plectin-1 | Cytoplasm | 998 | 520095 | 32 | 7.3 |
| 23 | IPI00004358 | P11216 | Tax_Id=9606 Gene_Symbol=PYGB Glycogen phosphorylase, brain form | Cytoplasm | 987 | 97319 | 28 | 27.3 |
| 24 | IPI00465248 | P06733 | Tax_Id=9606 Gene_Symbol=ENO1 Isoform alpha-enolase of Alpha-enolase | Cytoplasm | 1157 | 47481 | 27 | 49.3 |
| 25 | IPI00382470 | P07900 | Tax_Id=9606 Gene_Symbol=HSP90AA1 Isoform 2 of Heat shock protein HSP 90-alpha | Cytoplasm | 1048 | 98670 | 27 | 25.5 |
| 26 | IPI00005614 | Q01082 | Tax_Id=9606 Gene_Symbol=SPTBN1 Isoform Long of Spectrin beta chain, brain 1 | Cytoplasm | 911 | 275237 | 26 | 8.8 |
| 27 | IPI00414676 | P08238 | Tax_Id=9606 Gene_Symbol=HSP90AB1 Heat shock protein HSP 90-beta | Cytoplasm | 822 | 83554 | 24 | 26.8 |
| 28 | IPI00022977 | P12277 | Tax_Id=9606 Gene_Symbol=CKB Creatine kinase B-type | Cytoplasm | 746 | 42902 | 24 | 43.6 |
| 29 | IPI00001453 | Q16352 | Tax_Id=9606 Gene_Symbol=INA Alpha-internexin | Cytoplasm | 342 | 55528 | 23 | 13 |
| 30 | IPI00219217 | P07195 | Tax_Id=9606 Gene_Symbol=LDHB L-lactate dehydrogenase B chain | Cytoplasm | 899 | 36900 | 22 | 43.1 |
| 31 | IPI00026314 | P06396 | Tax_Id=9606 Gene_Symbol=GSN Isoform 1 of Gelsolin | Cytoplasm | 852 | 86043 | 22 | 25.1 |
| 32 | IPI00021428 | P68133 | Tax_Id=9606 Gene_Symbol=ACTA1 Actin, alpha skeletal muscle | Cytoplasm | 565 | 42366 | 22 | 28.6 |
| 37 | IPI00003269 | Q562R1 | Tax_Id=9606 Gene_Symbol=ACTBL2 Beta-actin-like protein 2 | Cytoplasm | 303 | 42318 | 16 | 17.6 |
| 41 | IPI00007702 | P54652 | Tax_Id=9606 Gene_Symbol=HSPA2 Heat shock-related 70 kDa protein 2 | Cytoplasm | 390 | 70263 | 12 | 11.3 |
| 45 | IPI00643920 | P29401 | Tax_Id=9606 Gene_Symbol=TKT cDNA FLJ54957, highly similar to Transketolase | Cytoplasm | 450 | 69382 | 10 | 17.9 |
| 47 | IPI00221226 | P08133 | Tax_Id=9606 Gene_Symbol=ANXA6 Annexin A6 | Cytoplasm | 546 | 76168 | 9 | 14.3 |
| 48 | IPI00302840 | P13637 | Tax_Id=9606 Gene_Symbol=ATP1A3 Sodium/potassium-transporting ATPase subunit alpha-3 | Cytoplasm | 517 | 113102 | 9 | 10.3 |
| 50 | IPI00178352 | Q14315 | Tax_Id=9606 Gene_Symbol=FLNC Isoform 1 of Filamin-C | Cytoplasm | 436 | 293407 | 9 | 3.6 |
| 52 | IPI00916111 | P40925 | Tax_Id=9606 Gene_Symbol=MDH1 Malate dehydrogenase | Cytoplasm | 366 | 38916 | 9 | 33.5 |
| 53 | IPI00029111 | Q6DEN2 | Tax_Id=9606 Gene_Symbol=DPYSL3 Collapsin response mediator protein 4 long variant | Cytoplasm | 381 | 74321 | 8 | 13.5 |
| 54 | IPI00418262 | P09972 | Tax_Id=9606 Gene_Symbol=ALDOC Fructose-bisphosphate aldolase | Cytoplasm | 378 | 49062 | 8 | 16.4 |
| 55 | IPI00294187 | Q9Y2J8 | Tax_Id=9606 Gene_Symbol=PADI2 Protein-arginine deiminase type-2 | Cytoplasm | 360 | 76257 | 8 | 13.4 |
| 58 | IPI00013508 | P12814 | Tax_Id=9606 Gene_Symbol=ACTN1 Alpha-actinin-1 | Cytoplasm | 438 | 103563 | 7 | 9.4 |
| 59 | IPI00010154 | P31150 | Tax_Id=9606 Gene_Symbol=GDI1 Rab GDP dissociation inhibitor alpha | Cytoplasm | 364 | 51177 | 7 | 17.7 |
| 60 | IPI00013164 | P41219 | Tax_Id=9606 Gene_Symbol=PRPH Isoform 1 of Peripherin | Cytoplasm | 209 | 53732 | 7 | 9.8 |
| 61 | IPI00644892 | Q5TZA2 | Tax_Id=9606 Gene_Symbol=CROCC Isoform 2 of Rootletin | Cytoplasm | 69 | 148339 | 7 | 2.7 |
| 62 | IPI00217966 | P00338 | Tax_Id=9606 Gene_Symbol=LDHA Isoform 1 of L-lactate dehydrogenase A chain | Cytoplasm | 395 | 36950 | 6 | 17.5 |
| 63 | IPI00169383 | P00558 | Tax_Id=9606 Gene_Symbol=PGK1 Phosphoglycerate kinase 1 | Cytoplasm | 306 | 44985 | 6 | 12.9 |
| 65 | IPI00022465 | O14578 | Tax_Id=9606 Gene_Symbol=CIT Isoform 1 of Citron Rho-interacting kinase | Cytoplasm | 65 | 233339 | 6 | 0.7 |
| 69 | IPI00008994 | Q9UN36 | Tax_Id=9606 Gene_Symbol=NDRG2 Isoform 1 of Protein NDRG2 | Cytoplasm | 269 | 41114 | 5 | 19.9 |
| 73 | IPI00031522 | P40939 | Tax_Id=9606 Gene_Symbol=HADHA Trifunctional enzyme subunit alpha, mitochondrial | Cytoplasm | 207 | 83688 | 5 | 6.9 |
| 74 | IPI00027230 | P14625 | Tax_Id=9606 Gene_Symbol=HSP90B1 Endoplasmin | Cytoplasm | 196 | 92696 | 5 | 4.4 |
| 75 | IPI00465439 | P04075 | Tax_Id=9606 Gene_Symbol=ALDOA Fructose-bisphosphate aldolase A | Cytoplasm | 146 | 39851 | 5 | 16.8 |
| 81 | IPI00031461 | P50395 | Tax_Id=9606 Gene_Symbol=GDI2 cDNA FLJ60299, highly similar to Rab GDP dissociation inhibitor beta | Cytoplasm | 167 | 51577 | 4 | 8.5 |
| 85 | IPI00008756 | Q03001 | Tax_Id=9606 Gene_Symbol=DST dystonin isoform 3 | Cytoplasm | 48 | 636467 | 4 | 0.3 |
| 87 | IPI00645078 | P22314 | Tax_Id=9606 Gene_Symbol=UBA1 Ubiquitin-like modifier-activating enzyme 1 | Cytoplasm | 199 | 118858 | 3 | 5.3 |
| 88 | IPI00016461 | P51178 | Tax_Id=9606 Gene_Symbol=PLCD1 phospholipase C, delta 1 isoform 1 | Cytoplasm | 163 | 88878 | 3 | 5.9 |
| 89 | IPI00220342 | O94760 | Tax_Id=9606 Gene_Symbol=DDAH1 N(G),N(G)-dimethylarginine dimethylaminohydrolase 1 | Cytoplasm | 138 | 31444 | 3 | 8.4 |
| 91 | IPI00376483 | Q7Z7L7 | Tax_Id=9606 Gene_Symbol=ZER1 Protein zer-1 homolog | Cytoplasm | 50 | 89481 | 3 | 2 |
| 93 | IPI00657839 | Q9NXG0 | Tax_Id=9606 Gene_Symbol=CNTLN Isoform 2 of Centlein | Cytoplasm | 36 | 162673 | 3 | 0.6 |
| 94 | IPI00011200 | O43175 | Tax_Id=9606 Gene_Symbol=PHGDH D-3-phosphoglycerate dehydrogenase | Cytoplasm | 198 | 57356 | 2 | 6.9 |
| 95 | IPI00020984 | B4DGP8 | Tax_Id=9606 Gene_Symbol=CANX cDNA FLJ55574, highly similar to Calnexin | Cytoplasm | 146 | 71971 | 2 | 5.6 |
| 98 | IPI00007087 | Q9UK22 | Tax_Id=9606 Gene_Symbol=FBXO2 F-box only protein 2 | Cytoplasm | 141 | 33706 | 2 | 12.5 |
| 100 | IPI00219525 | P52209 | Tax_Id=9606 Gene_Symbol=PGD 6-phosphogluconate dehydrogenase, decarboxylating | Cytoplasm | 136 | 53619 | 2 | 7 |
| 102 | IPI00873656 | B4DNE4 | Tax_Id=9606 Gene_Symbol=SEPT7 cDNA FLJ52574, highly similar to Septin-7 | Cytoplasm | 122 | 45099 | 2 | 8.6 |
| 102 | IPI00873656 | B4DNE4 | Tax_Id=9606 Gene_Symbol=SEPT7 cDNA FLJ52574, highly similar to Septin-7 | Cytoplasm | 122 | 45099 | 2 | 8.6 |
| 116 | IPI00457284 | Q9UKJ3 | Tax_Id=9606 Gene_Symbol=GPATCH8 Isoform 1 of G patch domain-containing protein 8 | Cytoplasm | 49 | 165010 | 2 | 1.1 |
| 117 | IPI00398700 | P09471 | Tax_Id=9606 Gene_Symbol=GNAO1 Isoform Alpha-2 of Guanine nucleotide-binding protein G(o) subunit alpha | Cytoplasm | 46 | 40574 | 2 | 8.5 |
| 119 | IPI00434629 | Q9C0D6 | Tax_Id=9606 Gene_Symbol=FHDC1 FH2 domain-containing protein 1 | Cytoplasm | 44 | 125654 | 2 | 1.3 |
| 121 | IPI00030268 | Q5H9T9 | Tax_Id=9606 Gene_Symbol=FSCB Fibrous sheath CABYR-binding protein | Cytoplasm | 42 | 89134 | 2 | 2.3 |
| 122 | IPI00910300 | B4DUT0 | Tax_Id=9606 Gene_Symbol=BFAR cDNA FLJ58979, highly similar to Bifunctional apoptosis regulator | Cytoplasm | 41 | 38798 | 2 | 2.8 |
| 125 | IPI00425404 | Q7Z4S6 | Tax_Id=9606 Gene_Symbol=KIF21A Isoform 1 of Kinesin-like protein KIF21A | Cytoplasm | 37 | 188374 | 2 | 1.1 |
| 126 | IPI00005621 | Q9UH66 | Tax_Id=9606 Gene_Symbol=SYDE1 7h3 protein (Fragment) | Cytoplasm | 36 | 49963 | 2 | 1.8 |
| 127 | IPI00479877 | P49189 | Tax_Id=9606 Gene_Symbol=ALDH9A1 aldehyde dehydrogenase 9A1 | Cytoplasm | 33 | 57168 | 2 | 2.1 |
| 129 | IPI00165579 | Q96KP4 | Tax_Id=9606 Gene_Symbol=CNDP2 Isoform 2 of Cytosolic non-specific dipeptidase | Cytoplasm | 115 | 44090 | 1 | 5.4 |
| 131 | IPI00419237 | P28838 | Tax_Id=9606 Gene_Symbol=LAP3 Isoform 1 of Cytosol aminopeptidase | Cytoplasm | 81 | 56530 | 1 | 2.3 |
| 134 | IPI00029485 | Q14203 | Tax_Id=9606 Gene_Symbol=DCTN1 Isoform p150 of Dynactin subunit 1 | Cytoplasm | 73 | 142348 | 1 | 1.3 |
| 140 | IPI00424869 | Q6U7G8 | Tax_Id=9606 Gene_Symbol=RAP1GDS1 RAP1, GTP-GDP dissociation stimulator 1 isoform 6 | Cytoplasm | 62 | 57070 | 1 | 3.7 |
| 146 | IPI00037448 | Q9UBQ7 | Tax_Id=9606 Gene_Symbol=GRHPR Glyoxylate reductase/hydroxypyruvate reductase | Cytoplasm | 56 | 36045 | 1 | 7.6 |
| 148 | IPI00219526 | P36871 | Tax_Id=9606 Gene_Symbol=PGM1 Isoform 1 of Phosphoglucomutase-1 | Cytoplasm | 49 | 61696 | 1 | 2.7 |
| 153 | IPI00006451 | P46459 | Tax_Id=9606 Gene_Symbol=NSF Vesicle-fusing ATPase | Cytoplasm | 42 | 83055 | 1 | 1.2 |
| 155 | IPI00419903 | A6ND91 | Tax_Id=9606 Gene_Symbol=ASPDH Putative L-aspartate dehydrogenase | Cytoplasm | 40 | 30213 | 1 | 2.8 |
| 156 | IPI00220308 | P35240 | Tax_Id=9606 Gene_Symbol=NF2 Isoform 2 of Merlin | Cytoplasm | 37 | 72696 | 1 | 1.3 |
| 22 | IPI00479186 | P14618 | Tax_Id=9606 Gene_Symbol=PKM2 Isoform M2 of Pyruvate kinase isozymes M1/M2 | Nucleus | 1186 | 58470 | 28 | 45.6 |
| 34 | IPI00003865 | P11142 | Tax_Id=9606 Gene_Symbol=HSPA8 Isoform 1 of Heat shock cognate 71 kDa protein | Nucleus | 801 | 71082 | 19 | 25.9 |
| 35 | IPI00218130 | P11217 | Tax_Id=9606 Gene_Symbol=PYGM Glycogen phosphorylase, muscle form | Nucleus | 742 | 97487 | 19 | 23.6 |
| 64 | IPI00025447 | P68104 | Tax_Id=9606 Gene_Symbol=EEF1A1 Elongation factor 1-alpha | Nucleus | 154 | 48181 | 6 | 10.4 |
| 66 | IPI00783950 | Q8WZ42 | Tax_Id=9606 Gene_Symbol=TTN Isoform 6 of Titin | Nucleus | 54 | 634993 | 6 | 0.2 |
| 77 | IPI00783392 | Q8TDY2 | Tax_Id=9606 Gene_Symbol=RB1CC1 RB1-inducible coiled-coil protein 1 | Nucleus | 65 | 185085 | 5 | 0.9 |
| 78 | IPI00005264 | Q99959 | Tax_Id=9606 Gene_Symbol=PKP2 Isoform 2 of Plakophilin-2 | Nucleus | 49 | 97852 | 5 | 2.3 |
| 86 | IPI00400922 | Q14690 | Tax_Id=9606 Gene_Symbol=PDCD11 Protein RRP5 homolog | Nucleus | 46 | 209939 | 4 | 0.9 |
| 101 | IPI00001639 | Q14974 | Tax_Id=9606 Gene_Symbol=KPNB1 Importin subunit beta-1 | Nucleus | 123 | 98420 | 2 | 3.1 |
| 106 | IPI00026216 | P55786 | Tax_Id=9606 Gene_Symbol=NPEPPS Puromycin-sensitive aminopeptidase | Nucleus | 81 | 103895 | 2 | 1.4 |
| 107 | IPI00220556 | Q96AQ6 | Tax_Id=9606 Gene_Symbol=PBXIP1 Isoform 3 of Pre-B-cell leukemia transcription factor-interacting protein 1 | Nucleus | 76 | 73218 | 2 | 1.7 |
| 110 | IPI00155647 | O15018 | Tax_Id=9606 Gene_Symbol=PDZD2 Isoform 2 of PDZ domain-containing protein 2 | Nucleus | 59 | 282428 | 2 | 0.7 |
| 111 | IPI00641445 | Q5VTG6 | Tax_Id=9606 Gene_Symbol=NBPF9;NBPF8;NBPF10;NBPF14;KIAA1245;RP11-94I2.2;NBPF12;NBPF15;NBPF1 Novel protein | Nucleus | 58 | 78352 | 2 | 3.4 |
| 115 | IPI00044751 | Q96Q89 | Tax_Id=9606 Gene_Symbol=KIF20B Putative Unknown protein KIF20B | Nucleus | 52 | 215199 | 2 | 1 |
| 120 | IPI00301844 | Q9H4I2 | Tax_Id=9606 Gene_Symbol=ZHX3 Zinc fingers and homeoboxes protein 3 | Nucleus | 42 | 105447 | 2 | 1.5 |
| 123 | IPI00470896 | Q68DK2 | Tax_Id=9606 Gene_Symbol=ZFYVE26 Isoform 2 of Zinc finger FYVE domain-containing protein 26 | Nucleus | 40 | 287055 | 2 | 0.8 |
| 124 | IPI00107801 | P48378 | Tax_Id=9606 Gene_Symbol=RFX2 Isoform 2 of DNA-binding protein RFX2 | Nucleus | 37 | 77619 | 2 | 1.3 |
| 128 | IPI00383105 | Q3L8U1 | Tax_Id=9606 Gene_Symbol=CHD9 Isoform 1 of Chromodomain-helicase-DNA-binding protein 9 | Nucleus | 33 | 327986 | 2 | 0.4 |
| 147 | IPI00396378 | P22626 | Tax_Id=9606 Gene_Symbol=HNRNPA2B1 Isoform B1 of Heterogeneous nuclear ribonucleoproteins A2/B1 | Nucleus | 53 | 37464 | 1 | 2.8 |
| 150 | IPI00179330 | P62979 | Tax_Id=9606 Gene_Symbol=UBC;RPS27A;UBB ubiquitin and ribosomal protein S27a precursor | Nucleus | 47 | 18296 | 1 | 10.3 |
| 159 | IPI00218918 | P04083 | Tax_Id=9606 Gene_Symbol=ANXA1 Annexin A1 | Nucleus | 35 | 38918 | 1 | 3.8 |
| 39 | IPI00455315 | P07355 | Tax_Id=9606 Gene_Symbol=ANXA2 Isoform 1 of Annexin A2 | Secreted | 616 | 38808 | 15 | 42.8 |
| 70 | IPI00022463 | P02787 | Tax_Id=9606 Gene_Symbol=TF Serotransferrin | Secreted | 262 | 79280 | 5 | 9.2 |
| 90 | IPI00553177 | P01009 | Tax_Id=9606 Gene_Symbol=SERPINA1 Isoform 1 of Alpha-1-antitrypsin | Secreted | 92 | 46878 | 3 | 5.7 |
| 92 | IPI00015614 | P35030 | Tax_Id=9606 Gene_Symbol=PRSS3 Isoform A of Trypsin-3 | Secreted | 48 | 33306 | 3 | 4.9 |
| 105 | IPI00027497 | P06744 | Tax_Id=9606 Gene_Symbol=GPI Glucose-6-phosphate isomerase | Secreted | 90 | 63335 | 2 | 6.1 |
| 112 | IPI00291136 | P12109 | Tax_Id=9606 Gene_Symbol=COL6A1 Collagen alpha-1(VI) chain | Secreted | 58 | 109602 | 2 | 3.8 |
| 130 | IPI00550991 | P01011 | Tax_Id=9606 Gene_Symbol=SERPINA3 cDNA FLJ35730 fis, clone TESTI2003131, highly similar to ALPHA-1-ANTICHYMOTRYPSIN | Secreted | 86 | 50737 | 1 | 4.5 |
| 133 | IPI00025465 | P20774 | Tax_Id=9606 Gene_Symbol=OGN cDNA FLJ59205, highly similar to Mimecan | Secreted | 74 | 40870 | 1 | 4.5 |
| 139 | IPI00020987 | P51888 | Tax_Id=9606 Gene_Symbol=PRELP Prolargin | Secreted | 63 | 44181 | 1 | 3.1 |
| 57 | IPI00738806 | C9J6C0 | Tax_Id=9606 Gene_Symbol=MYO7B Putative Unknown protein MYO7B | Unknown | 58 | 243711 | 8 | 0.9 |
| 103 | IPI00384938 | Q7Z351 | Tax_Id=9606 Gene_Symbol=IGHV4-31;LOC100290320;LOC100294459;IGHG1 Putative Unknown protein DKFZp686N02209 | Unknown | 117 | 53503 | 2 | 7.5 |
| 160 | IPI00063805 | Q96IT6 | Tax_Id=9606 Gene_Symbol=C14orf128 Unknown protein C14orf128 | Unknown | 35 | 6102 | 1 | 14.3 |
| 161 | IPI00937890 |  | Tax_Id=9606 Gene_Symbol=LOC100287621;LOC100291050;LOC100291706 hypothetical protein XP_002342569 | Unknown | 33 | 13316 | 1 | 5.9 |
| 162 | IPI00022937 |  | Tax_Id=9606 Gene_Symbol=F5 252 kDa protein | Unknown | 33 | 253190 | 1 | 0.3 |

**Supplementary** **Table 6:** Proteins identified by LC-MSMS, sample MS3

| **Protein Accession Number** | **Uniprot Entry** | **Protein Description** | **Sub cellular location** | **Protein Score** | **Protein Mass** | **Protein matches** | **Protein coverage** |
| --- | --- | --- | --- | --- | --- | --- | --- |
| IPI00219661 | P60201 | Tax_Id=9606 Gene_Symbol=PLP1 Isoform 1 of Myelin proteolipid protein | Myelin | 396 | 30855 | 24 | 25.6 |
| IPI00220993 | P09543 | Tax_Id=9606 Gene_Symbol=CNP Isoform CNPI of 2~,3~-cyclic-nucleotide 3~-phosphodiesterase | Myelin | 215 | 45469 | 6 | 18 |
| IPI00219664 | Q16653 | Tax_Id=9606 Gene_Symbol=MOG Isoform 2 of Myelin-oligodendrocyte glycoprotein | Myelin | 77 | 23883 | 1 | 5.8 |
| IPI00178352 | Q14315 | Tax_Id=9606 Gene_Symbol=FLNC Isoform 1 of Filamin-C | Membrane | 2971 | 293407 | 69 | 25 |
| IPI00024067 | Q00610 | Tax_Id=9606 Gene_Symbol=CLTC Isoform 1 of Clathrin heavy chain 1 | Membrane | 1169 | 193260 | 20 | 15.1 |
| IPI00219217 | P07195 | Tax_Id=9606 Gene_Symbol=LDHB L-lactate dehydrogenase B chain | Membrane | 813 | 36900 | 16 | 44 |
| IPI00418169 | P07355 | Tax_Id=9606 Gene_Symbol=ANXA2 Isoform 2 of Annexin A2 | Membrane | 715 | 40671 | 15 | 41.5 |
| IPI00016801 | P00367 | Tax_Id=9606 Gene_Symbol=GLUD1 Glutamate dehydrogenase 1, mitochondrial | Membrane | 317 | 61701 | 11 | 13.4 |
| IPI00017855 | Q99798 | Tax_Id=9606 Gene_Symbol=ACO2 Aconitate hydratase, mitochondrial | Membrane | 281 | 86113 | 8 | 8.1 |
| IPI00029751 | Q12860 | Tax_Id=9606 Gene_Symbol=CNTN1 Isoform 1 of Contactin-1 | Membrane | 366 | 114104 | 7 | 8.4 |
| IPI00219365 | P26038 | Tax_Id=9606 Gene_Symbol=MSN Moesin | Membrane | 212 | 67892 | 7 | 11.1 |
| IPI00022799 | P55087 | Tax_Id=9606 Gene_Symbol=AQP4 Isoform 2 of Aquaporin-4 | Membrane | 166 | 35263 | 7 | 12.1 |
| IPI00440493 | P25705 | Tax_Id=9606 Gene_Symbol=ATP5A1 ATP synthase subunit alpha, mitochondrial | Membrane | 423 | 59828 | 6 | 11.2 |
| IPI00025753 | Q02413 | Tax_Id=9606 Gene_Symbol=DSG1 Desmoglein-1 | Membrane | 295 | 114670 | 6 | 7.3 |
| IPI00215997 | P21926 | Tax_Id=9606 Gene_Symbol=CD9 CD9 antigen | Membrane | 162 | 25969 | 6 | 21.5 |
| IPI00293251 | Q03001 | Tax_Id=9606 Gene_Symbol=DST Isoform 6 of Bullous pemphigoid antigen 1, isoforms 6/9/10 | Membrane | 74 | 593763 | 6 | 0.5 |
| IPI00006663 | P05091 | Tax_Id=9606 Gene_Symbol=ALDH2 Aldehyde dehydrogenase, mitochondrial | Membrane | 238 | 56859 | 5 | 11.8 |
| IPI00218487 | P17302 | Tax_Id=9606 Gene_Symbol=GJA1 Gap junction alpha-1 protein | Membrane | 126 | 43494 | 5 | 7.9 |
| IPI00297160 | P16070 | Tax_Id=9606 Gene_Symbol=CD44 Isoform 12 of CD44 antigen | Membrane | 110 | 39904 | 5 | 5.5 |
| IPI00013508 | P12814 | Tax_Id=9606 Gene_Symbol=ACTN1 Alpha-actinin-1 | Membrane | 307 | 103563 | 4 | 5.6 |
| IPI00000190 | P60033 | Tax_Id=9606 Gene_Symbol=CD81 CD81 antigen | Membrane | 276 | 26476 | 4 | 25 |
| IPI00783987 | P01024 | Tax_Id=9606 Gene_Symbol=C3 Complement C3 (Fragment) | Membrane | 241 | 188569 | 4 | 2.9 |
| IPI00298994 | Q9Y490 | Tax_Id=9606 Gene_Symbol=TLN1 Talin-1 | Membrane | 189 | 271766 | 4 | 1.8 |
| IPI00025252 | P30101 | Tax_Id=9606 Gene_Symbol=PDIA3 Protein disulfide-isomerase A3 | Membrane | 151 | 57146 | 4 | 7.1 |
| IPI00251454 | Q8N2A8 | Tax_Id=9606 Gene_Symbol=PLD6 Phospholipase D6 | Membrane | 54 | 28654 | 4 | 9.9 |
| IPI00022827 | Q9H2G2 | Tax_Id=9606 Gene_Symbol=SLK Isoform 1 of STE20-like serine/threonine-protein kinase | Membrane | 48 | 143234 | 4 | 1.5 |
| IPI00015473 | P43003 | Tax_Id=9606 Gene_Symbol=SLC1A3 Excitatory amino acid transporter 1 | Membrane | 224 | 59705 | 3 | 7.7 |
| IPI00012303 | Q13228 | Tax_Id=9606 Gene_Symbol=SELENBP1 Selenium binding protein 1 | Membrane | 215 | 57457 | 3 | 10.9 |
| IPI00016461 | P51178 | Tax_Id=9606 Gene_Symbol=PLCD1 phospholipase C, delta 1 isoform 1 | Membrane | 156 | 88878 | 3 | 5.3 |
| IPI00217871 | P30038 | Tax_Id=9606 Gene_Symbol=ALDH4A1 Delta-1-pyrroline-5-carboxylate dehydrogenase, mitochondrial | Membrane | 91 | 62137 | 3 | 7.1 |
| IPI00020599 | P27797 | Tax_Id=9606 Gene_Symbol=CALR Calreticulin | Membrane | 72 | 48283 | 3 | 4.1 |
| IPI00027626 | P40227 | Tax_Id=9606 Gene_Symbol=CCT6A T-complex protein 1 subunit zeta | Membrane | 69 | 58444 | 3 | 5.3 |
| IPI00289758 | P17655 | Tax_Id=9606 Gene_Symbol=CAPN2 Calpain-2 catalytic subunit | Membrane | 176 | 80814 | 2 | 6.4 |
| IPI00018206 | P00505 | Tax_Id=9606 Gene_Symbol=GOT2 Aspartate aminotransferase, mitochondrial | Membrane | 133 | 47844 | 2 | 8.1 |
| IPI00747849 | P05026 | Tax_Id=9606 Gene_Symbol=ATP1B1 Isoform 1 of Sodium/potassium-transporting ATPase subunit beta-1 | Membrane | 132 | 35438 | 2 | 8.9 |
| IPI00291175 | P18206 | Tax_Id=9606 Gene_Symbol=VCL Isoform 1 of Vinculin | Membrane | 125 | 117220 | 2 | 2.7 |
| IPI00554711 | P14923 | Tax_Id=9606 Gene_Symbol=JUP Junction plakoglobin | Membrane | 94 | 82434 | 2 | 3 |
| IPI00022202 | Q00325 | Tax_Id=9606 Gene_Symbol=SLC25A3 Isoform A of Phosphate carrier protein, mitochondrial | Membrane | 89 | 40525 | 2 | 5.2 |
| IPI00930224 | Q5TCS8 | Tax_Id=9606 Gene_Symbol=AKD1 adenylate kinase domain containing 1 isoform 1 | Membrane | 58 | 222698 | 2 | 1.3 |
| IPI00019952 | P51674 | Tax_Id=9606 Gene_Symbol=GPM6A Neuronal membrane glycoprotein M6-a | Membrane | 57 | 31930 | 2 | 4 |
| IPI00293971 | P14415 | Tax_Id=9606 Gene_Symbol=ATP1B2 Sodium/potassium-transporting ATPase subunit beta-2 | Membrane | 55 | 33745 | 2 | 7.2 |
| IPI00294556 | Q02410 | Tax_Id=9606 Gene_Symbol=APBA1 Amyloid beta A4 precursor protein-binding family A member 1 | Membrane | 46 | 93263 | 2 | 2.2 |
| IPI00219315 | P42263 | Tax_Id=9606 Gene_Symbol=GRIA3 Isoform Flip of Glutamate receptor 3 | Membrane | 43 | 101733 | 2 | 3 |
| IPI00470809 | Q68CR1 | Tax_Id=9606 Gene_Symbol=SEL1L3 Isoform 1 of Protein sel-1 homolog 3 | Membrane | 41 | 129512 | 2 | 1.4 |
| IPI00004573 | P01833 | Tax_Id=9606 Gene_Symbol=PIGR Polymeric immunoglobulin receptor | Membrane | 106 | 84429 | 1 | 2.1 |
| IPI00024689 | P29972 | Tax_Id=9606 Gene_Symbol=AQP1 Aquaporin-1 | Membrane | 95 | 28736 | 1 | 5.6 |
| IPI00291006 | P40926 | Tax_Id=9606 Gene_Symbol=MDH2 Malate dehydrogenase, mitochondrial | Membrane | 90 | 35937 | 1 | 5.6 |
| IPI00011285 | P07384 | Tax_Id=9606 Gene_Symbol=CAPN1 Calpain-1 catalytic subunit | Membrane | 87 | 82465 | 1 | 2.7 |
| IPI00300020 | P43004 | Tax_Id=9606 Gene_Symbol=SLC1A2 Isoform 1 of Excitatory amino acid transporter 2 | Membrane | 86 | 62577 | 1 | 2.6 |
| IPI00031522 | P40939 | Tax_Id=9606 Gene_Symbol=HADHA Trifunctional enzyme subunit alpha, mitochondrial | Membrane | 82 | 83688 | 1 | 2.4 |
| IPI00016949 | Q9Y6R1 | Tax_Id=9606 Gene_Symbol=SLC4A4 Isoform 4 of Electrogenic sodium bicarbonate cotransporter 1 | Membrane | 73 | 113055 | 1 | 2.4 |
| IPI00658109 | P12532 | Tax_Id=9606 Gene_Symbol=CKMT1B;CKMT1A Isoform 1 of Creatine kinase U-type, mitochondrial | Membrane | 63 | 47406 | 1 | 5 |
| IPI00218646 | P04839 | Tax_Id=9606 Gene_Symbol=CYBB Cytochrome b-245 heavy chain | Membrane | 60 | 66206 | 1 | 3 |
| IPI00290085 | P19022 | Tax_Id=9606 Gene_Symbol=CDH2 Cadherin-2 | Membrane | 59 | 100203 | 1 | 2.9 |
| IPI00019901 | P35611 | Tax_Id=9606 Gene_Symbol=ADD1 Isoform 1 of Alpha-adducin | Membrane | 54 | 81304 | 1 | 3.7 |
| IPI00384998 | O94856 | Tax_Id=9606 Gene_Symbol=NFASC Isoform 7 of Neurofascin | Membrane | 46 | 140650 | 1 | 1.4 |
| IPI00025874 | P04843 | Tax_Id=9606 Gene_Symbol=RPN1 Dolichyl-diphosphooligosaccharide--protein glycosyltransferase subunit 1 precursor | Membrane | 43 | 72847 | 1 | 2.3 |
| IPI00019906 | P35613 | Tax_Id=9606 Gene_Symbol=BSG Isoform 2 of Basigin | Membrane | 42 | 29431 | 1 | 6.7 |
| IPI00337541 | Q13423 | Tax_Id=9606 Gene_Symbol=NNT NAD(P) transhydrogenase, mitochondrial | Membrane | 39 | 114564 | 1 | 0.9 |
| IPI00032258 | P0C0L4 | Tax_Id=9606 Gene_Symbol=C4A Complement C4-A | Membrane | 37 | 194247 | 1 | 1.4 |
| IPI00305383 | P22695 | Tax_Id=9606 Gene_Symbol=UQCRC2 Cytochrome b-c1 complex subunit 2, mitochondrial | Membrane | 37 | 48584 | 1 | 4.4 |
| IPI00183879 | Q3MIP1 | Tax_Id=9606 Gene_Symbol=ITPRIPL2 Inositol 1,4,5-triphosphate receptor-interacting protein-like 2 | Membrane | 36 | 59379 | 1 | 2.6 |
| IPI00032405 | O60883 | Tax_Id=9606 Gene_Symbol=GPR37L1 Endothelin B receptor-like protein 2 | Membrane | 35 | 54162 | 1 | 1.7 |
| IPI00025363 | P14136 | Tax_Id=9606 Gene_Symbol=GFAP Isoform 1 of Glial fibrillary acidic protein | Cytoplasm | 2865 | 49907 | 735 | 77.8 |
| IPI00745872 | P02768 | Tax_Id=9606 Gene_Symbol=ALB Isoform 1 of Serum albumin | Cytoplasm | 2630 | 71317 | 142 | 75.4 |
| IPI00871535 | Q13813 | Tax_Id=9606 Gene_Symbol=SPTAN1 Isoform 2 of Spectrin alpha chain, brain | Cytoplasm | 4345 | 285717 | 99 | 38.7 |
| IPI00843765 | Q13813 | Tax_Id=9606 Gene_Symbol=SPTAN1 Isoform 3 of Spectrin alpha chain, brain | Cytoplasm | 4285 | 282906 | 98 | 38.6 |
| IPI00021439 | P60709 | Tax_Id=9606 Gene_Symbol=ACTB Actin, cytoplasmic 1 | Cytoplasm | 1229 | 42052 | 83 | 70.9 |
| IPI00005614 | Q01082 | Tax_Id=9606 Gene_Symbol=SPTBN1 Isoform Long of Spectrin beta chain, brain 1 | Cytoplasm | 2643 | 275237 | 78 | 21.9 |
| IPI00418471 | P08670 | Tax_Id=9606 Gene_Symbol=VIM Vimentin | Cytoplasm | 1391 | 53676 | 62 | 51.5 |
| IPI00792677 | B4DDU2 | Tax_Id=9606 Gene_Symbol=TUBA1B cDNA FLJ60097, highly similar to Tubulin alpha-ubiquitous chain | Cytoplasm | 1271 | 46797 | 57 | 62.5 |
| IPI00180675 | Q71U36 | Tax_Id=9606 Gene_Symbol=TUBA1A Tubulin alpha-1A chain | Cytoplasm | 1258 | 50788 | 57 | 57.6 |
| IPI00465248 | P06733 | Tax_Id=9606 Gene_Symbol=ENO1 Isoform alpha-enolase of Alpha-enolase | Cytoplasm | 1502 | 47481 | 49 | 60.1 |
| IPI00479186 | P14618 | Tax_Id=9606 Gene_Symbol=PKM2 Isoform M2 of Pyruvate kinase isozymes M1/M2 | Cytoplasm | 1629 | 58470 | 47 | 55.9 |
| IPI00398625 | Q86YZ3 | Tax_Id=9606 Gene_Symbol=HRNR Hornerin | Cytoplasm | 1401 | 283140 | 46 | 10.8 |
| IPI00003269 | Q562R1 | Tax_Id=9606 Gene_Symbol=ACTBL2 Beta-actin-like protein 2 | Cytoplasm | 321 | 42318 | 46 | 17.6 |
| IPI00007750 | P68366 | Tax_Id=9606 Gene_Symbol=TUBA4A Tubulin alpha-4A chain | Cytoplasm | 1046 | 50634 | 44 | 49.3 |
| IPI00013475 | Q13885 | Tax_Id=9606 Gene_Symbol=TUBB2A Tubulin beta-2A chain | Cytoplasm | 1395 | 50274 | 42 | 60.7 |
| IPI00031370 | Q9BVA1 | Tax_Id=9606 Gene_Symbol=TUBB2B Tubulin beta-2B chain | Cytoplasm | 1358 | 50377 | 42 | 60.7 |
| IPI00257508 | Q16555 | Tax_Id=9606 Gene_Symbol=DPYSL2 Dihydropyrimidinase-related protein 2 | Cytoplasm | 1604 | 62711 | 41 | 59.4 |
| IPI00021428 | P68133 | Tax_Id=9606 Gene_Symbol=ACTA1 Actin, alpha skeletal muscle | Cytoplasm | 551 | 42366 | 40 | 26.8 |
| IPI00219018 | P04406 | Tax_Id=9606 Gene_Symbol=GAPDH Glyceraldehyde-3-phosphate dehydrogenase | Cytoplasm | 1100 | 36201 | 39 | 63.3 |
| IPI00003021 | P50993 | Tax_Id=9606 Gene_Symbol=ATP1A2 Sodium/potassium-transporting ATPase subunit alpha-2 | Cytoplasm | 1493 | 113505 | 38 | 30.3 |
| IPI00909140 | P07436 | Tax_Id=9606 Gene_Symbol=TUBB Tubulin beta chain | Cytoplasm | 1181 | 50095 | 38 | 53.6 |
| IPI00072917 | P12111 | Tax_Id=9606 Gene_Symbol=COL6A3 COL6A3 protein | Cytoplasm | 1590 | 323455 | 35 | 11.9 |
| IPI00954527 | Q13707 | Tax_Id=9606 Gene_Symbol=ACTA2 ACTA2 protein (Fragment) | Cytoplasm | 452 | 37125 | 35 | 25.2 |
| IPI00003865 | P11142 | Tax_Id=9606 Gene_Symbol=HSPA8 Isoform 1 of Heat shock cognate 71 kDa protein | Cytoplasm | 1137 | 71082 | 34 | 36.8 |
| IPI00023598 | P04350 | Tax_Id=9606 Gene_Symbol=TUBB4 Tubulin beta-4 chain | Cytoplasm | 1008 | 50010 | 33 | 48.6 |
| IPI00013683 | Q13509 | Tax_Id=9606 Gene_Symbol=TUBB3 Tubulin beta-3 chain | Cytoplasm | 1024 | 50856 | 32 | 43.1 |
| IPI00398002 | Q15149 | Tax_Id=9606 Gene_Symbol=PLEC1 Isoform 3 of Plectin-1 | Cytoplasm | 1192 | 519655 | 31 | 6.3 |
| IPI00237671 | P07196 | Tax_Id=9606 Gene_Symbol=NEFL Neurofilament light polypeptide | Cytoplasm | 602 | 61536 | 30 | 19.7 |
| IPI00029111 | Q14195 | Tax_Id=9606 Gene_Symbol=DPYSL3 Collapsin response mediator protein 4 long variant | Cytoplasm | 1094 | 74321 | 27 | 32.5 |
| IPI00007702 | P54652 | Tax_Id=9606 Gene_Symbol=HSPA2 Heat shock-related 70 kDa protein 2 | Cytoplasm | 793 | 70263 | 27 | 24.3 |
| IPI00302840 | P13637 | Tax_Id=9606 Gene_Symbol=ATP1A3 Sodium/potassium-transporting ATPase subunit alpha-3 | Cytoplasm | 1102 | 113102 | 23 | 22.2 |
| IPI00304925 | P08107 | Tax_Id=9606 Gene_Symbol=HSPA1A;HSPA1B Heat shock 70 kDa protein 1A/1B | Cytoplasm | 876 | 70294 | 23 | 29.6 |
| IPI00006482 | P05023 | Tax_Id=9606 Gene_Symbol=ATP1A1 Isoform Long of Sodium/potassium-transporting ATPase subunit alpha-1 | Cytoplasm | 1027 | 114135 | 22 | 20.8 |
| IPI00022977 | P12277 | Tax_Id=9606 Gene_Symbol=CKB Creatine kinase B-type | Cytoplasm | 663 | 42902 | 21 | 36.2 |
| IPI00923396 | P12036 | Tax_Id=9606 Gene_Symbol=NEFH Isoform 2 of Neurofilament heavy polypeptide | Cytoplasm | 586 | 105804 | 21 | 11.3 |
| IPI00010154 | P31150 | Tax_Id=9606 Gene_Symbol=GDI1 Rab GDP dissociation inhibitor alpha | Cytoplasm | 970 | 51177 | 20 | 42.5 |
| IPI00303476 | P06576 | Tax_Id=9606 Gene_Symbol=ATP5B ATP synthase subunit beta, mitochondrial | Cytoplasm | 884 | 56525 | 19 | 40.5 |
| IPI00382470 | P07900 | Tax_Id=9606 Gene_Symbol=HSP90AA1 Isoform 2 of Heat shock protein HSP 90-alpha | Cytoplasm | 774 | 98670 | 19 | 19.4 |
| IPI00026314 | P06396 | Tax_Id=9606 Gene_Symbol=GSN Isoform 1 of Gelsolin | Cytoplasm | 692 | 86043 | 19 | 26.6 |
| IPI00177728 | Q96KP4 | Tax_Id=9606 Gene_Symbol=CNDP2 Isoform 1 of Cytosolic non-specific dipeptidase | Cytoplasm | 626 | 53187 | 15 | 31.4 |
| IPI00221234 | P49419 | Tax_Id=9606 Gene_Symbol=ALDH7A1 aldehyde dehydrogenase 7 family, member A1 | Cytoplasm | 548 | 59020 | 14 | 28.4 |
| IPI00216171 | P09104 | Tax_Id=9606 Gene_Symbol=ENO2 Gamma-enolase | Cytoplasm | 570 | 47581 | 13 | 24.2 |
| IPI00217507 | P07197 | Tax_Id=9606 Gene_Symbol=NEFM Neurofilament medium polypeptide | Cytoplasm | 429 | 102468 | 13 | 8.7 |
| IPI00553177 | P01009 | Tax_Id=9606 Gene_Symbol=SERPINA1 Isoform 1 of Alpha-1-antitrypsin | Cytoplasm | 441 | 46878 | 12 | 29.2 |
| IPI00169383 | P00558 | Tax_Id=9606 Gene_Symbol=PGK1 Phosphoglycerate kinase 1 | Cytoplasm | 479 | 44985 | 11 | 30 |
| IPI00414676 | P08238 | Tax_Id=9606 Gene_Symbol=HSP90AB1 Heat shock protein HSP 90-beta | Cytoplasm | 454 | 83554 | 11 | 11.7 |
| IPI00465439 | P04075 | Tax_Id=9606 Gene_Symbol=ALDOA Fructose-bisphosphate aldolase A | Cytoplasm | 433 | 39851 | 11 | 33 |
| IPI00418262 | P09972 | Tax_Id=9606 Gene_Symbol=ALDOC Fructose-bisphosphate aldolase | Cytoplasm | 427 | 49062 | 10 | 26.2 |
| IPI00220737 | P13591 | Tax_Id=9606 Gene_Symbol=NCAM1 Isoform 3 of Neural cell adhesion molecule 1 | Cytoplasm | 521 | 84345 | 9 | 15.9 |
| IPI00003362 | P11021 | Tax_Id=9606 Gene_Symbol=HSPA5 HSPA5 protein | Cytoplasm | 408 | 72492 | 9 | 13 |
| IPI00027230 | P14625 | Tax_Id=9606 Gene_Symbol=HSP90B1 Endoplasmin | Cytoplasm | 375 | 92696 | 9 | 8.8 |
| IPI00289862 | Q12765 | Tax_Id=9606 Gene_Symbol=SCRN1 Secernin-1 | Cytoplasm | 337 | 46980 | 8 | 18.6 |
| IPI00221226 | P08133 | Tax_Id=9606 Gene_Symbol=ANXA6 Annexin A6 | Cytoplasm | 519 | 76168 | 7 | 17.8 |
| IPI00328156 | P27338 | Tax_Id=9606 Gene_Symbol=MAOB Amine oxidase [flavin-containing] B | Cytoplasm | 349 | 59238 | 7 | 11.2 |
| IPI00294187 | Q9Y2J8 | Tax_Id=9606 Gene_Symbol=PADI2 Protein-arginine deiminase type-2 | Cytoplasm | 311 | 76257 | 7 | 9.8 |
| IPI00784154 | P10809 | Tax_Id=9606 Gene_Symbol=HSPD1 60 kDa heat shock protein, mitochondrial | Cytoplasm | 241 | 61187 | 7 | 11.7 |
| IPI00008868 | P46821 | Tax_Id=9606 Gene_Symbol=MAP1B Microtubule-associated protein 1B | Cytoplasm | 143 | 271651 | 6 | 1.7 |
| IPI00026216 | P55786 | Tax_Id=9606 Gene_Symbol=NPEPPS Puromycin-sensitive aminopeptidase | Cytoplasm | 349 | 103895 | 5 | 8.4 |
| IPI00004358 | P11216 | Tax_Id=9606 Gene_Symbol=PYGB Glycogen phosphorylase, brain form | Cytoplasm | 282 | 97319 | 5 | 10 |
| IPI00908881 | B4DVJ0 | Tax_Id=9606 Gene_Symbol=GPI Glucose-6-phosphate isomerase | Cytoplasm | 265 | 60398 | 5 | 10.2 |
| IPI00019502 | P35579 | Tax_Id=9606 Gene_Symbol=MYH9 Isoform 1 of Myosin-9 | Cytoplasm | 236 | 227646 | 5 | 4 |
| IPI00217182 | P15924 | Tax_Id=9606 Gene_Symbol=DSP Isoform DPII of Desmoplakin | Cytoplasm | 191 | 262237 | 5 | 3.2 |
| IPI00419237 | P28838 | Tax_Id=9606 Gene_Symbol=LAP3 Isoform 1 of Cytosol aminopeptidase | Cytoplasm | 180 | 56530 | 5 | 8.7 |
| IPI00007682 | P38606 | Tax_Id=9606 Gene_Symbol=ATP6V1A V-type proton ATPase catalytic subunit A | Cytoplasm | 179 | 68660 | 4 | 7.6 |
| IPI00011200 | O43175 | Tax_Id=9606 Gene_Symbol=PHGDH D-3-phosphoglycerate dehydrogenase | Cytoplasm | 267 | 57356 | 3 | 9 |
| IPI00456969 | Q14204 | Tax_Id=9606 Gene_Symbol=DYNC1H1 Cytoplasmic dynein 1 heavy chain 1 | Cytoplasm | 259 | 534809 | 3 | 1.1 |
| IPI00217966 | P00338 | Tax_Id=9606 Gene_Symbol=LDHA Isoform 1 of L-lactate dehydrogenase A chain | Cytoplasm | 220 | 36950 | 3 | 8.7 |
| IPI00479877 | P49189 | Tax_Id=9606 Gene_Symbol=ALDH9A1 aldehyde dehydrogenase 9A1 | Cytoplasm | 124 | 57168 | 3 | 6.9 |
| IPI00657839 | Q9NXG0 | Tax_Id=9606 Gene_Symbol=CNTLN Isoform 2 of Centlein | Cytoplasm | 58 | 162673 | 3 | 1.1 |
| IPI00022300 | Q9H8H3 | Tax_Id=9606 Gene_Symbol=METTL7A Methyltransferase-like protein 7A | Cytoplasm | 174 | 28814 | 2 | 11.1 |
| IPI00008274 | Q01518 | Tax_Id=9606 Gene_Symbol=CAP1 Isoform 1 of Adenylyl cyclase-associated protein 1 | Cytoplasm | 108 | 52222 | 2 | 3.8 |
| IPI00413641 | P15121 | Tax_Id=9606 Gene_Symbol=AKR1B1 Aldose reductase | Cytoplasm | 99 | 36230 | 2 | 17.1 |
| IPI00171791 | Q8N4C6 | Tax_Id=9606 Gene_Symbol=NIN Isoform 3 of Ninein | Cytoplasm | 72 | 242852 | 2 | 0.8 |
| IPI00295777 | P21695 | Tax_Id=9606 Gene_Symbol=GPD1 Glycerol-3-phosphate dehydrogenase [NAD+], cytoplasmic | Cytoplasm | 57 | 38171 | 2 | 9.2 |
| IPI00395633 | O14830 | Tax_Id=9606 Gene_Symbol=PPEF2 Isoform PPEF-2(S) of Serine/threonine-protein phosphatase with EF-hands 2 | Cytoplasm | 46 | 69712 | 2 | 2.8 |
| IPI00220156 | P61812 | Tax_Id=9606 Gene_Symbol=TGFB2 Isoform B of Transforming growth factor beta-2 | Cytoplasm | 43 | 51567 | 2 | 4.8 |
| IPI00030268 | Q5H9T9 | Tax_Id=9606 Gene_Symbol=FSCB Fibrous sheath CABYR-binding protein | Cytoplasm | 42 | 89134 | 2 | 2.3 |
| IPI00010133 | P31146 | Tax_Id=9606 Gene_Symbol=CORO1A Coronin-1A | Cytoplasm | 80 | 51678 | 1 | 3.5 |
| IPI00003168 | O60256 | Tax_Id=9606 Gene_Symbol=PRPSAP2 Phosphoribosyl pyrophosphate synthetase-associated protein 2 | Cytoplasm | 71 | 41299 | 1 | 6.2 |
| IPI00022429 | P02763 | Tax_Id=9606 Gene_Symbol=ORM1 Alpha-1-acid glycoprotein 1 | Cytoplasm | 69 | 23725 | 1 | 7 |
| IPI00218914 | P00352 | Tax_Id=9606 Gene_Symbol=ALDH1A1 Retinal dehydrogenase 1 | Cytoplasm | 64 | 55454 | 1 | 2.6 |
| IPI00011454 | Q14697 | Tax_Id=9606 Gene_Symbol=GANAB Isoform 2 of Neutral alpha-glucosidase AB | Cytoplasm | 54 | 109825 | 1 | 2.4 |
| IPI00008994 | Q9UN36 | Tax_Id=9606 Gene_Symbol=NDRG2 Isoform 1 of Protein NDRG2 | Cytoplasm | 52 | 41114 | 1 | 5.7 |
| IPI00101645 | Q96HN2 | Tax_Id=9606 Gene_Symbol=AHCYL2 Putative adenosylhomocysteinase 3 | Cytoplasm | 52 | 67705 | 1 | 1.8 |
| IPI00037448 | Q9UBQ7 | Tax_Id=9606 Gene_Symbol=GRHPR Glyoxylate reductase/hydroxypyruvate reductase | Cytoplasm | 50 | 36045 | 1 | 7.6 |
| IPI00027341 | P40121 | Tax_Id=9606 Gene_Symbol=CAPG Macrophage-capping protein | Cytoplasm | 49 | 38779 | 1 | 8 |
| IPI00022082 | Q92599 | Tax_Id=9606 Gene_Symbol=SEPT8 Isoform 2 of Septin-8 | Cytoplasm | 47 | 50068 | 1 | 5.6 |
| IPI00219029 | P17174 | Tax_Id=9606 Gene_Symbol=GOT1 Aspartate aminotransferase, cytoplasmic | Cytoplasm | 42 | 46447 | 1 | 4.8 |
| IPI00291419 | Q9BWD1 | Tax_Id=9606 Gene_Symbol=ACAT2 cDNA FLJ53975, highly similar to Acetyl-CoA acetyltransferase, cytosolic | Cytoplasm | 42 | 45184 | 1 | 6.1 |
| IPI00033419 | Q9UK73 | Tax_Id=9606 Gene_Symbol=FEM1B Protein fem-1 homolog B | Cytoplasm | 41 | 71189 | 1 | 1.3 |
| IPI00915869 | B9A041 | Tax_Id=9606 Gene_Symbol=MDH1 Malate dehydrogenase | Cytoplasm | 40 | 23195 | 1 | 4.8 |
| IPI00007087 |  | Tax_Id=9606 Gene_Symbol=FBXO2 F-box only protein 2 | Cytoplasm | 37 | 33706 | 1 | 6.4 |
| IPI00300376 | Q08188 | Tax_Id=9606 Gene_Symbol=TGM3 Protein-glutamine gamma-glutamyltransferase E | Cytoplasm | 36 | 76926 | 1 | 1.3 |
| IPI00746777 | P11766 | Tax_Id=9606 Gene_Symbol=ADH5P4;ADH5 Alcohol dehydrogenase class-3 | Cytoplasm | 34 | 40554 | 1 | 2.1 |
| IPI00007752 | P68371 | Tax_Id=9606 Gene_Symbol=TUBB2C Tubulin beta-2C chain | Nucleus | 1336 | 50255 | 47 | 60.7 |
| IPI00643920 | P29401 | Tax_Id=9606 Gene_Symbol=TKT cDNA FLJ54957, highly similar to Transketolase | Nucleus | 819 | 69382 | 19 | 32.6 |
| IPI00409658 | O95935 | Tax_Id=9606 Gene_Symbol=TBX18 T-box transcription factor TBX18 | Nucleus | 52 | 65225 | 12 | 3.8 |
| IPI00645078 | P22314 | Tax_Id=9606 Gene_Symbol=UBA1 Ubiquitin-like modifier-activating enzyme 1 | Nucleus | 636 | 118858 | 10 | 13.5 |
| IPI00431645 | Q6NSB4 | Tax_Id=9606 Gene_Symbol=HP HP protein | Nucleus | 267 | 31647 | 5 | 24.6 |
| IPI00100160 | Q86VP6 | Tax_Id=9606 Gene_Symbol=CAND1 Isoform 1 of Cullin-associated NEDD8-dissociated protein 1 | Nucleus | 217 | 137999 | 5 | 5.8 |
| IPI00154528 | Q96SB8 | Tax_Id=9606 Gene_Symbol=SMC6 Isoform 1 of Structural maintenance of chromosomes protein 6 | Nucleus | 106 | 127216 | 4 | 3.4 |
| IPI00028565 | P32456 | Tax_Id=9606 Gene_Symbol=GBP2 Interferon-induced guanylate-binding protein 2 | Nucleus | 48 | 67680 | 3 | 2.5 |
| IPI00024024 | Q02040 | Tax_Id=9606 Gene_Symbol=SFRS17A Isoform 1 of Splicing factor, arginine/serine-rich 17A | Nucleus | 47 | 81313 | 3 | 3.3 |
| IPI00018321 | Q15047 | Tax_Id=9606 Gene_Symbol=SETDB1 Isoform 1 of Histone-lysine N-methyltransferase SETDB1 | Nucleus | 40 | 145119 | 3 | 1.1 |
| IPI00179330 | P62979 | Tax_Id=9606 Gene_Symbol=UBC;RPS27A;UBB ubiquitin and ribosomal protein S27a precursor | Nucleus | 63 | 18296 | 2 | 10.3 |
| IPI00007277 | Q9Y608 | Tax_Id=9606 Gene_Symbol=LRRFIP2 Isoform 1 of Leucine-rich repeat flightless-interacting protein 2 | Nucleus | 46 | 82349 | 2 | 2.1 |
| IPI00217669 | P49917 | Tax_Id=9606 Gene_Symbol=LIG4 DNA ligase 4 | Nucleus | 100 | 105044 | 1 | 3 |
| IPI00026268 | P62873 | Tax_Id=9606 Gene_Symbol=GNB1 Guanine nucleotide-binding protein G(I)/G(S)/G(T) subunit beta-1 | Nucleus | 99 | 38151 | 1 | 5.3 |
| IPI00424869 | Q6U7G8 | Tax_Id=9606 Gene_Symbol=RAP1GDS1 RAP1, GTP-GDP dissociation stimulator 1 isoform 6 | Nucleus | 72 | 57070 | 1 | 3.7 |
| IPI00793443 | O00410 | Tax_Id=9606 Gene_Symbol=IPO5 Isoform 1 of Importin-5 | Nucleus | 67 | 125032 | 1 | 1.8 |
| IPI00014424 | Q05639 | Tax_Id=9606 Gene_Symbol=EEF1A2 Elongation factor 1-alpha 2 | Nucleus | 61 | 50780 | 1 | 6.3 |
| IPI00176903 | Q6NZI2 | Tax_Id=9606 Gene_Symbol=PTRF Isoform 1 of Polymerase I and transcript release factor | Nucleus | 57 | 43450 | 1 | 2.8 |
| IPI00007244 | P05164 | Tax_Id=9606 Gene_Symbol=MPO Isoform H17 of Myeloperoxidase | Nucleus | 54 | 84784 | 1 | 2.7 |
| IPI00021405 | P02545 | Tax_Id=9606 Gene_Symbol=LMNA Isoform A of Lamin-A/C | Nucleus | 48 | 74380 | 1 | 2.4 |
| IPI00020194 | Q92804 | Tax_Id=9606 Gene_Symbol=TAF15 Isoform Short of TATA-binding protein-associated factor 2N | Nucleus | 35 | 61749 | 1 | 1.7 |
|  |  |  |  |  |  |  |  |
| IPI00291136 | P12109 | Tax_Id=9606 Gene_Symbol=COL6A1 Collagen alpha-1(VI) chain | Secreted | 289 | 109602 | 11 | 9.6 |
| IPI00020987 | P51888 | Tax_Id=9606 Gene_Symbol=PRELP Prolargin | Secreted | 168 | 44181 | 8 | 8.1 |
| IPI00010790 | P21810 | Tax_Id=9606 Gene_Symbol=BGN Biglycan | Secreted | 201 | 42027 | 7 | 10.6 |
| IPI00297646 | Q14042 | Tax_Id=9606 Gene_Symbol=COL1A1 Collagen alpha-1(I) chain | Secreted | 260 | 139853 | 6 | 3.5 |
| IPI00022463 | P02787 | Tax_Id=9606 Gene_Symbol=TF Serotransferrin | Secreted | 309 | 79280 | 5 | 8.6 |
| IPI00160552 | Q92752 | Tax_Id=9606 Gene_Symbol=TNR Isoform 1 of Tenascin-R | Secreted | 259 | 151791 | 4 | 4.5 |
| IPI00298497 | P02675 | Tax_Id=9606 Gene_Symbol=FGB Fibrinogen beta chain | Secreted | 134 | 56577 | 3 | 5.7 |
| IPI00019038 | P61626 | Tax_Id=9606 Gene_Symbol=LYZ Lysozyme C | Secreted | 103 | 16982 | 2 | 14.2 |
| IPI00012119 | P07585 | Tax_Id=9606 Gene_Symbol=DCN Isoform A of Decorin | Secreted | 58 | 40064 | 2 | 5.3 |
| IPI00176125 | A8TX70 | Tax_Id=9606 Gene_Symbol=COL29A1 Isoform 1 of Collagen alpha-5(VI) chain | Secreted | 49 | 291796 | 2 | 0.6 |
| IPI00555812 | P02774 | Tax_Id=9606 Gene_Symbol=GC Isoform 1 of Vitamin D-binding protein | Secreted | 48 | 54526 | 2 | 4.6 |
| IPI00028908 | Q14112 | Tax_Id=9606 Gene_Symbol=NID2 Nidogen-2 | Secreted | 72 | 154093 | 1 | 2.1 |
| IPI00017601 | P00450 | Tax_Id=9606 Gene_Symbol=CP Ceruloplasmin | Secreted | 56 | 122983 | 1 | 2.2 |
| IPI00176193 | Q05707 | Tax_Id=9606 Gene_Symbol=COL14A1 Isoform 1 of Collagen alpha-1(XIV) chain | Secreted | 55 | 194478 | 1 | 0.6 |
| IPI00024621 | Q9NRN5 | Tax_Id=9606 Gene_Symbol=OLFML3 Isoform 1 of Olfactomedin-like protein 3 | Secreted | 53 | 46380 | 1 | 2.5 |
| IPI00292836 | Q9P1Z9 | Tax_Id=9606 Gene_Symbol=KIAA1529 Isoform 1 of Unknown protein KIAA1529 | Unknown | 37 | 192404 | 36 | 1.2 |
| IPI00553169 | A6NDY9 | Tax_Id=9606 Gene_Symbol=FLNA Putative Unknown protein FLNA | Unknown | 865 | 248149 | 16 | 9.2 |
| IPI00384938 | Q7Z351 | Tax_Id=9606 Gene_Symbol=IGHV4-31;LOC100290320;LOC100294459;IGHG1 Putative Unknown protein DKFZp686N02209 | Unknown | 379 | 53503 | 11 | 20.5 |
| IPI00738806 | C9J6C0 | Tax_Id=9606 Gene_Symbol=MYO7B Putative Unknown protein MYO7B | Unknown | 58 | 243711 | 8 | 0.9 |
| IPI00550991 | P01011 | Tax_Id=9606 Gene_Symbol=SERPINA3 cDNA FLJ35730 fis, clone TESTI2003131, highly similar to ALPHA-1-ANTICHYMOTRYPSIN | Unknown | 256 | 50737 | 6 | 10.7 |
| IPI00872463 |  | Tax_Id=9606 Gene_Symbol=TUBAL3 50 kDa protein | Unknown | 164 | 50674 | 6 | 13.7 |
| IPI00013290 | Q7Z4V5 | Tax_Id=9606 Gene_Symbol=HDGF2 75 kDa protein | Unknown | 74 | 74943 | 5 | 2.4 |
| IPI00789324 | F5GWP8 | Tax_Id=9606 Gene_Symbol=JUP cDNA FLJ60424, highly similar to Junction plakoglobin | Unknown | 118 | 62862 | 3 | 3.7 |
| IPI00020984 | B4DGP8 | Tax_Id=9606 Gene_Symbol=CANX cDNA FLJ55574, highly similar to Calnexin | Unknown | 106 | 71971 | 2 | 2.6 |
| IPI00871279 |  | Tax_Id=9606 Gene_Symbol=SLC38A1 Putative Unknown protein SLC38A1 | Unknown | 45 | 59471 | 2 | 3.4 |
| IPI00878584 |  | Tax_Id=9606 Gene_Symbol=- 40 kDa protein | Unknown | 44 | 40650 | 2 | 4.1 |
| IPI00555809 | Q562S5 | Tax_Id=9606 Gene_Symbol=- Pseudogene candidate | Unknown | 73 | 26180 | 1 | 10.9 |
| IPI00025465 | P20774 | Tax_Id=9606 Gene_Symbol=OGN cDNA FLJ59205, highly similar to Mimecan | Unknown | 70 | 40870 | 1 | 4.5 |
| IPI00033025 | E7EPK1 | Tax_Id=9606 Gene_Symbol=SEPT7 51 kDa protein | Unknown | 70 | 50820 | 1 | 5.3 |

| **Gene name** | **Primers** | **Amplicon size** |
| --- | --- | --- |
| **EphrinA1** | F: 5′-atcccaagttccgagaggagg  R: 5′-ctccttgcccaaggtgaaaggc | 251 |
| **EphrinA2** | F: 5′-ctataccgtggaggtgagca  R: 5′-caggtgctccaaccctccac | 300 |
| **EphrinA3** | F: 5′-tcgccttcttcctcatgacg  R: 5′-ctgagcactgcctttatagcc | 274 |
| **EphrinA4** | F: 5′-gagctgggcttcaacgatta  R: 5′-tgacttggaaggtgtgcttg | 550 |
| **EphrinA5** | F: 5′-aacggaccgctgaagttctcgg  R: 5′-tttgtgccgcgttctctccgcg | 273 |
| **EphrinB1** | F: 5′-agctgcttgcagcactgtgc  R: 5′-ctcatgcttgccatcagagtc | 350 |
| **EphrinB2** | F: 5′-accgctaaggactgcagacag  R: 5′-gtccaagtggggatctcctag | 319 |
| **EphrinB3** | F: 5′-gacacaggttttcggggtacagct  R: 5′-gagcctgtctactggaactcggc | 455 |
| **EphA1** | F: 5′-ttgccaactttgaccctagg  R: 5′-cttaaatccttgaatactgcag | 247 |
| **EphA2** | F: 5′-cccgagtgtccattcggctac  R: 5′-tcacttggtctttgagtcccag | 244 |
| **EphA3** | F: 5′-ggagttacgggattgtactctg  R: 5′-tggcaatggtgtcacaggagc | 389 |
| **EphA4** | F: 5′-agcgcttcatcagagagagcc  R: 5′-ggtccgggctagggttatact | 889 |
| **EphB1** | F: 5′-aagccccctacctcaaagttg  R: 5′-caccatccactctccatctcc | 352 |
| **EphB3** | F: 5′-gtagggtcaggtggggataag  R: 5′-gacagcaccaagggtaggcag | 217 |
| **EphB4** | F: 5′-cacccagcagcttgatcctg  R: 5′-accaggaccacacccacaac | 299 |
| **GADPH** | F: 5′-ctacatggtctacatgttccagta  R: 5′′-tgatggcatggactgtggtcat | 450 |

**Supplementary** **Table 7:** List of primers used for RT-PCR

| **Antibody** | **Source** | **Application** | **Dilution** |
| --- | --- | --- | --- |
| O4 | Sigma | Immunocytochemistry | 1:500 |
| Mbp | Millipore | Immunocytochemistry,  Immunohistochemistry Western Blot | 1:300 |
| Plp | Millipore | Western Blot | 1:5000 |
| A2B5 | Millipore | Immunocytochemistry | 1: 300 |
| EphrinB3 | Abcam (Ab2) | Immunocytochemistry  Immunoprecipitation  Neutralization assay  In vivo experiment | 1:1000  (2.5ug/ml) |
| EphrinB3 | R&D (Ab1) | Immunoprecipitation  Neutralization assay  In vivo experiment  Immunohistochemistry | 2.5 ug/ml |
| EphA4 | Abgent | Immunocytochemistry  Immunoprecipitation  Immunohistochemsitry | 1:500  1:2500 |
| EphB1 | Abgent | Immunocytochemistry  Immunoprecipitation | 1:500  1:2500 |
| EphB2 | Abgent | Immunocytochemistry  Immunoprecipitation | 1:500  1:2500 |
| EphB3 | Abgent | Immunocytochemistry  Immunoprecipitation | 1:500  1:2500 |
| Marcks | Sigma | Immunocytochemistry | 1:1000 |
| Rho A | Millipore | Rho A assay | 1:2500 |
| p-FAK | Cell Signaling | Immunoprecipitation | 1:3000 |
| t-FAK | Cell Signaling | Immunoprecipitation | 1:3000 |
| Nkx2.2 | Developmental Studies Hybridoma Bank | Immunocytochemistry  Immunohistochemsitry | 1:300 |
| MOG | Millipore | Immunohistochemistry | 1:500 |
| dMBP | Millipore | Immunohistochemistry | 1:500 |
| GFAP | Abcam | Immunohistochemistry | 1:500 |

**Supplementary** **Table 8:** List of antibodies used

**Supplementary** **Table 9 :** List of MS tissue samples

Type to enter text

|  | **Age (sex)** | **Disease duration and course** | **PM delay**  **hrs** | **HLA+ cell density in lesions (per x40 field)** |
| --- | --- | --- | --- | --- |
| **MS377 (MS1)** | 50 F | 23 yrs SPMS | 22 | 55.6 ± 10.1 |
| **MS378 (MS2)** | 53 M | 12 yrs PPMS | 15 | 51.2 ± 8.5 |
| **MS389 (MS3)** | 55 F | 4 yrs, PPMS | 14 | 74.0 ± 10.1 |
| **MS379 (MS4)** | 49, F | 16 yrs, SPMS | 21 | 74.1 ± 10.9 |
| **MS381 (MS5)** | 80, F | 37 yrs, SPMS | 7 | 54.6 ± 4.9 |
|  |  |  |  |  |
