# Supplementary figures and images for: Antibody-mediated neutralization of myelin-associated EphrinB3 accelerates CNS remyelination
Source: Acta Neuropathol. 2015 Dec 19;131(2):281–98. doi: 10.1007/s00401-015-1521-1 (PMC4713754; doi:10.1007/s00401-015-1521-1)

Supplementary Fig. 1

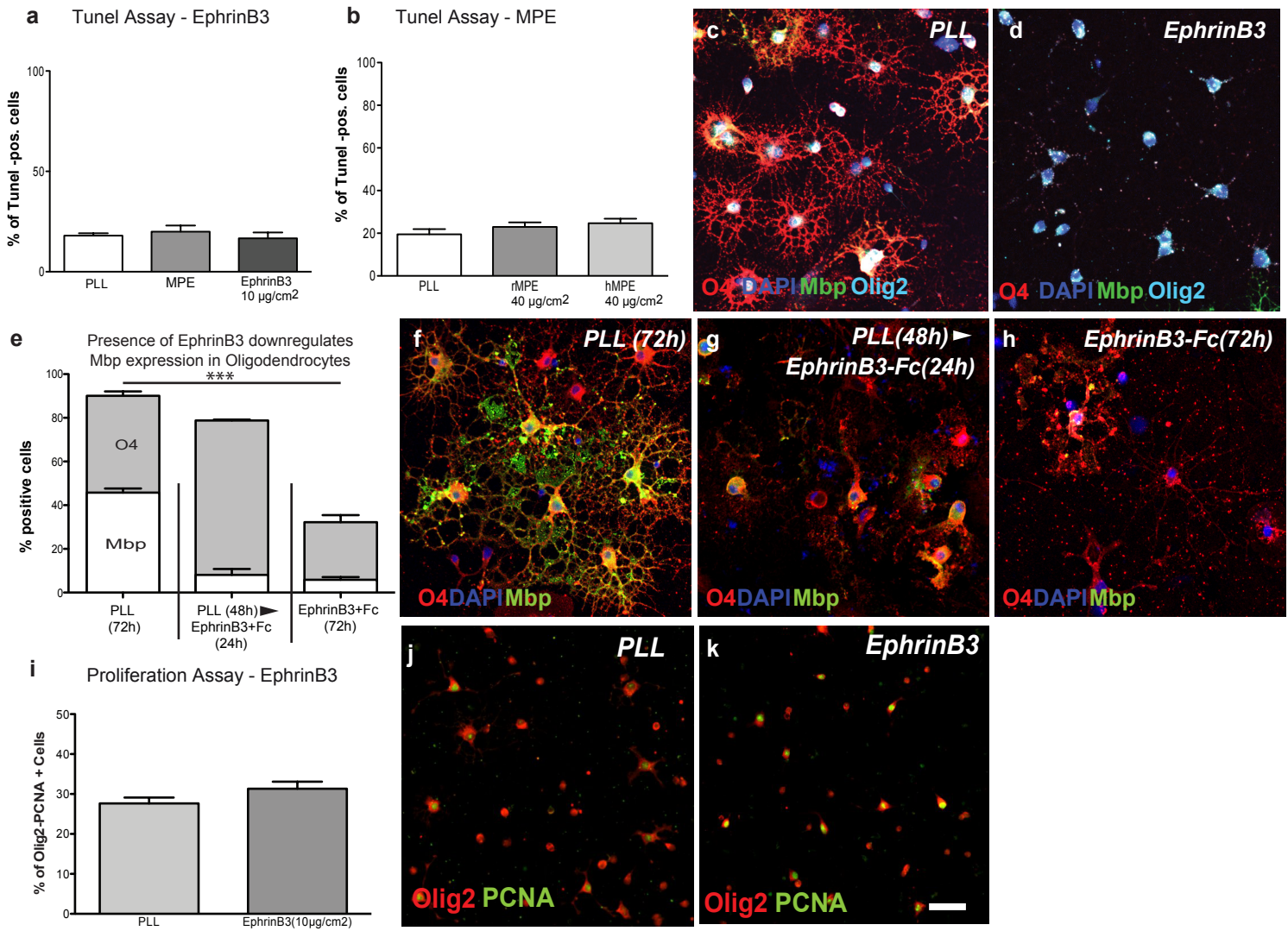

Supplement: Supplementary file 2 — Supplementary material 2 (PDF 16648 kb) [file 401_2015_1521_MOESM2_ESM.pdf]

**a**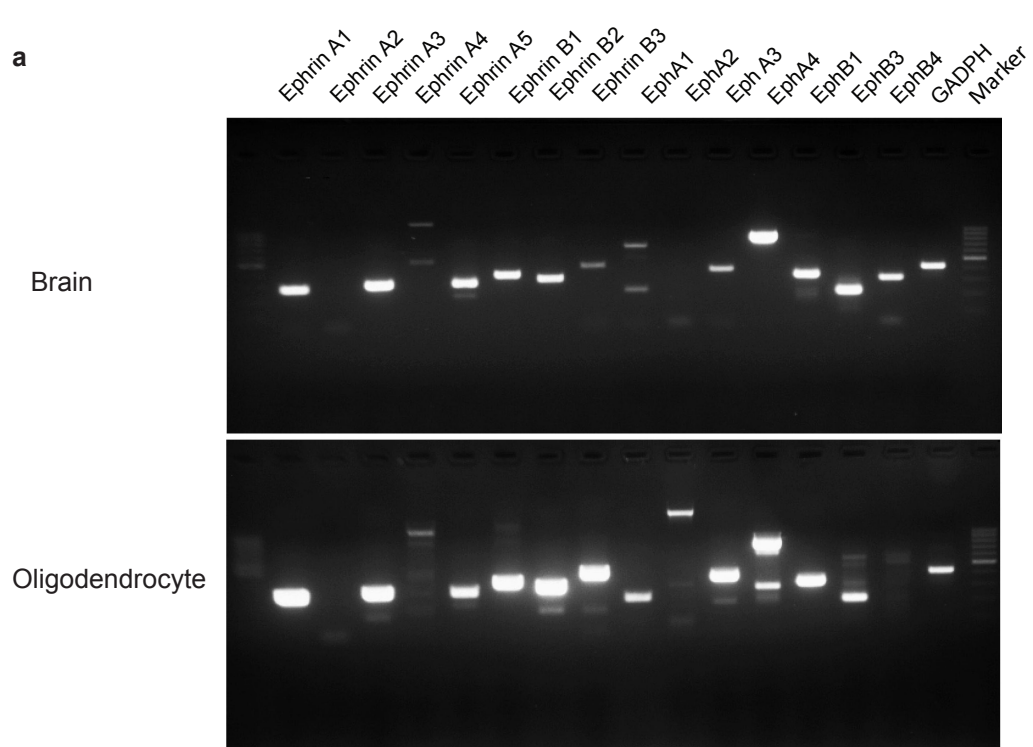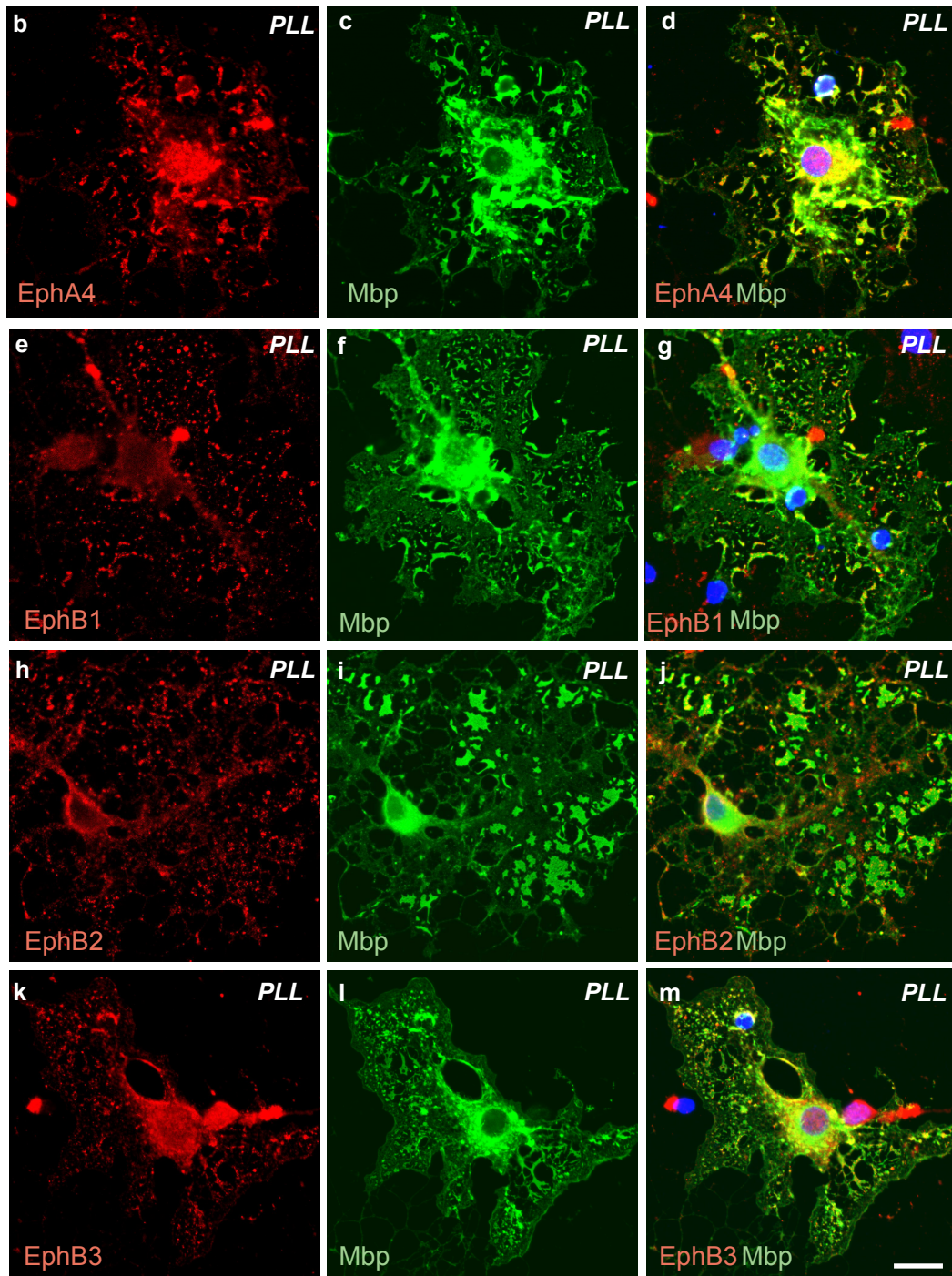

Supplementary Fig. 3

Supplement: Supplementary file 4 — Supplementary material 4 (PDF 15153 kb) [file 401_2015_1521_MOESM4_ESM.pdf]

Supplementary Fig. 4

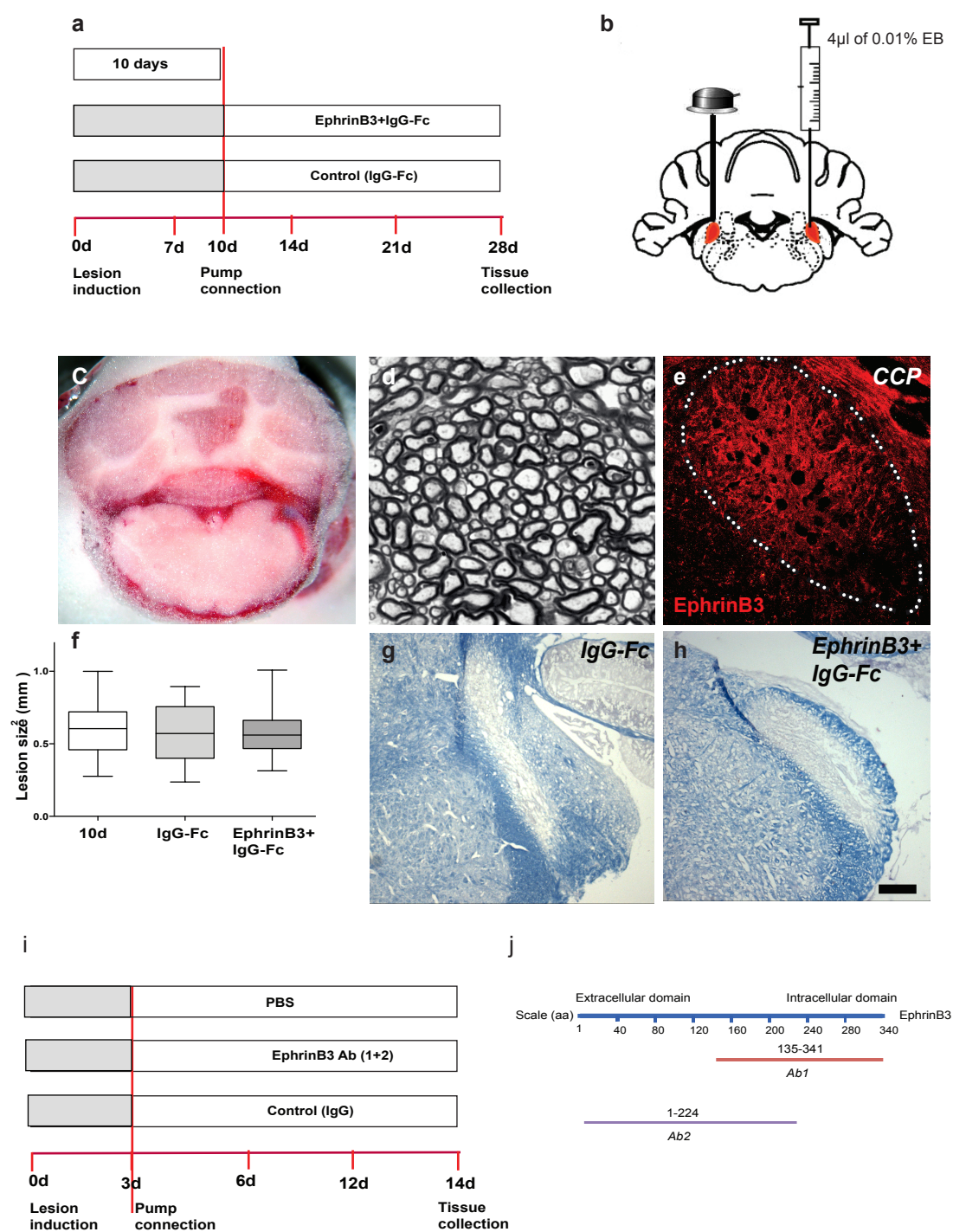

Supplement: Supplementary file 5 — Supplementary material 5 (PDF 12750 kb) [file 401_2015_1521_MOESM5_ESM.pdf]

Supplementary Fig. 5

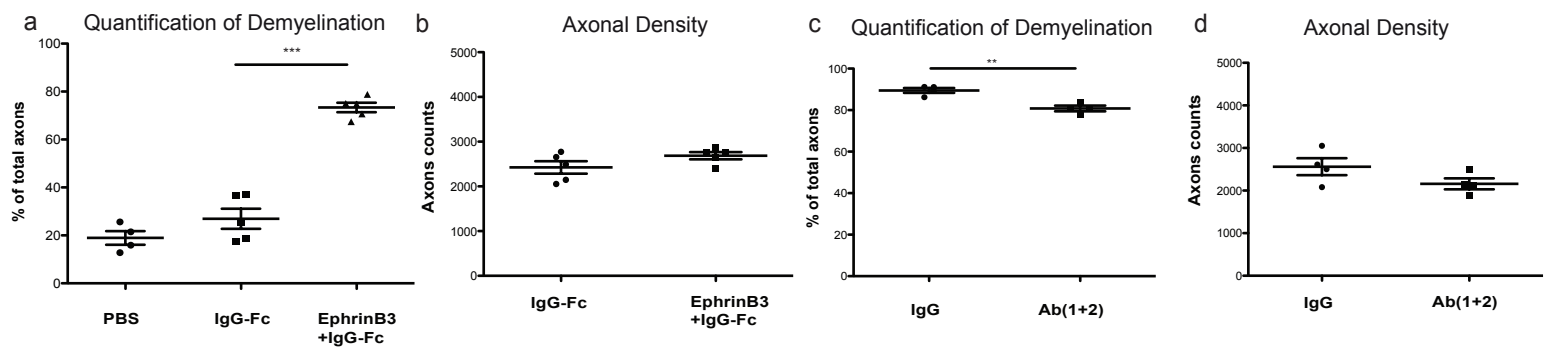

Supplement: Supplementary file 6 — Supplementary material 6 (PDF 305 kb) [file 401_2015_1521_MOESM6_ESM.pdf]
