# Supplementary material for: Antibody-mediated neutralization of myelin-associated EphrinB3 accelerates CNS remyelination
Source: Acta Neuropathol. 2015 Dec 19;131(2):281–98. doi: 10.1007/s00401-015-1521-1 (PMC4713754; doi:10.1007/s00401-015-1521-1)

**Supplementary Fig. 2**

**a** Experimental design and chromatograms of CM, High Q and S100 columns

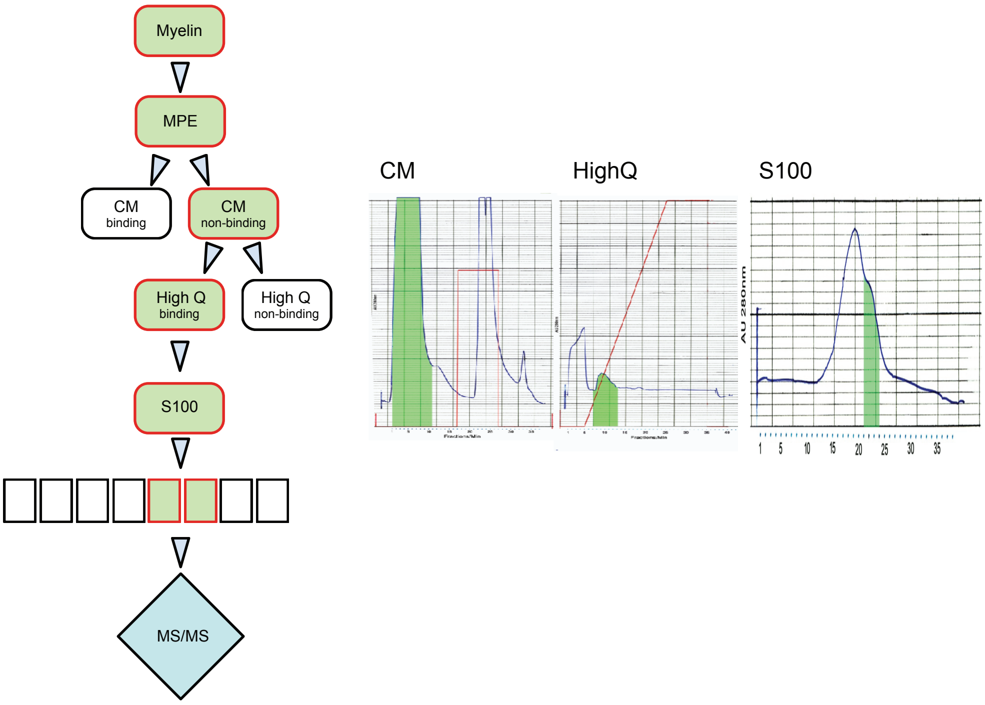


**b** Inhibition of OPC differentiation in the presence of enriched fractions

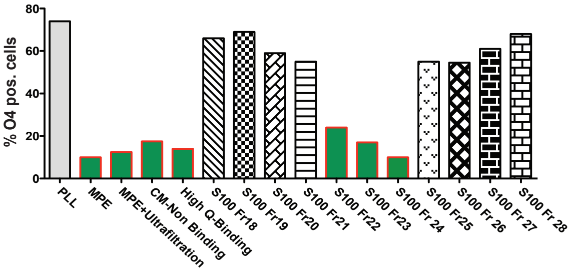


**C** Enrichment of EphrinB in inhibotry fractions

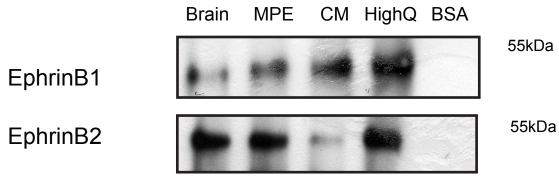

Supplement: Supplementary file 3 — Supplementary material 3 (PDF 2071 kb) [file 401_2015_1521_MOESM3_ESM.pdf]
