# Supplementary material for: Antibody-mediated neutralization of myelin-associated EphrinB3 accelerates CNS remyelination
Source: Acta Neuropathol. 2015 Dec 19;131(2):281–98. doi: 10.1007/s00401-015-1521-1 (PMC4713754; doi:10.1007/s00401-015-1521-1)

Supplementary Fig. 6

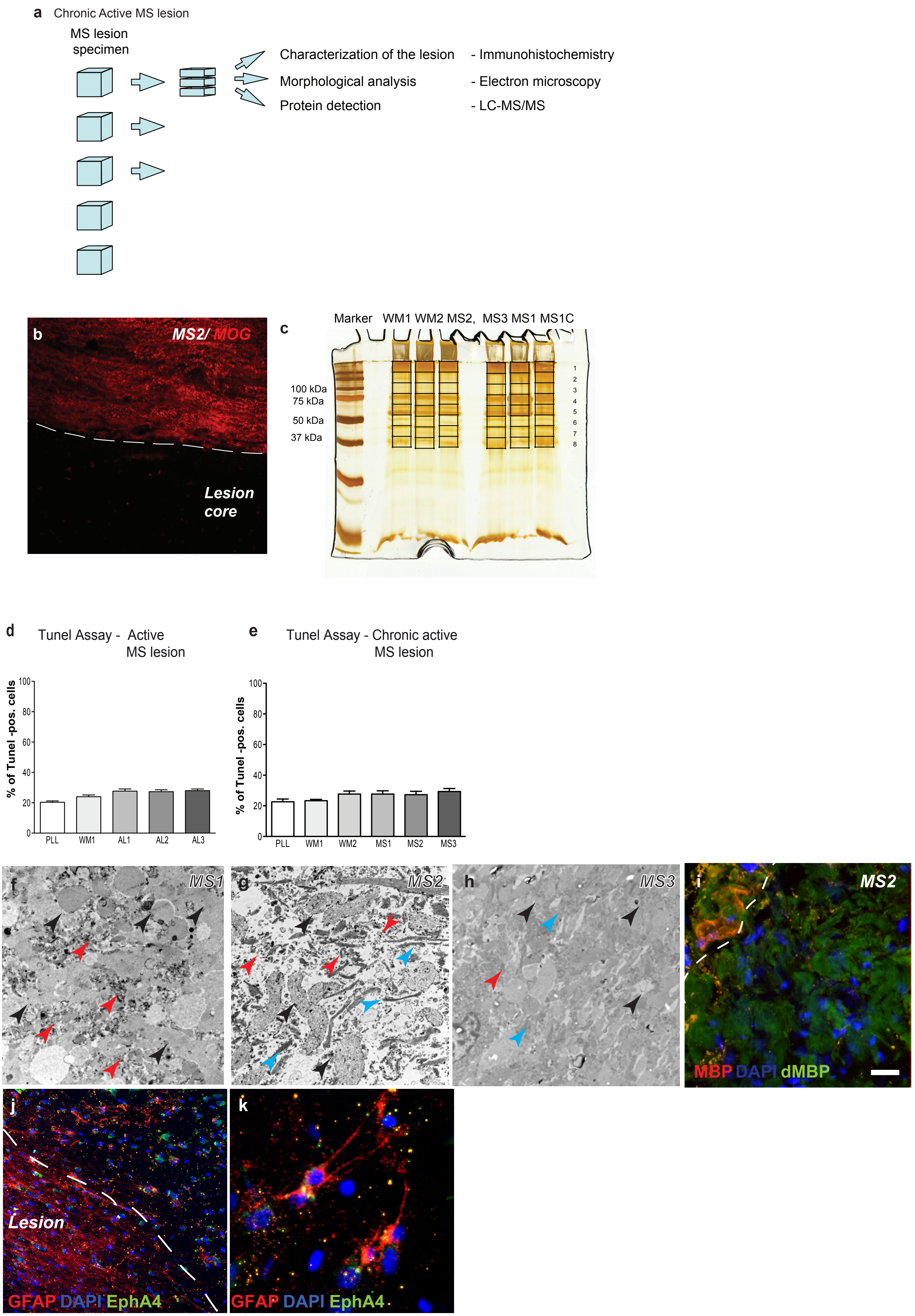

Supplement: Supplementary file 7 — Supplementary material 7 (PDF 15525 kb) [file 401_2015_1521_MOESM7_ESM.pdf]
